# Expeditive synthesis of trithiotriazine-cored glycoclusters and inhibition of *Pseudomonas aeruginosa* biofilm formation

**DOI:** 10.3762/bjoc.10.206

**Published:** 2014-08-25

**Authors:** Meriem Smadhi, Sophie de Bentzmann, Anne Imberty, Marc Gingras, Raoudha Abderrahim, Peter G Goekjian

**Affiliations:** 1 Laboratoire Chimie Organique 2 Glycochimie, Université de Lyon, ICBMS, UMR 5246 – CNRS, Université Claude Bernard Lyon 1, Bat. 308 –CPE Lyon, 43 Bd. du 11 Novembre 1918, 69622 Villeurbanne, France. Fax: +33-4-72448109; Tel: +33-4-72448183; 2 Université de Carthage, Faculté des sciences Bizerte, Tunisie; 3 Laboratoire d'Ingénierie des Systèmes Macromoléculaires, Institut de Biologie Structurale et Microbiologie, CNRS-Aix Marseille University, UMR7255, 31 Chemin Joseph Aiguier, 13402 Marseille Cedex 20, France; 4 Centre de Recherches sur les Macromolécules Végétales (CERMAV), UPR 5301 CNRS et Université Grenoble Alpes, BP53, 38041 Grenoble, France; 5 Aix-Marseille Université, CNRS, CINaM UMR 7325, 163 Avenue de Luminy 13288 Marseille, France

**Keywords:** antibiotic, biofilm, glycocluster, lectin, multivalency effect, multivalent glycosystems

## Abstract

Readily accessible, low-valency glycoclusters based on a triazine core bearing D-galactose and L-fucose epitopes are able to inhibit biofilm formation by *Pseudomonas aeruginosa*. These multivalent ligands are simple to synthesize, are highly soluble, and can be either homofunctional or heterofunctional. The galactose-decorated cluster shows good affinity for *Pseudomonas aeruginosa* lectin lecA. They are convenient biological probes for investigating the roles of lecA and lecB in biofilm formation.

## Introduction

*Pseudomonas aeruginosa* (PA) is an opportunistic human pathogen known to cause a variety of hospital-borne infections. It poses a severe threat to immunocompromised patients, as well as to those suffering from cystic fibrosis or cancer [1–3]. Its virulence is largely associated with multi-resistance to antibiotics, in particular due to the physical barrier created by surface-attached biofilms, thus limiting antibiotic penetration [4–6]. A challenging and useful task is therefore to develop novel strategies against PA colonies at this late stage of virulence. Among recent approaches, targeting biofilm formation or promoting its dissolution is thus particularly appealing. Because the formation of PA biofilm is a complex process partly mediated by the D-galactose-specific lectin lecA (PA-IL) [7–10] and the L-fucose-specific lectin lecB (PA-IIL) [11–13], lectin-carbohydrate interactions can provide a new target for pharmacological intervention. Further investigations of the specific functions played by these lectins in PA biofilm formation will provide useful understanding, and ultimately a means of prevention of PA virulence. The creative design of glycomimetics that can interfere or can modulate the bioactivity of these lectins in host recognition and adhesion in biofilm formation represents an attractive antibacterial strategy, as multivalent carbohydrate motifs on cell surfaces are known to mediate a broad range of cellular and tissue adhesion processes.

Carbohydrate recognition in biological systems is often based on the recognition of multiple epitopes through a synergistic and cooperative effect, called the ‘’glycocluster effect” [14–16]. It has been shown in a number of systems that multivalency effects can be exploited to obtain high-avidity synthetic ligands against various types of lectins in the form of glycoclusters [17], poly(glycomer)s [18–21], and glycodendrimers [22–24]. In regards to PA, *C*-fucosylpeptide dendrimers were shown to inhibit biofilm formation and to efficiently disperse established biofilms in both reference and hospital strains of PA [25–27]. Recently, galactosylated peptide dendrimers have shown a strong affinity for lecA while inhibiting or dispersing biofilms [28–29]. This anti-biofilm effect mediated by glycodendrimers validates a new approach to the control PA propagation and infection.

In this work and following those lines, we had in mind to develop simpler, lower molecular weight, and hydrosoluble multivalent ligands against lecA and lecB, able to exert useful biofilm inhibition and to provide useful tools for investigating the roles of lecA and lecB in the colonization process. Our investigations further aimed at concentrating a high density of proximate carbohydrate epitopes with limited degrees of freedom onto a sulfurated heteroaromatic scaffold as novel glycosylated asterisk ligands [30]. We have thus designed a simple, yet effective new family of multivalent glycosylated architectures built around a trithiotriazine core. Both homo- and heterobifunctional ligands are obtained by a straightforward preparative route, as an innovative approach. Additionally, isothermal titration calorimetry (ITC) and dynamic light scattering (DLS) helped to better understand lectin–ligand interactions between lecA or lecB and these trithiotriazine-based ligands.

## Results and Discussion

### Design of ligands

**Figure 1:**
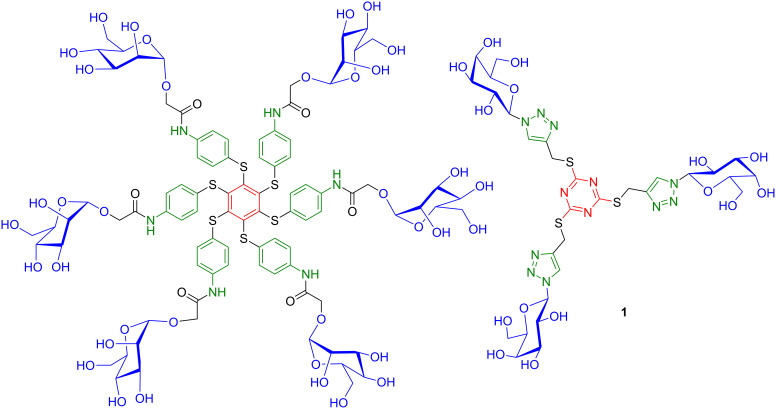
Previously reported low-valent glycoasterisk α-D-Man ligand based on a persulfurated benzene core [30] and currenly reported β-D-Gal compound **1**.

A previous study from our laboratories [30] has shown that low-valent glycoasterisk ligands based on a persulfurated benzene core [31–32] could have a dual role as a probe and as a ligand, due to their phosphorescence [33] and electrochemical properties [34] (Figure 1). They were also highly potent lectin aggregators. Among other aromatic glycoasterisks, Roy et al. described the synthesis of densely substituted hexaphenylbenzene glycoclusters [35].

In this work, we have designed a new family of low-valent glycoclusters based on a heteroaromatic core with the benefit of sulfur chemistry [36]. Sulfur facilitates the synthesis by providing a strong nucleophile and access to a thioether linkage under mild conditions, but it also enhances a number of potentially useful physical properties. For instance, polysulfuration of an aromatic core is known to significantly modify the HOMO–LUMO orbital energies, and thus change the redox potentials [31–34]. It also shifts the spectroscopic absorption and emission wavelengths and can lead to a phosphosrescence emission [33]. Additionally, an aza-aromatic core would improve water solubility by modifying π–π-interactions and by favoring hydrogen-bonding to water. These compounds also lack the hydrophobic peripheral benzene units of the previous glycoasterisk ligands. They were replaced with a methylene-triazole linker in order to increase water solubility and to modulate the degree of flexibility.

### Synthesis of ligands

**Scheme 1:**
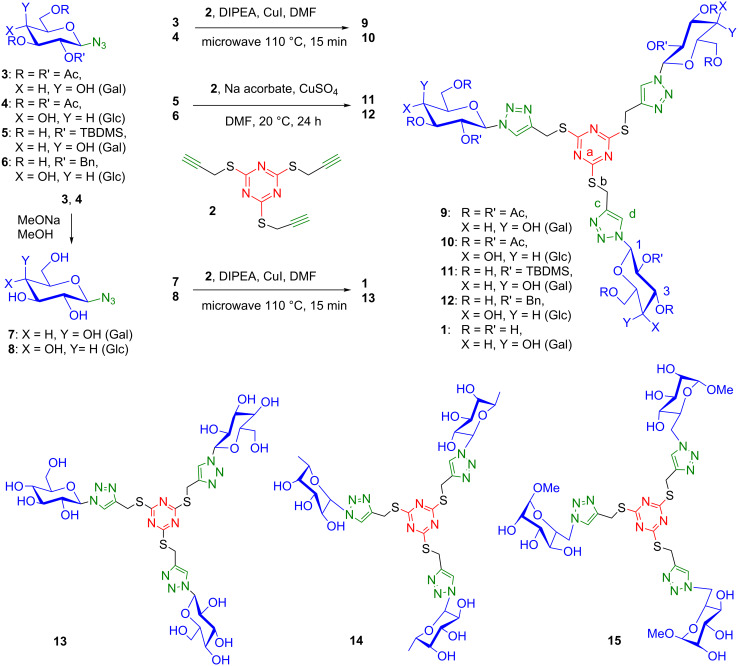
Synthesis of trivalent trithiotriazine-based glycoclusters.

The glycoclusters were prepared from the inexpensive trithiocyanuric acid (1,3-5-triazine-2,4,6-trithiol) as the heteroaromatic core (Scheme 1). Trisubstitution of the commercial trisodium salt with propargyl bromide ensured the facile preparation of 2,4,6-tris(propargylthio)-1,3,5-triazine (**2**) as a key precursor [37]. The glycosyl units were incorporated via Cu(I)-catalyzed Huisgen cycloaddition with protected or unprotected glycosyl azides.

We first investigated the Cu-catalyzed azide–alkyne cycloaddition (CuAAC) of acetyl protected β-D-galactopyranosyl azide **3** [38], to tris(propargylthio)triazine **2**, using CuI and diisopropylethylamine (DIPEA) in DMF under microwave irradiation at 110 °C for 15 min. It provided the peracetylated D-galactopyanosyl cluster **9** in 73% yield. The peracetyl D-glucopyranosyl cluster **10** was similarly obtained in 92% yield.

The deacetylation of the carbohydrate units proved to be problematic, as a result of the instability of the triazine system under either forcing or mild Zemplén deprotection conditions. The *tert-*butyldimethylsilyl-protected galactopyranosyl azide **5** was therefore prepared via the epoxidation of silylated D-galactal with dimethyldioxirane (DMDO) generated in situ in the presence of a phase-transfer catalyst, followed by treatment with NaN_3_ [39]. This afforded the silyl-protected D-galactose trithiotriazine–triazole glycocluster **11** under CuSO_4_/sodium ascorbate-catalyzed cycloaddition conditions [40] (20 °C, 24 h), in a satisfactory 87% yield. The benzyl protected D-glucose glycocluster **12** was similarly prepared from tri-*O*-benzyl-β-D-glucopyranosyl azide **6** [39] in 92% yield. The removal of the silyl groups with TBAF led to complete degradation of the scaffold. Ammonium fluoride in THF or trifluoroacetic acid also led to the fragmentation of the cluster core, which preceded complete deprotection of the carbohydrate groups. We were unable to obtain the deprotected glycoclusters by this route.

We therefore investigated a direct route to the glycoasterisks using unprotected azidosugars, thus avoiding the final deprotection step. The unprotected azidosugars were obtained by straightforward deprotection of the corresponding acetyl-protected azides [38]. The trivalent glycoclusters decorated with D-galactose, **1**, D-glucose, **13**, and L-fucose, **14**, epitopes were thus obtained directly in 53%, 50%, and 44% yields, respectively, after reversed-phase chromatography. Methyl 6-azido-6-deoxy-α-D-mannoside was similarly coupled as a less expensive isostere of L-fucose [41]. The tris 6-*C*-(6-deoxy-D-mannosyl) cluster **15** was thus obtained in 47% yield. The cycloaddition conditions were optimized using 3.3 equiv of glycosyl azide [39] and one equivalent of tris(propargylthio)triazine **2** in DMF, catalyzed by CuI and DIPEA under microwave irradiation. The incorporation of three carbohydrate residues was established unambiguously by ESIMS, ^1^H NMR, ^13^C NMR, and HMBC analysis, in particular based on the symmetry of the molecule, and on the lack of signals corresponding to the residual alkynes in the NMR and MS. The connectivity was established thanks to HMBC ^3^*J* proton–carbon correlations between the anomeric proton of the sugar and the triazole methine carbon (H-1–C-d), between the trizaole methine carbon and the thiomethylene protons (C-d–H-b), and between the thiomethylene protons and the triazine carbon (H-b–C-a). Despite the moderate yields, these products are readily accessible, being easy to purify, simple to characterize, and able to be produced on a relatively large scale.

**Scheme 2:**
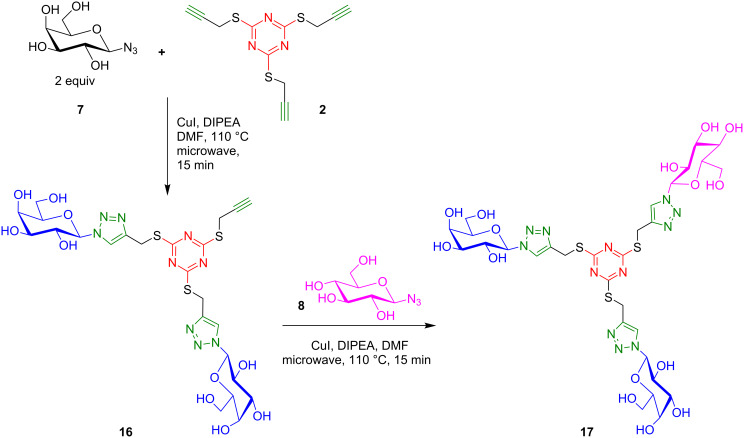
Synthesis of mixed triazine-based glycoclusters.

The current process also offers the possibility of synthesizing mixed glycoclusters. Reducing the number of equivalents of glycosyl azide **7** to 2 equiv in the presence of CuI and DIPEA in DMF at 110 °C under microwave irradiation provided a statistical mixture with the bivalent cluster as the major product. The bis-D-galactosyl cluster **16** was thus isolated in 34% yield. A second [3 + 2] cycloaddition with a different glycoside, such as D-glycopyranosyl azide **8**, under the same conditions, provided for example the mixed Gal_2_-Glc triazine cluster **17** (Scheme 2).

The efficient conjugation of unprotected glycosyl azides to trithiotriazine **2** thus provides convenient access to low valency mono- or heterobifunctional glycoclusters. As expected, they display excellent aqueous solubility due of the combination of a dendritic polyheterocyclic architecture and carbohydrate epitopes.

### Biophysical studies

**Figure 2:**
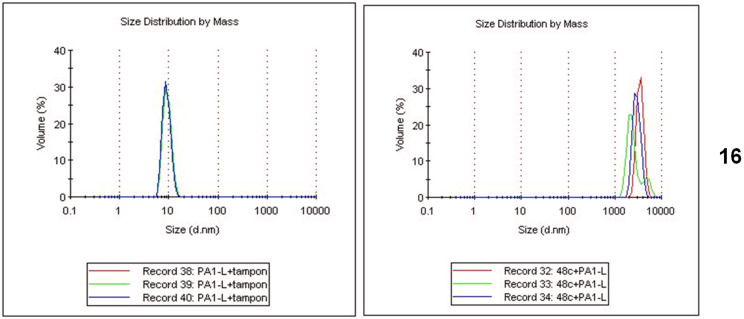
Dynamic light scattering experiments of bis-D-galactosyl proparyl cluster **16** with lecA. Distribution by mass for lecA + buffer (left) and lecA + **16** (200 μM, right) at 3 minute intervals. Additional experiments can be found in Supporting Information File 1.

Dynamic light scattering experiments (DLS) were performed on the trivalent and divalent galactose-substituted clusters **1**, **16** and **17**, as well as the glucose-substituted cluster **13** as a negative control. The results show that of the four clusters, only the divalent bis-D-Gal propargyl cluster **16** induces rapid aggregation of lecA (Figure 2 and Supporting Information File 1). Although such results should not be over-interpreted, they confirm that two epitopes are sufficient for aggregation, and suggest that additional hydrophobic and hydrophilic interactions play a role. The inability of these systems to efficiently aggregate lectins is in stark contrast to the hexavalent benzene cluster [30], which may be attributed to differences in rigidity and hydrophobicity between the two systems [25]. It thus appears that the direct diaryl sulfide bridge presents a more optimal degree of semi-rigidity.

**Figure 3:**
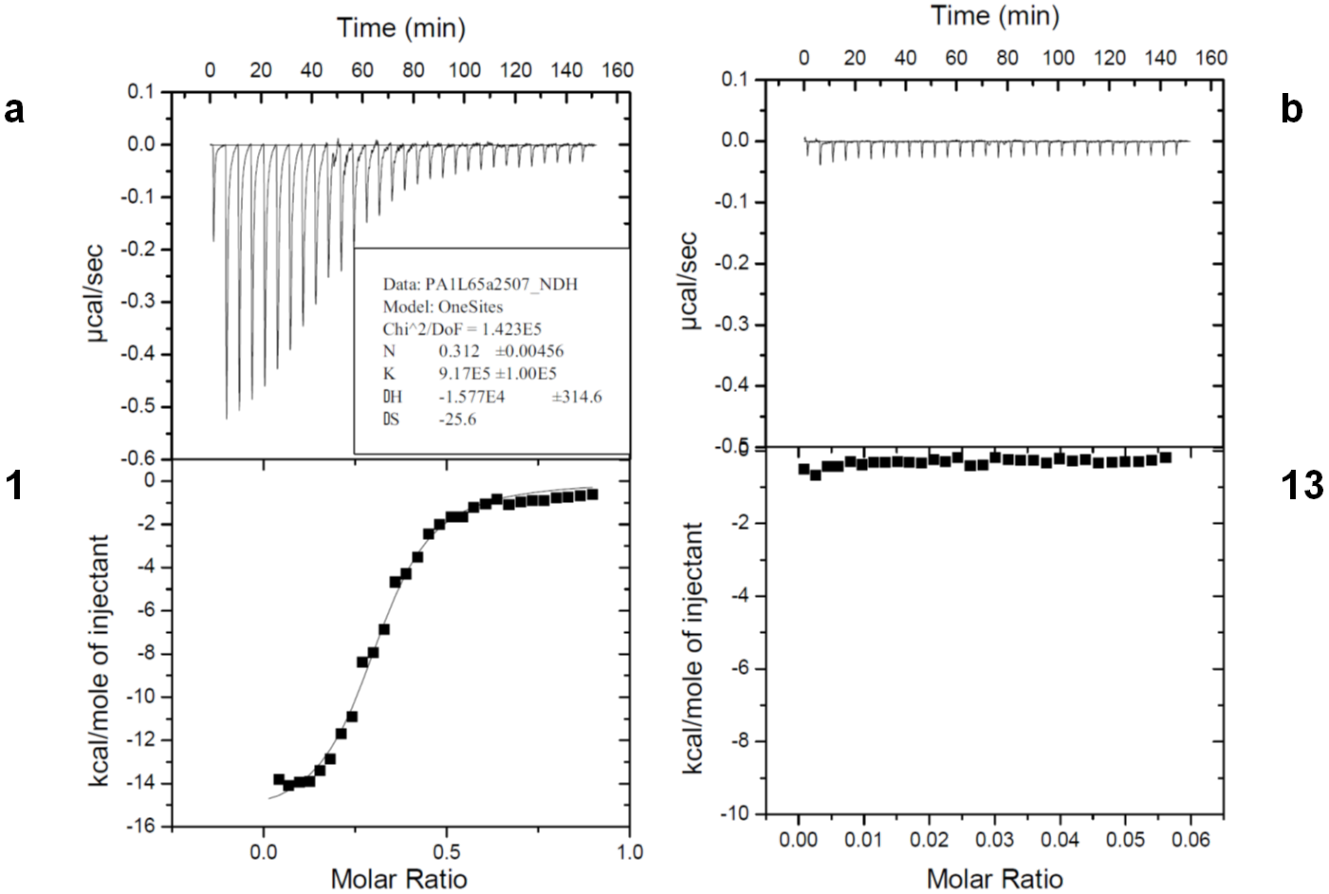
Typical ITC measurements representing the raw ITC data (top) and integrated titration curves (bottom) for the binding to lecA of a) tris-D-galactosyl triazine cluster **1**, and b) tris D-glucosyl glycocluster **13** (negative control).

The affinities of the designed glycoconjugates with lecA and lecB were determined by isothermal titration calorimetry (ITC) by addition of the ligands to a solution of lectin (Figure 3). Dissociation constants (*K*_d_) and thermodynamic parameters (Δ*G*, Δ*H*, −*T*Δ*S*) are listed in Table 1, together with the experimental binding stoichiometry (*n*), defined as the number of glycocluster ligands per monomer of lectin.

The trivalent tris-galacosylated glycoconjugate **1** displays a good affinity and a *K*_d_ value of 1.09 µM, compared to 94 µM for the monovalent reference, methyl β-D-galactoside (Table 1). The stoichiometry indicates that each cluster binds to three lecA sites. The tris-glucosylated cluster **13** was used as a negative control with nearly identical physical properties, and showed no affinity for the lectin, confirming that the recognition is epitope-specific. The bivalent clusters containing two galactose residues **16** and **17** have similar binding constants, although the mixed cluster **17** containing two D-galactose and one D-glucose residues provided better ITC titration curves and more rational *n* values than the bis D-galactosyl monopropargyl cluster **16**, which may reflect precipitation of the lectin-cluster complex during the ITC experiment in the latter case, based on the DLS results above.

**Table 1:** Thermodynamic parameters of glycoclusters upon binding to lecA by ITC^a^.

cmpd	val.	*n*^b^	Δ*H* kJ/mol	−*T*Δ*S* kJ/mol	Δ*G* kJ/mol	*K*_d_ µM	β/N^c^

β-D-GalOMe^d^	1	0.8	−42.8	19.8	−23.0	94	1
Gal_3_-tzn, **1**	3	0.31	−66.0	31.9	−34.1	1.09	29
Gal_2_Glc-tzn, **17**	2	0.54	−51.0	19.7	−31.3	3.4	14
Gal_2_Pg-tzn, **16**	2	0.79	−47.6	17.2	−30.5	4.4	11
Glc_3_-tzn, **13**	3				<0		

^a^*T* = 298 K. ^b^Stoichiometry. ^c^Improvement in affinity relative to the methyl glycoside, divided by the valency. ^d^Data from reference [50]. Pg = propargyl; tzn = tris(triazolylmehylthio)triazine.

The observed β/N values in Table 1, which reflect the relative affinity per unit sugar, are 29 for the trivalent cluster and in the range of 12 for the bivalent clusters. These values most likely reflect sub-site binding by the heterocyclic rings. Indeed, the divalent clusters **16** and **17** show a relatively less unfavorable entropy contribution, compared to methyl β-D-galactopyranoside, which is consistent with the contribution of additional hydrophobic interactions. No chelate binding is expected in this first generation cluster, as the arm length is well below the 29 Å distance between sugar binding sites [7]. Not unexpectedly, several reported multivalent clusters have achieved higher affinities, yet the values observed here fall within the range obtained with far more complex multivalent systems [28,42–50]. The β-fucoside-containing trivalent cluster **14** was also tested by ITC and a *K*_d_ of 50 μM was obtained, which is significantly higher than the *K*_d_ for α-MeFuc (0.43 μM) [41] (data not shown). This confirms that lecB has lower affinity for β-fucosides than for the α-anomers, but the trimeric β-fucoside cluster **14** still demonstrated reasonable binding. The 6-deoxymannose isostere **15** was not tested, in view of the low affinity of the β-fucose epitopes. These clusters thus represent a readily accessible, highly soluble, and convenient tool for the investigation of the role of lecA and lecB in the formation of biofilms by *Pseudomonas aeruginosa*.

### Inhibition of biofilm formation

While the expectation that glycoclusters with high affinity to lecA and lecB should inhibit biofilm formation is now a common design hypothesis, it is nonetheless important to show whether individual synthetic clusters do so in fact. This has only been done in a limited number of examples [9–10], perhaps due to lack of solubility, lack of availability, or other reasons. The response of PA biofilms to different clusters is not necessarily directly correlated to their affinity, as many other factors may intervene, and the accumulation of biofilm data will therefore be an important factor in our understanding of this complex process.

The *P. aeruginosa* adherence assay was performed in 24 well microplates. Biofilms were obtained after 24 h of incubation at 30 °C in LB medium alone or in the presence or galactose, fucose, or glucose (control)-substituted trivalent clusters and stained with crystal violet (CV).

**Figure 4:**
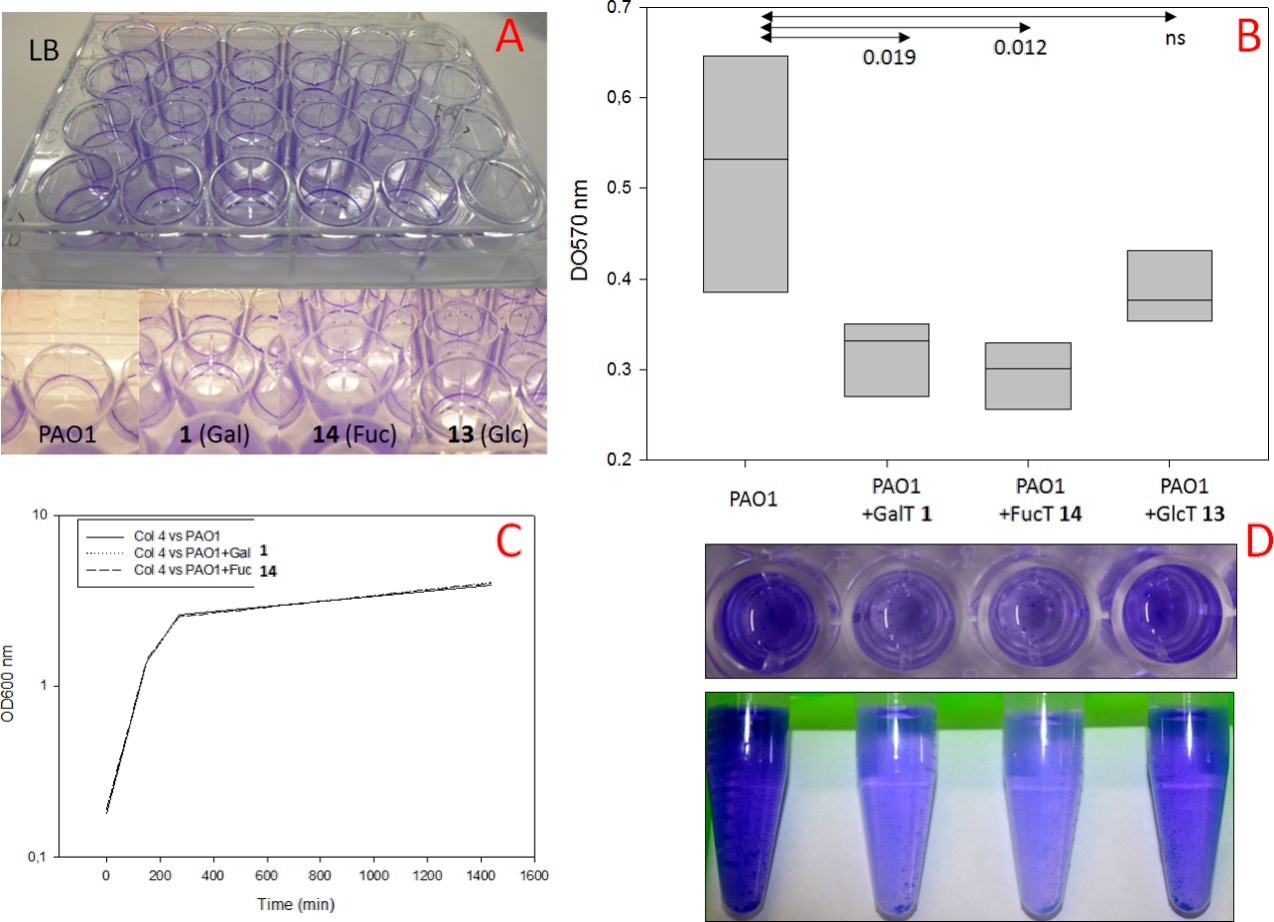
Inhibition of PAO1 biofilm formation by D-galactose cluster **1**, L-fucose cluster **14**, and D-glucose cluster **13** (negative control). A) Biofilm growth assay in LB medium. B) Statistical analysis (above, *n* = 5, duplicate UV measurements) of ethanol-solubilized biofilm recovered from each well (below). C) PAO1 growth inhibition control test.

A statistically significant reduction in biofilm formation was observed at 5 mM concentration of either the galactose- or the fucose-bearing cluster, **1** and **14**, respectively, as compared to the glucose-bearing cluster, **13**, or absence of cluster (Figure 4). To check that differences observed were not due to bacterial growth defect in the presence of clusters, a growth inhibition control experiment was performed (Figure 4C). No growth defect was observed, further confirming that observed reduction of biofilm formation in the presence of the galactose or the fucose-bearing clusters is due to potential effects on *P. aeruginosa* lectins.

## Conclusion

We have developed a convenient synthesis of simple, low-valency glycoclusters. These compounds have good solubility, are readily accessible, and are easy to purify and to characterize. The presence of the sulfur provides beneficial structural and synthetic elements, and the heterocyclic systems improve solubility and may potentially lead to better pharmacodynamic properties for eventual biological applications. They show good affinities for the lectins, comparable to more complex multivalent systems. The recognition is sugar-specific, as the corresponding D-glucose glycocluster shows no affinity for the lectin, and can thus be used as a negative control. Both the D-galactose and L-fucose clusters are able to inhibit biofilm formation. These compounds therefore provide convenient tools for further investigation of lectin-mediated processes in *P. aeruginosa* biofilm formation.

## Experimental

**2,4,6-tris(1-(β-D-galactopyranosyl)triazol-4-ylmethylthio)-1,3,5-triazine (1).** A solution of compound **2** (22 mg, 0.073 mmol, 1 equiv), β-D-galactopyranosyl azide (59.7 mg, 0.294 mmol, 4 equiv), CuI (0.022 mmol, 4.2 mg, 0.3 equiv) and DIPEA (0.2 mL, 15 equiv) in DMF (2 mL) was heated under microwave irradiation for 15 minutes at 110 °C. The reaction mixture was concentrated in vacuo and the residue was purified by C18 chromatography (Combiflash, Grace Reveleris C18 RP 4g Cartridge, H_2_O/MeOH gradient). Yield = 53%. TLC (C18; MeOH/H_2_O 1:1). *R*_f_ = 0.42. [α]_D_ +14.5 (*c* 1, H_2_O); IR (neat) υ = 3287.6 cm^−1^ (OH) 1474.3 (triazole); ^1^H NMR (400 MHz, DMSO-*d*_6_) δ 8.20 ppm (s, 3H, H-d), 5.45 (d, *J =* 9.2 Hz, 3H, H-1), 5.22 (d, *J =* 6.0 Hz, 3H, OH-2), 5.01 (d, *J =* 5.7 Hz, 3H, OH-3), 4.69 (t, 3H, OH-6), 4.64 (d, *J =* 5.4 Hz, 3H, OH-4), 4.52 (s, 6H, H-b), 4.01 (ddd, *J =* 9.3 Hz, *J =* 9.2 Hz, *J =* 6.0 Hz, 3H, H-2), 3.76 (br dd, *J =* 5.7, 3.4 Hz, 3H, H-4), 3.69 (br dd, *J =* 6.1, *J =* 6.1 Hz, 3H, H-5), 3.55–3.44 (m, 9H, H-3, H-6, H-6’); ^13^C NMR (100 MHz, DMSO-*d*_6_) δ 178.5 ppm (C-a), 142.4 (C-c), 122.5 (C-d), 88.1 (C-1), 78.4 (C-5), 73.6(C-3), 69.2 (C-2), 68.4 (C-4), 60.4 (C-6), 24.6 (C-b); HRMS–ESI (*m*/*z*): [M + H]^+^ calcd for C_30_H_43_N_12_O_15_S_3_, 907.2170; found, 907.2127; (*m*/*z*): [M + Na]^+^ calcd for C_30_H_43_N_12_NaO_15_S_3_, 929.1980; found, 929.1947.

**2,4,6-tris(1-(β-D-glucopyranosyl)triazol-4-ylmethylthio)-1,3,5-triazine (13).** A solution of compound **2** (18.1 mg, 0.062 mmol, 1 equiv), β-D-glucopyranosyl azide (51 mg, 0.25 mmol, 4 equiv), CuI (3.5 mg, 0.3 equiv) and DIPEA (0.74 mmol, 0.16 mL, 15 equiv) in DMF (1 mL) was heated under microwave irradiation for 15 minutes at 110 °C. The reaction mixture was concentrated in vacuo and the residue was purified by C18 chromatography (Combiflash, Grace Reveleris C18 RP 4g Cartridge, H_2_O/MeOH gradient). Yield = 50%. TLC (C18; MeOH/H_2_O 1:1). *R*_f_ = 0.47. [α]_D_ −2.0 (*c* 0.46, H_2_O); ^1^H NMR (300 MHz, DMSO-*d*_6_) δ 8.25 ppm (s, 3H, H-d), 5.51 (d, *J =* 9.3Hz, 3H, H-1), 5.38 (d, *J =* 6.0Hz, 3H, OH-2), 5.27 (d, *J =* 4.9, 3H, OH-3), 5.14 (d, *J =* 5.5, 3H, OH-4), 4.63 (t, *J =* 5.6, 3H, OH-6), 4.52 (s, 6H, H-b), 3.77–3.66 (m, 6H, H-2, H-6), 3.46–3.32 (m, 6H, H-3, H-5, H-6’), 3.25–3.15 (m, 3H, H-4); ^13^C NMR (75 MHz, DMSO-*d*_6_) δ 178.6ppm (C-a), 142.4 (C-c), 122.9 (C-d), 87.5 (C-1), 79.9 (C-3), 76.9 (C-5), 72.0 (C-2), 69.5 (C-4), 60.7 (C-6), 24.6 (C-b); HRMS–ESI (*m*/*z*): [M + H]^+^ calcd for C_30_H_43_N_12_O_15_S_3_, 907.2132; found, 907.2127; (*m*/*z*): [M + Na]^+^ calcd for C_30_H_43_N_12_NaO_15_S_3_. 929.1943; found, 929.1947.

**2,4,6-tris(1-(β-L-fucopyranosyl)triazol-4-ylmethylthio)-1,3,5-triazine (14).** Compound **2** (132.9 mg, 0.458 mmol, 1 equiv), β-L-fucopyranosyl azide (345.6 mg, 1.82 mmol, 4 equiv), CuI (26 mg, 0.13 mmol, 0.3 equiv) and DIPEA (1.13 mL, 16.8 mmol, 5 equiv) in DMF (2 mL) was heated under microwave irradiation for 15 minutes at 110 °C. The reaction mixture was concentrated in vacuo and the residue was purified by C18 chromatography (Combiflash, Grace Reveleris C18 RP 4g Cartridge, H_2_O/MeOH gradient). Yield = 44%. TLC (C18; MeOH/H_2_O 1:1) *R*_f_ = 0.5. [α]_D_ +5.2 (*c* 0.17; H_2_O); ^1^H NMR (400 MHz, DMSO-*d*_6_) δ 8.18 ppm (s, 3H, H-d), 5.44 (d, *J =* 9.2 Hz, 3H, H-1), 5.22 (d, *J =* 6.0 Hz, 3H, OH-2), 5.00 (d, *J =* 5.5 Hz, 3H, OH-3), 4.70 (d, *J =* 5.4 Hz, 3H, OH-4), 4.52 (s, 6H, H-b), 4.03–3.94 (m, 3H, H-2), 3.88 (br. q, *J =* 6.3 Hz, 3H, H-5), 3.57–3.51 (m, 6H, H-4, H-3), 1.13 (d, *J =* 6.4 Hz, 9H, CH_3_); ^13^C NMR (100 MHz, DMSO-*d*_6_) δ 178.1ppm (C-a), 142.0 (C-c), 122.0 (C-d), 87.6 (C-1), 73.6 (C-3), 73.0 (C-5), 71.0 (C-4), 68.5 (C-2), 24.3 (C-b), 16.3 (CH_3_); HRMS–ESI (*m*/*z*): [M + H]^+^ calcd for C_30_H_43_N_12_O_12_S_3_, 859.2299; found, 859.2280 ; (*m*/*z*): [M + Na]^+^ calcd for C_30_H_42_N_12_NaO_12_S_3_, 881.2096; found, 881.2099.

**2,4-bis(1-(β-D-galactopyranosyl)triazol-4-ylmethylthio)-6-(prop-2-ynylthio)-1,3,5-triazine (16).** A solution of compound **2** (35.9 mg, 0.123 mmol, 1 equiv), β-D-galactopyranosyl azide (50.6 mg, 0.240 mmol, 2 equiv), CuI (0.036 mmol, 7 mg, 0.3 equiv) and DIPEA (1.84 mmol, 0.32 mL, 15 equiv) in DMF (2 mL) was heated under microwave irradiation for 15 minutes at 110 °C. The reaction mixture was concentrated in vacuo and the residue was purified by C18 chromatography (Combiflash, Grace Reveleris C18 RP 4g Cartridge, H_2_O/MeOH gradient). Yield = 34%. [α]_D_ +4.3 (c 0.36, H_2_O); ^1^H NMR (400 MHz, DMSO-*d*_6_) δ 8.18 ppm (s, 2H, H-d), 5.44 (d, *J =* 9.2 Hz, 2H, H-1), 5.23 (d, *J =* 6.0 Hz, 2H, OH-2), 5.02 (d, *J =* 5.6 Hz, 2H, OH-3), 4.70 (t, *J =* 5.6 Hz, 2H, OH-6), 4.67 (d, *J =* 5.3 Hz, 2H, OH-4), 4.58–4.50 (AB, *J* ~ 15.0 Hz, 4H, H-b), 4.05–4.03 (m, 2H, H-e), 4.0 (ddd, *J =* 9.3 Hz, *J =* 9.2 Hz, *J =* 6.0 Hz, 2H, H-2), 3.76 (br dd, *J =* 5.3 Hz, *J =* 3.5 Hz, 2H, H-4), 3.69 (br t, *J =* 6.0 Hz, 2H, H-5), 3.54–3.46 (m, 6H, H-3, H-6, H-6’), 3.23 (t, *J =* 2.5 Hz, 1H, H-g);^13^C NMR (100 MHz, DMSO-*d*_6_) δ 178.4 ppm (C-a), 177.8 (C-a’), 142.4 (C-c), 122.3 (C-d), 87.9 (C-1), 79.5 (C-f), 78.2 (C-5), 73.8 (C-g), 73.4 (C-3), 69.1 (C-2), 68.3 (C-4), 60.2 (C-6), 24.4 (C-b), 18.4 (C-e); HRMS–ESI (*m*/*z*): [M + H]^+^ calcd for C_24_H_32_N_9_O_10_S_3_, 702.1461; found, 702.1429; (*m*/*z*): [M + Na]^+^ calcd for C_24_H_31_N_9_NaO_10_S_3_, 724.1277; found, 724.1248.

**2,4-bis(1-(β-D-galactopyranosyl)triazol-4-ylmethylthio)-6-(1-(β-D-glucopyranosyl)triazol-4-ylmethylthio)-1,3,5-triazine (17).** A solution of the bis-Gal triazine cluster **16** (12.2 mg, 0.017 mmol, 1 equiv), β-D-glucopyranosyl azide (5.3 mg, 0.026 mmol, 1.5 equiv), CuI (1 mg, 0.3 equiv) and DIPEA (0.043 mL, 15 equiv) in DMF (1mL) was heated under microwave irradiation for 15 minutes at 110 °C. The reaction mixture was concentrated in vacuo and the residue was purified by C18 chromatography (Combiflash, Grace Reveleris C18 RP 4g Cartridge, H_2_O/MeOH gradient). Yield = 30%. [α]_D_ −1.2 (*c* 0.1, H_2_O); ^1^H NMR (500 MHz, D_2_O) δ 8.20 ppm (s, 2H, Gal H-d), 8.16 (s, 1H, Glc H-d), 5.70 (d, *J =* 9.2 Hz, 1H, Glc H-1), 5.65 (d, 2H, *J =* 9.2 Hz, Gal H-1), 4.40 (s, 6H, H-b), 4.19 (t, *J =* 9.5 Hz, 2H, Gal H-2), 4.09 (d, *J =* 3.2 Hz, 2H, Gal H-5), 4.01–3.94 (m, 3H, Gal H-4,Glc H-2), 3.88 (dd, *J =* 9.3 Hz, *J =* 3.2 Hz, 2H, Gal H-3), 3.77 (d, *J =* 12.1 Hz, 2H, Gal H-6), 3.76 (d, *J =* 12.1 Hz, 2H, Gal H-6’), 3.74 (d, *J =* 11.6 Hz, 1H, Glc H-6), 3.73 (d, *J =* 11.6 Hz, 1H, Glc H-6’), 3.71–3.67 (m, 2H, Glc H-3, Glc H-4), 3.62 (t, *J =* 9.4 Hz, 1H, Glc H-5); ^13^C NMR (125 MHz, D_2_O) δ 178.7 ppm (C-a), 144.4 (C-c), 123.4 (C-d), 87.1 (Gal C-1), 87.4 (Glc C-1), 78.9 (Glc C-4), 78.3 (Gal C-4), 75.9 (Glc C-3), 73.0 (Gal C-3), 72.3 (Glc C-2), 69.8 (Glc C-5), 68.6 (Gal C-5), 62.5 (Glc C-6), 60.8 (Gal C-6), 24.4 (Cb); HRMS–ESI (*m*/*z*): [M + Na]^+^ calcd for C_30_H_42_N_12_NaO_15_S_3_, 929.1916; found, 929.1947.

## Supporting Information

Full 1D and 2D NMR spectra of compounds **1**, **2**, and **9**–**17**; experimental procedures for ITC and biofilm inhibition studies, and for the synthesis of tris propargyl precursor **2**, protected clusters **9**–**12,** and 6-*C*-mannose cluster **15**; additional DLS and ITC spectra, additional biofilm quantification information.

File 1 Experimental procedures, characterization checklist and NMR, DLS and ITC data.

## References

[1] Ramos J-L (2004). Pseudomonas.

[2] Bodey G P, Elting L S, Narro J, Koller C, O'Brien S, Estey E, Benjamin R (1993). J Antimicrob Chemother.

[3] Mendelson M H, Gurtman A, Szabo S, Neibart E, Meyers B R, Policar M, Cheung T W, Lillienfeld D, Hammer G, Reddy S (1994). Clin Infect Dis.

[4] Wagner V E, Iglewski B H (2008). Clin Rev Allergy Immunol.

[5] Lister P D, Wolter D J, Hanson N D (2009). Clin Microbiol Rev.

[6] Penha Escudeiro B M, Baracho Marques E C (2012). Pseudomonas Aeruginosa: Symptoms of Infection, Antibiotic Resistance and Treatment.

[7] Imberty A, Wimmerová M, Mitchell E P, Gilboa-Garber N (2004). Microbes Infect.

[8] Cioci G, Mitchell E P, Gautier C, Wimmerová M, Sudakevitz D, Pérez S, Gilboa-Garber N, Imberty A (2003). FEBS Lett.

[9] Garber N, Guempel U, Belz A, Gilboa-Garber N, Doyle R J (1992). Biochim Biophys Acta.

[10] Diggle S P, Stacey R E, Dodd C, Cámara M, Williams P, Winzer K (2006). Environ Microbiol.

[11] Mitchell E, Houles C, Sudakevitz D, Wimmerová M, Gautier C, Pérez S, Wu M A, Gilboa-Garber N, Imberty A (2002). Nat Struct Mol Biol.

[12] Loris R, Tielker D, Jaeger K-E, Wyns L (2003). J Mol Biol.

[13] Tielker D, Hacker S, Loris R, Strathmann M, Wingender J, Wilhelm S, Rosenau F, Jaeger K-E (2005). Microbiology.

[14] Mammen M, Choi S K, Whitesides G M (1998). Angew Chem, Int Ed.

[15] Lee Y C, Lee R T (1995). Acc Chem Res.

[16] Lundquist J J, Toone E J (2002). Chem Rev.

[17] Renaudet O, Roy R (2013). Multivalent Scaffolds in Glycoscience. Chem Soc Rev.

[18] Roy R (1996). Curr Opin Struct Biol.

[19] Mortell K H, Gingras M, Kiessling L L (1994). J Am Chem Soc.

[20] Roy R, Laferrière C (1990). J Chem Soc, Chem Commun.

[21] Spaltenstein A, Whitesides G M (1991). J Am Chem Soc.

[22] Chabre Y M, Roy R (2013). Chem Soc Rev.

[23] Chabre Y M, Roy R (2010). Adv Carbohydr Chem Biochem.

[24] Turnbull W B, Stoddart J F (2002). J Biotechnol.

[25] Johansson E M V, Kadam R U, Rispoli G, Crusz S A, Bartels K-M, Diggle S P, Cámara M, Williams P, Jaeger K-E, Darbre T (2011). Med Chem Commun.

[26] Johansson E M V, Crusz S A, Kolomiets E, Buts L, Kadam R U, Cacciarini M, Bartels K-M, Diggle S P, Cámara M, Williams P (2008). Chem Biol.

[27] Consoli G M L, Granata G, Cafiso V, Stefani S, Geraci C (2011). Tetrahedron Lett.

[28] Kadam R U, Bergmann M, Hurley M, Garg D, Cacciarini M, Swiderska M A, Nativi C, Sattler M, Smyth A R, Williams P (2011). Angew Chem, Int Ed.

[29] Reymond J-L, Bergmann M, Darbre T (2013). Chem Soc Rev.

[30] Sleiman M, Varrot A, Raimundo J-M, Gingras M, Goekjian P G (2008). Chem Commun.

[31] Gingras M, Raimundo J-M, Chabre Y M (2006). Angew Chem, Int Ed.

[32] Gingras M, Pinchart A, Dallaire C (1998). Angew Chem, Int Ed.

[33] Bergamini G, Fermi A, Botta C, Giovanella U, Di Motta S, Negri F, Peresutti R, Gingras M, Ceroni P (2013). J Mater Chem C.

[34] Tucker J H R, Gingras M, Brand H, Lehn J-M (1997). J Chem Soc, Perkin Trans 2.

[35] Chabre Y M, Brisebois P P, Abbassi L, Kerr S C, Fahy J V, Marcotte I, Roy R (2011). J Org Chem.

[36] Gingras M, Chabre Y M, Roy M, Roy R (2013). Chem Soc Rev.

[37] Azev Y A, Dülcks T, Gabel D (2003). Tetrahedron Lett.

[38] Tropper F D, Andersson F O, Braun S, Roy R (1992). Synthesis.

[39] Lafont D, D’Attoma J, Gomez R, Goekjian P G (2011). Tetrahedron: Asymmetry.

[40] Lee B-Y, Park S R, Jeon H B, Kim K S (2006). Tetrahedron Lett.

[41] Sabin C, Mitchell E P, Pokorná M, Gautier C, Utille J-P, Wimmerová M, Imberty A (2006). FEBS Lett.

[42] Bernardi A, Jiménez-Barbero J, Casnati A, De Castro C, Darbre T, Fieschi F, Finne J, Funken H, Jaeger K-E, Lahmann M (2013). Chem Soc Rev.

[43] Soomro Z H, Cecioni S, Blanchard H, Praly J-P, Imberty A, Vidal S, Matthews S E (2011). Org Biomol Chem.

[44] Cecioni S, Oerthel V, Iehl J, Holler M, Goyard D, Praly J-P, Imberty A, Nierengarten J-F, Vidal S (2011). Chem – Eur J.

[45] Cecioni S, Faure S, Darbost U, Bonnamour I, Parrot-Lopez H, Roy O, Taillefumier C, Wimmerová M, Praly J-P, Imberty A (2011). Chem – Eur J.

[46] Cecioni S, Lalor R, Blanchard B, Praly J-P, Imberty A, Matthews S E, Vidal S (2009). Chem – Eur J.

[47] Otsuka I, Blanchard B, Borsali R, Imberty A, Kakuchi T (2010). ChemBioChem.

[48] Pertici F, Pieters R J (2012). Chem Commun.

[49] Reynolds M, Marradi M, Imberty A, Penadés S, Pérez S (2012). Chem – Eur J.

[50] Chabre Y M, Giguère D, Blanchard B, Rodrigue J, Rocheleau S, Neault M, Rauthu S, Papadopoulos A, Arnold A A, Imberty A (2011). Chem – Eur J.

