# Supplementary material for: Expeditive synthesis of trithiotriazine-cored glycoclusters and inhibition of Pseudomonas aeruginosa biofilm formation
Source: Beilstein J Org Chem. 2014 Aug 25;10:1981–90. doi: 10.3762/bjoc.10.206 (PMC4168900; doi:10.3762/bjoc.10.206)
Supplement: File 1 — Experimental procedures, characterization checklist and NMR, DLS and ITC data. [file Beilstein_J_Org_Chem-10-1981-s001.pdf]

Supporting Information
for
Expeditive synthesis of trithiotriazine-cored glycoclusters and inhibition of *Pseudomonas aeruginosa* biofilm formation

Meriem Smadhi[1,2], Sophie de Bentzmann*[3], Anne Imberty*[4], Marc Gingras[5], Raoudha Abderrahim[2] and Peter G. Goekjian*[1]

Address: [1]Laboratoire Chimie Organique 2 Glycochimie, Université de Lyon, ICBMS, UMR 5246 – CNRS, Université Claude Bernard Lyon 1, Bat. 308 –CPE Lyon, 43 Bd. du 11 Novembre 1918, 69622 Villeurbanne, France. Fax: +33-4-72448109; Tel: +33-4-72448183, [2]Université de Carthage, Faculté des sciences Bizerte, Tunisie, [3]Laboratoire d'Ingénierie des Systèmes Macromoléculaires, Institut de Biologie Structurale et Microbiologie, CNRS-Aix Marseille University, UMR7255, 31 Chemin Joseph Aiguier, 13402 Marseille Cedex 20, France, [4]Centre de Recherches sur les Macromolécules Végétales (CERMAV), UPR 5301 CNRS et Université Grenoble Alpes, BP53, 38041 Grenoble, France, and [5]Aix-Marseille Université, CNRS, CINaM UMR 7325, 163 Avenue de Luminy 13288 Marseille, France.

E-mail: Peter G. Goekjian* - goekjian@univ-lyon1.fr; Sophie de Bentzmann* - bentzman@imm.cnrs.fr; Anne Imberty* - imberty@cermav.cnrs.fr
*Corresponding author

Experimental procedures, characterization checklist and NMR, DLS and ITC data.

Materials and Methods	2
Experimental procedures	2
Characterization checklist	5
^{1}H spectrum of compound **1**	6
^{13}C spectrum of compound **1**	7
^{13}C DEPT spectrum of compound **1**	8
^{1}H-^{1}H 2D COSY spectrum of compound **1**	9
^{1}H-^{13}C 2D HSQC spectrum of compound **1**	10
^{1}H-^{13}C 2D HMBC spectrum of compound **1**	11
^{1}H spectrum of compound **2**	12
^{13}C spectrum of compound **2**	13
^{1}H spectrum of compound **9**	14
^{13}C spectrum of compound **9**	15
^{1}H-^{1}H 2D COSY spectrum of compound **9**	16
^{1}H spectrum of compound **10**	17
^{13}C spectrum of compound **10**	18
^{1}H-^{1}H 2D COSY spectrum of compound **10**	19
^{1}H-^{13}C 2D HMBC spectrum of compound **10**	20
^{1}H spectrum of compound **11**	21
^{1}H spectrum of compound **12**	22
^{13}C spectrum of compound **12**	23
^{13}C DEPT spectrum of compound **12**	24
^{1}H-^{1}H 2D COSY spectrum of compound **12**	25
^{1}H-^{13}C 2D HSQC spectrum of compound **12**	26
^{1}H-^{13}C 2D HMBC spectrum of compound **12**	27
^{1}H spectrum of compound **13**	28
^{13}C spectrum of compound **13**	29
^{13}C DEPT spectrum of compound **13**	30
^{1}H-^{1}H 2D COSY spectrum of compound **13**	31
^{1}H-^{13}C 2D HSQC spectrum of compound **13**	32
^{1}H-^{13}C 2D HMBC spectrum of compound **13**	33
^{1}H spectrum of compound **14**	34
^{13}C spectrum of compound **14**	35
^{13}C DEPT spectrum of compound **14**	36
^{1}H-^{1}H 2D COSY spectrum of compound **14**	37
^{1}H-^{13}C 2D HSQC spectrum of compound **14**	38
^{1}H-^{13}C 2D HMBC spectrum of compound **14**	39
^{1}H spectrum of compound **15**	40
^{13}C spectrum of compound **15**	41
^{13}C DEPT spectrum of compound **15**	42
^{1}H-^{1}H 2D COSY spectrum of compound **15**	43
^{1}H-^{13}C 2D HSQC spectrum of compound **15**	44
^{1}H-^{13}C 2D HMBC spectrum of compound **15**	45
^{1}H spectrum of compound **16**	46
^{13}C spectrum of compound **16**	47
^{13}C DEPT spectrum of compound **16**	48
^{1}H-^{1}H 2D COSY spectrum of compound **16**	49
^{1}H-^{13}C 2D HSQC spectrum of compound **16**	50
^{1}H-^{13}C 2D HMBC spectrum of compound **16**	51
^{1}H spectrum of compound **17**	52
^{13}C spectrum of compound **17**	53
^{1}H-^{1}H 2D COSY spectrum of compound **17**	54
^{1}H-^{13}C 2D HSQC spectrum of compound **17**	55
^{1}H-^{13}C 2D HMBC spectrum of compound **17**	56
DLS spectrum of LecA + **1**	57
DLS spectrum of LecA + **13**	58
DLS spectrum of LecA + **17**	58
DLS spectrum of LecA + **16**	58
ITC titration curve of Lec A + **13**	59
ITC titration curve of Lec A + **16**	60
ITC titration curve of Lec A + **17**	61
ITC titration curve of Lec A + **1** (run 2)	62
ITC titration curve of LecB + **14**	63
Biofilm quantification	64

Materials and methods:

All reagents were commercial and used without further purification. Solvents were distilled from CaH_2. Reactions under microwave activation were performed using a Biotage Initiator system. NMR spectra were recorded at 293 K, unless otherwise stated, using a 300 or 400 or 500 MHz spectrometer. Shifts are referenced relative to deuterated solvent residual peaks. Complete signal assignments from 1D and 2D NMR spectroscopy were based on COSY, HSQC, and HMBC correlations. High-resolution (HR-ESI- QToF) mass spectra were recorded using a Bruker MicroToF-Q II XL spectrometer. Thin-layer chromatography (TLC) was carried out on aluminum sheets coated with silica gel 60 F254 (Merck). TLC plates were inspected by UV light (λ = 254 nm) and developed by treatment with a mixture of 10% H_2SO_4 in EtOH/H_2O (1:1 v/v) followed by heating. Optical rotation was mesured by using a Perkin-Elemer polarimeter and values are given in 10^{-1} deg.cm^2.g^{-1}. IR spectra were recorded on a Shimadzu FTIR-8400S IR spectrophotometer and n are expressed in cm^{-1}. NMR spectra were recorded on a Bruker DRX (^{1}H at 300 or 400 MHz, ^{13}C at 75 or 100 MHz) or on a Bruker AV500 spectrometer (^{1}H at 500 MHz, ^{13}C at 125 MHz). HRMS were recorded in electrospray ionization mode on a micro q-tof Micromass in strument (3000 V) with an internal lock mass (H_3PO_4) and an external lock mass (Leu-enkephaline).

Experimental procedures

Isothermal titration microcalorimetry (ITC):

Recombinant lyophilized lecA and lecB were dissolved in buffer (100 mM Tris-HCl pH 7.5, 6 µM $CaCl_2$,) and degassed. The same procedure was applied for lecB with a different buffer (20mM Tris-HCl pH 7.54 100mM NaCl 100µM $CaCl_2$). Protein concentration varied between 50 and 120 µM depending on the ligand affinity. Carbohydrate ligands were dissolved directly into the same buffer, degassed, and placed in the injection syringe. ITC was performed using a VP-ITC MicroCalorimeter from MicroCal Incorporated. lecA was placed into the 1.4478 mL sample cell, at 25°C. Titration was performed with 10 µL injections of carbohydrate ligands every 300 s. Data were fitted using MicroCal Origin 7 software according to standard procedures. Fitted data yielded the stoichiometry (n), the association constant (K_a), and the enthalpy of binding (ΔH). Other thermodynamic parameters (i.e., changes in free energy ΔG and entropy ΔS) were calculated from the equation $\Delta G = \Delta H - T\Delta S = -RTlnK_d$ in which T is the absolute temperature and R = 8.314 $Jmol^{-1}K^{-1}$. Two or three independent titrations were performed for each ligand tested.

Assay protocol for *Pseudomonas aeruginosa* biofilm formation:

P. aeruginosa PAO1 ON preculture grown in LB was inoculated into fresh culture medium (LB) in 20-well plates at an initial OD_{600nm} of 0.1 in 24-well microplates that were incubated alone or in the presence of galactose (1), fucose (14), and glucose (13, control)-substituted trivalent clusters at 30°C for 24 hrs and

further incubated with 1 % Crystal Violet for 10 min and washed twice. Staining was extracted by treatment with 40 % ethanol and values of OD_{570nm} were measured. All quantification assays were made at least in triplicate.

Synthesis

2,4,6-tris(prop-2-ynylthio)-1,3,5-triazine (2)

1,3,5-triazine-2,4,6 trithiol trisodium salt (2 mmol, 1 eq), K_2CO_3 (1 eq, 270 mg), and propargyl bromide (0.56 mL, 3.3 eq) in acetonitrile (2 mL) were stirred for 15 min at 0°C and at room temperature for 4h. The residue was purified by silica gel flash chromatography (PE/EtOAc 4/1) (Yield = 56%). M.p 91-92°C. TLC (SiO_2 ; PE :EtOAc/ 3:1) R_f = 0.6. ^{1}H NMR (300 MHz, $CDCl_3$) δ = 3.90 ppm (d, 6H, *J* = 2.5 Hz), 2.22 (t, 3H, *J* = 2.5 Hz). ^{13}C NMR (300 MHz, $CDCl_3$) δ = 178.6 ppm (C-a), 78.7 (C-c), 71.6 (C-d), 19.2 (C-b). HRMS (ESI) : *m/z* calcd for $C_{12}H_{10}N_3S_3$ [M + H]+ : 292.0038 ; found : 292.0031.

3,4,6-tri-*O*-t-butyldimethylsilyl-D-galactopyranosyl azide (5)

A solution of oxone (1.257g, 2.04mmol) in H_2O (5.1mL) was added dropwise over 15 min to a vigorously stirred, cooled (ice bath) biphasic solution of tri-*O*-t-butyldimethylsilyl-D-galactal (500mg, 1.02mmol), acetone (0.5mL), Bu_4NHSO_4 (0.367mmol, 0.148g) ,CH_2Cl_2 (5mL) and sat aq $NaHCO_3$ (7.22mL). The two-phase mixture was vigorously stirred at r.t for 3 h. The organic phase was separated and the aqueous phase was extracted with CH_2Cl_2 (2 x 30 mL). The combined organic phases were washed once with water (10mL), dried on Na_2SO_3 (sodium sulfite) and concentrated to 6 ml. To this solution was added successively a sat aq $NaHCO_3$ (6 mL), sodium azide (265.1mg, 4.08mmol) and Bu_4NHSO_4 again (346.5mg, 1.02mmol). The mixture was vigorously stirred for 4h, then extracted with ethyl acetate (60 mL) and washed once with sat aq NaHCO3 (10mL). After drying and concentration, the crude product was purified by column chromatography (4:1 ethyl acetate- petroleum ether). (Yield=70%). TLC (SiO_2; PE :CH_2Cl_2/ 1:4) Rf=0.24 ^{1}HNMR (300MHz, $CDCl_3$) δ=4.37 ppm (dd, 1H, J=8.2Hz, J=10.9, H-1), 3.95-4.04 (m, 1H), 3.65-3.76 (m, 3H), 3.38-3.61 (m, 3H), 0.96-0.71 (m, 27H), 0.21-0.04 (m, 18H). ESI-MS (positive mode) m/z : 570.2 $[M+Na]^+$, 542.3 $[M-N_2+Na]^+$. Further characterization was not performed due to migration of the silyl groups.

Acetyl-protected tris-β-D-Gal triazine glycocluster, 9

A solution of compound **2** (21.1 mg, 0.920 mmol, 1 eq.), 2,3,4,6-tetra-*O*-acetyl-β-D-glucopyranosyl azide (113.4 mg, 0.303 mmol, 3.3 eq.), CuI (0.02 mmol, 5.2 mg, 0.3 eq.) and DIPEA (1.38 mmol, 0.240 mL, 15 eq.) in DMF (2mL) was heated under microwave irradiation for 15 minutes at 110°C. The reaction mixture was diluted with CH_2Cl_2 (5 mL) and H_2O (5 mL). The organic layer was separated, dried over $MgSO_4$, and concentrated. The residue was purified by silica gel flash chromatography (PE/EtOAc , 1:3). Yield = 73%. $[\alpha]_D$ = +1.1 (c = 0.5; CH_2Cl_2). ^{1}H NMR (300 MHz, $CDCl_3$) δ = 7.87 ppm (s, 3H, H-d), 5.81 (d, 3H, *J* = 9.3 Hz, H-1), 5.54 (br s, 3H, H-4), 5.53 (dd, 3H, J_1 = 10.2 Hz, J_2 = 9.3 Hz, H-2), 5.23 (dd, 3H, J_1 = 10.2 Hz, J_2 = 3.3 Hz, H-3), 4.50 (d, 3H, *J* = 14.8 Hz, H-b), 4.41 (d, 3H, *J* = 14.8 Hz, H-b'), 4.26-

4.11 (m, 9H, H-5, H-6), 2.21 (s, 9H, CH_3), 2.04 (s, 9H, CH_3), 2.01 (s, 9H, CH_3), 1.84 (s, 9H, CH_3). ^{13}C NMR (75 MHz, $CDCl_3$) δ = 179.2 ppm (C-a) 170.7, 170.4, 170.2, 169.3 (CO esters) 144.6 (C-c) 121.7 (C-d) 86.6 (C-1); 74.4 ; 71.2 ; 68.3 ; 67.2 ; 61.5 ; 25.5 (C-b) 21.1, 21.1, 20.9, 20.6 (CH_3). HRMS (ESI): *m/z* calcd for $C_{54}H_{67}N_{12}NaO_{27}S_3$ [M + H + 2Na]$^{++}$: 717.1666 ; found : 717.1644.

Acetyl-protected tris-β-D-Glc triazine glycocluster, 10

A solution of compound **2** (20 mg, 0.069 mmol, 1 eq.), 2,3,4,6-tetra-*O*-acetyl-β-D-galactopyranosyl azide (84.1 mg, 0.226 mmol, 3.3 eq.), CuI (0.02 mmol, 4 mg, 0.3 eq.) and DIPEA (1.03 mmol, 0.17 ml, 15 eq.) in DMF (1ml) was heated under microwave irradiation for 15 minutes at 110°C. The reaction mixture was diluted with CH_2Cl_2 (5mL) and H_2O (5mL). The organic layer was separated, dried over $MgSO_4$, and concentrated. The residue was purified by silica gel flash chromatography (PE/EtOAc , 1:3). Yield = 92%. TLC (SiO_2; PE:EtOAc/ 1:3) R_f = 0.40. ^{1}HNMR (300 MHz, $CDCl_3$) δ = 8.03ppm (s, 3H, H-d) 5.92 (d, 3H, *J* = 9.0 Hz, H-1) 5.93 (d, 3H, *J* = 9.0 Hz, H-1) 5.49 (dd, 3H, *J* = 9.0, 9.3 Hz, H-2) 5.41 (dd, 3H, *J* = 9.3, 9.0 Hz H-3) 5.33 (dd, 3H, *J* = 9.0, 9.6 Hz, H-4) 4.49 (s, 6H, H-b) 4.29 (dd, 3H, *J* = 4.8 Hz, *J* = 12.8 Hz, H-6’) 4.14 (dd, 3H, *J* = 2.1 Hz, *J* = 12.6 Hz, H-6’) 4.04 (ddd, 3H, , *J* = 2.1 Hz, *J* = 4.8 Hz, *J* = 9.6 Hz H-5) 2.08 (s, 9H, CH_3) 2.04 (s, 9H, CH_3) 2.03 (s, 9H, CH_3), 1.80 (s, 9H, CH_3). ^{13}CNMR (75 MHz, $CDCl_3$) δ = 179.1ppm (C-a) 170.9, 170.3, 169.9, 169.2 (CO esters) 145.0 (C-c) 121.8 (C-d) 86.1(C-1) ; 75.5 ; 73.1 ; 70.6 ; 68.2 ; 63.1 ; 24.6 (C-b) 21.1, 21.0, 20.5 (CH_3). HRMS (ESI) : *m/z* calcd for $C_{54}H_{66}N_{12}Na_2O_{27}S_3$ [M + 2Na]$^{++}$: 728.1561 ; found : 728.1553.

Silyl-protected tris-β-D-Gal triazine glycocluster, 11

To compound **2** (15.9 mg, 0.054 mmol, 1 eq.), 3,4,6-tri-*O*-t-butyldimethylsilyl-D-galactopyranosyl azide (**5**) (98.6 mg, 0.17 mmol, 3.3 eq.) in CH_2Cl_2 (0.5mL) and H_2O (0.5mL) were added $CuSO_4.5H_2O$ (15%, 2 mg, 0.008 mmol) and sodium ascorbate (45%, 4.8 mg, 0.024 mmol). The resulting solution was stirred for 24h at room temperature. The reaction mixture was diluted with CH_2Cl_2 (5 mL) and H_2O (5 mL). The organic layer was separated, dried over $MgSO_4$, and concentrated. The residue was purified by column chromatography (PE/EtOAc, 4:1) to give the silyl-protected glycocluster **11**. (Yield = 87%). TLC (SiO_2; PE:EtOAc/ 7:3) R_f = 0.77 ^{1}HNMR (300 MHz, $CDCl_3$) δ = 7.64ppm (s, 3H, H-d) 5.48 (d, *J* = 7 Hz, 3H) 4.30-4.47(m, 6H) 4.08 (d, *J* = 1.7 Hz, 3H) 3.62-3.72 (m, 12H) 0.87-0.97 (m, 81H) 0.01-0.17 (m, 54H). ESI-MS (positive mode) m/z :1935.6 [M+H]$^+$, 1958.6 [M+Na]$^+$. Further characterization was not performed due to partial migration of the silyl groups.

Benzyl-protected tris-β-D-Glc triazine glycocluster, 12

To compound **2** (19.2 mg, 0.0659 mmol, 1 eq.), 3,4,6-tri-O-benzyl-β-D-glucopyranosyl azide (103.5 mg, 0.217 mmol, 3.3eq) in CH_2Cl_2 (0.5mL) and H_2O (0.5mL) were added $CuSO_4.5H_2O$ (15%, 2.3 mg, 0.0098 mmol) and sodium ascorbate (45%, 3.6 mg, 0.018 mmol). The resulting solution was stirred for 24h at room temperature. The reaction mixture was diluted with CH_2Cl_2 (5 mL) and H_2O (5 mL). The organic layer was separated, dried over $MgSO_4$, and concentrated. The residue was purified by column chromatography (PE/EtOAc, 1:3) to give the nonabenzyl-protected glucocluster **10**. (Yield = 92%). TLC

(SiO_2; PE:EtOAc/ 1:3) R_f = 0.67. ^{1}H NMR (300 MHz, $CDCl_3$) δ = 7.78 ppm (s, 3H, H-d), 7.37-7.10 (m, 45H), 5.43 (d, 3H, *J* = 9.1 Hz, H-1), 4.99 (d, 3H, *J* = 11.3 Hz), 4.86 (d, 3H, *J* = 11.3 Hz), 4.85 (d, 3H, *J* = 10.8 Hz), 4.52 (d, 3H, *J* = 10.8 Hz), 4.53 (d, 3H, J_1 = 12.1 Hz), 4.45 (d, 3H, J_1 = 12.1 Hz), 4.13-4.27 (m, 9H, H-2, H-b), 3.80-3.59 (m, 15H, H-3, H-4, H-5, H-6). ^{13}C NMR (75 MHz, $CDCl_3$) δ = 179.1 ppm (C-a), 143.7 (C-c), 138.9, 138.3, 138.2, 128.8, 128.4, 128.3, 128.3, 128.2, 128.1, 128.0 (C-Bn), 122.9 (C-d), 88.6 (C-1) 85.5, 78.3, 77.2 (C-3, C-4, C-5), 75.9, 75.5, 73.9, (C-Bn), 73.6 (C-2), 68.5 (C-6), 25.3 (C-b). ESI-MS (positive mode) m/z : 1717.6 $[M+H]^+$, 1739.6 $[M+Na]^+$.

2,4,6-tris(1-(6-deoxy-6-*C*-α-D-mannopyranosyl)triazol-5-ylthio)-1,3,5-triazine (15)

Compound **2** (21.7 mg, 0.074 mmol, 1 eq.), methyl 6-azido-6-deoxy-α-D-mannopyranoside [Madzen, *Synthesis* 2002, *12*, 1721-1727.] (65.2 mg, 0.297 mmol, 4 eq.), CuI (0.022 mmol, 4.2 mg, 0.3 eq.) and DIPEA (1.11 mmol, 0.20 mL, 15 eq.) in DMF (1 mL) was heated under microwave irradiation for 15 minutes at 110°C. The reaction mixture was concentrated *in vacuo* and the residue was purified by C18 chromatography (Combiflash, Grace Reveleris C18 RP 4g Cartridge, H_2O/MeOH gradient). Yield = 47%. TLC (C18 ; MeOH :H_2O/ 1 :1) R_f = 0.5. $[\alpha]_D$ = +1.3 (c = 0.25; H_2O). ^{1}H NMR (500MHz, DMSO-d_6) δ = 8.01 ppm (s, 3H, H-d), 5.17 (d, 3H, *J* = 5.8 Hz, OH-4), 4.87 (d, 3H, *J* = 4.4 Hz, OH-2), 4.75 (d, 3H, *J* = 6.0 Hz, OH-3), 4.72 (dd, 3H, *J* = 14.2 Hz, *J* = 1.9 Hz, H-6), 4.51 (d, 3H, *J* = 14.5 Hz, H-b), 4.48 (d, 3H, *J* = 14.5 Hz, H-b') 4.42 (d, 3H, *J* = 1.2 Hz, H-1), 4.36 (dd, 3H, *J* = 14.2 Hz, *J* = 9.3 Hz, H-6'), 3.57-3.54 (m, 6H, H-2, H-5), 3.37 (ddd, 3H, *J* = 9.2 Hz, *J* = 6.0 Hz, *J* = 3.3 Hz, H-3), 3.43 (ddd, 3H, *J* = 9.5 Hz, *J* = 9.2 Hz, *J* = 5.9 Hz, H-4), 2.82 (s, 9H, OCH_3). ^{13}C NMR (125 MHz, DMSO-d_6) δ = 178.4 ppm (C-a), 142.3 (C-c), 124.4 (C-d), 101.0 (C-1), 71.7 (C-5), 70.6 (C-3), 70.0 (C-2), 68.0 (C-4), 53.6 (CH_3), 51.0 (C-6), 24.7 (C-b). HRMS (ESI) : *m/z* calcd for $C_{33}H_{50}N_{12}O_{15}S_3$ $[M + 2H]^{++}$: 475.1329 ; found : 475.1335.

Characterization checklist

Compound number	New	Known	Melting point range	IR	UV-Vis	1H NMR	13C NMR	DEPT	COSY	HSQC	HMBC	MS	HRMS	Optical rotation/ORD/CD	Enantiomeric/Diastereomeric ratio	X-ray [ORTEP and CIF in SI*]	<>	NMR spectrum in SI*
compound 2		X				X	X						X					X
compound 1	X			X		X	X	X	X	X	X		X	X				X
compound 14	X					X	X	X	X	X	X		X	X				X
compound 15	X					X	X	X	X	X	X		X	X				X
compound 13	X					X	X	X	X	X	X		X	X				X
compound 16	X					X	X	X	X	X	X		X	X				X
compound 17	X					X	X		X	X	X		X	X				X
compound 12	X					X	X	X	X	X	X		X	X				X
compound 11	X					X						X						X
compound 9	X					X	X		X				X	X				X
compound 10	X					X	X		X		X		X					X

Compound **11**: Further characterization was not performed due to partial migration of the silyl groups.

exp2 DMSO 500MHz

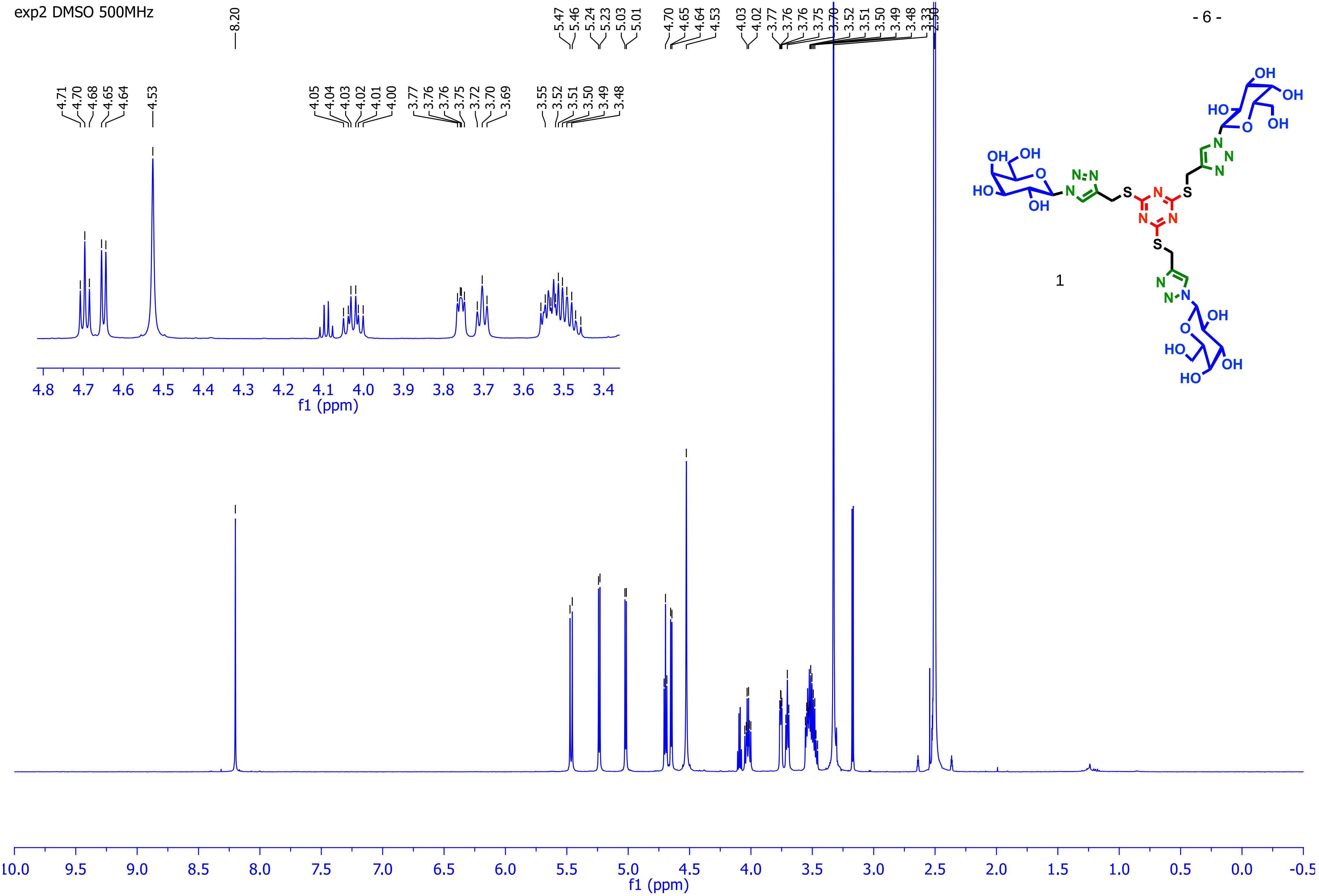

expl2
13C_dept135sp DMSO /opt/topspin lco2_pg 1

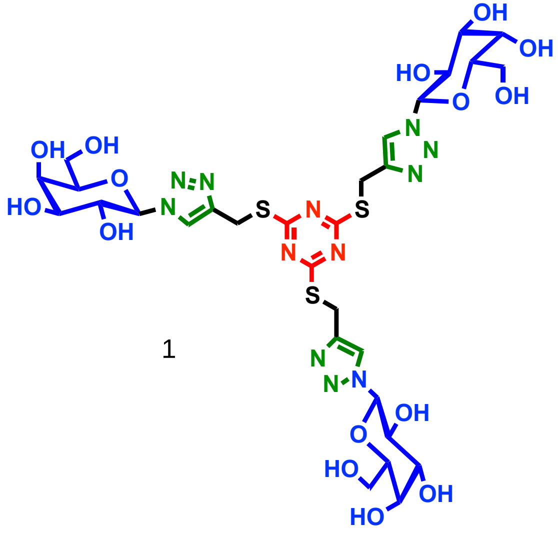

1

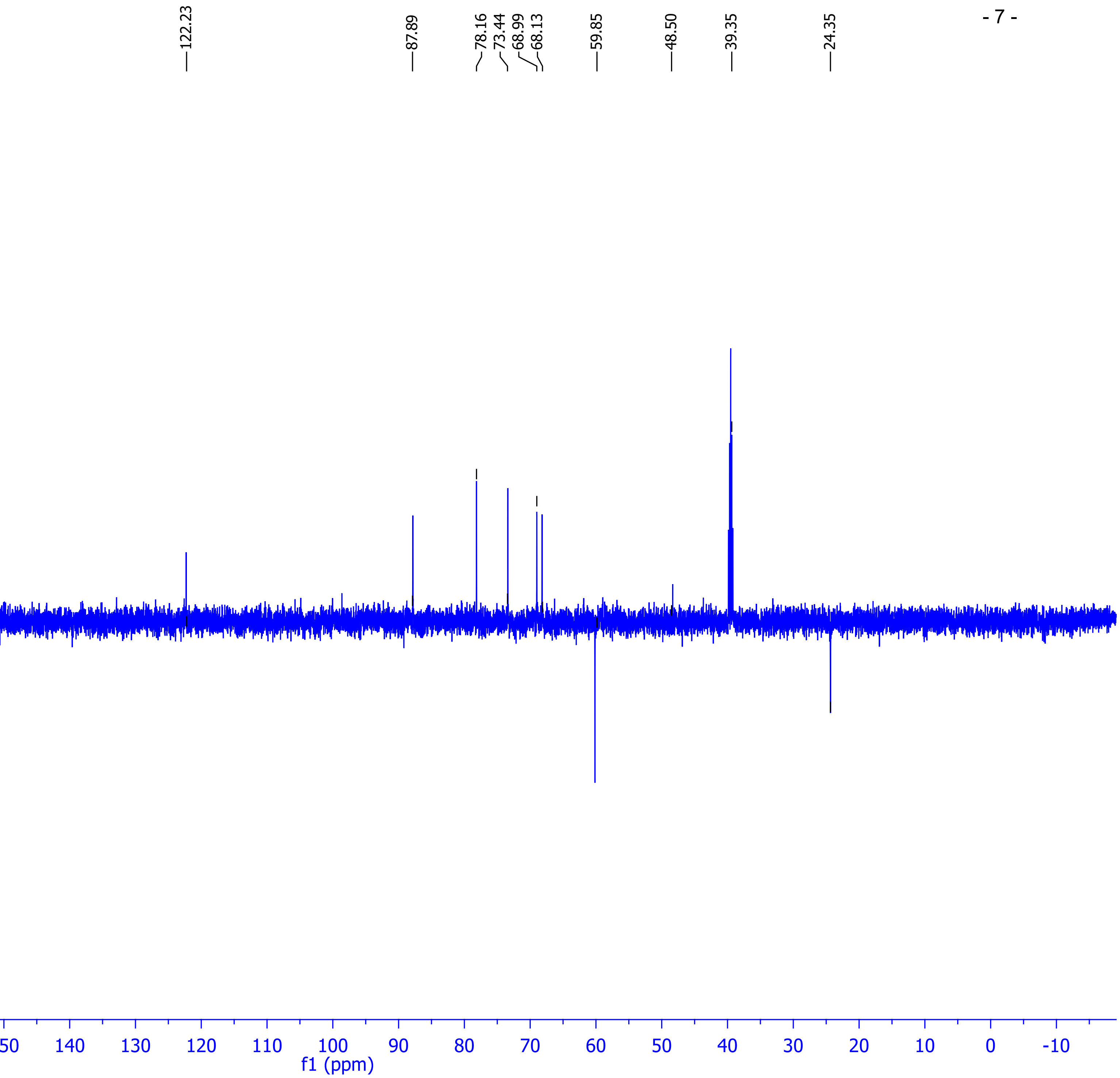

expl2 DMSO

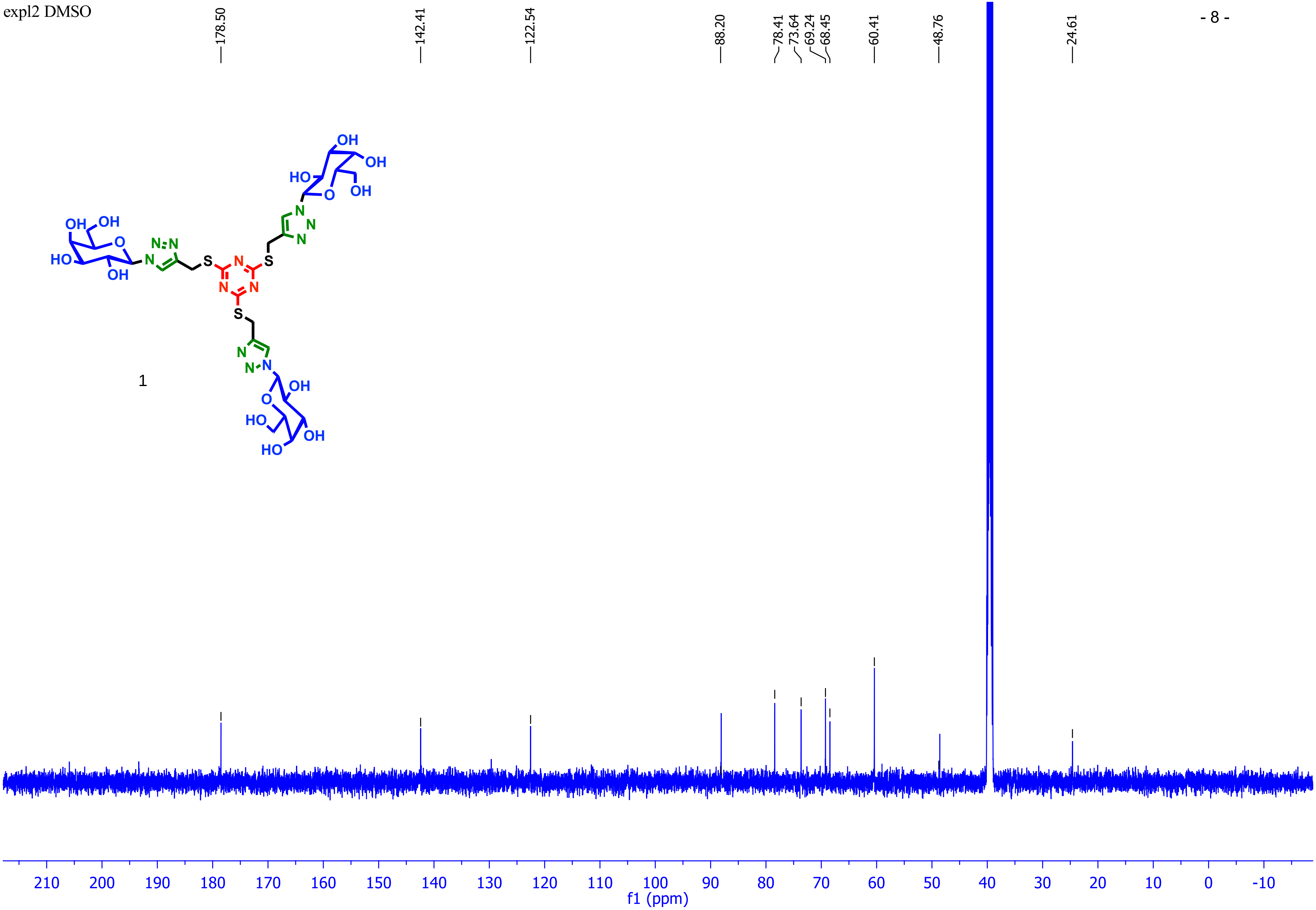
178.50
142.41
122.54
88.20
78.41
73.64
69.24
68.45
60.41
48.76
24.61
1
210 200 190 180 170 160 150 140 130 120 110 100 90 80 70 60 50 40 30 20 10 0 -10
f1 (ppm)

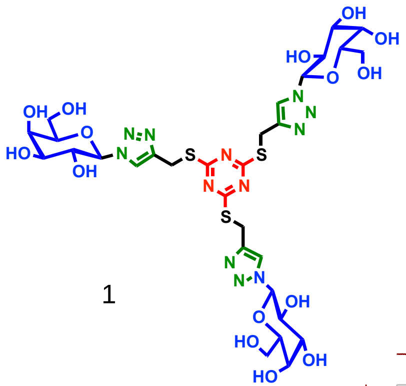

1

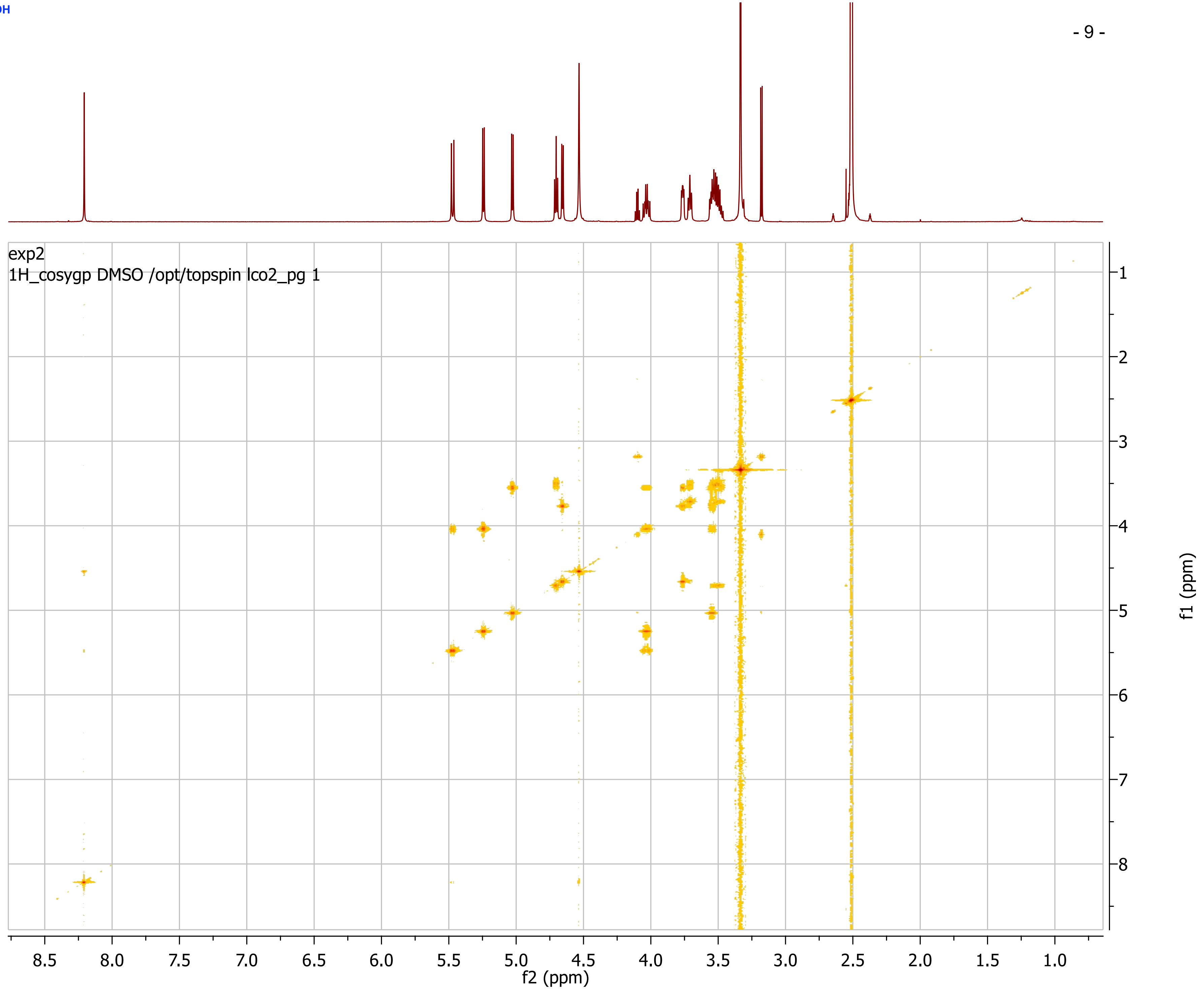

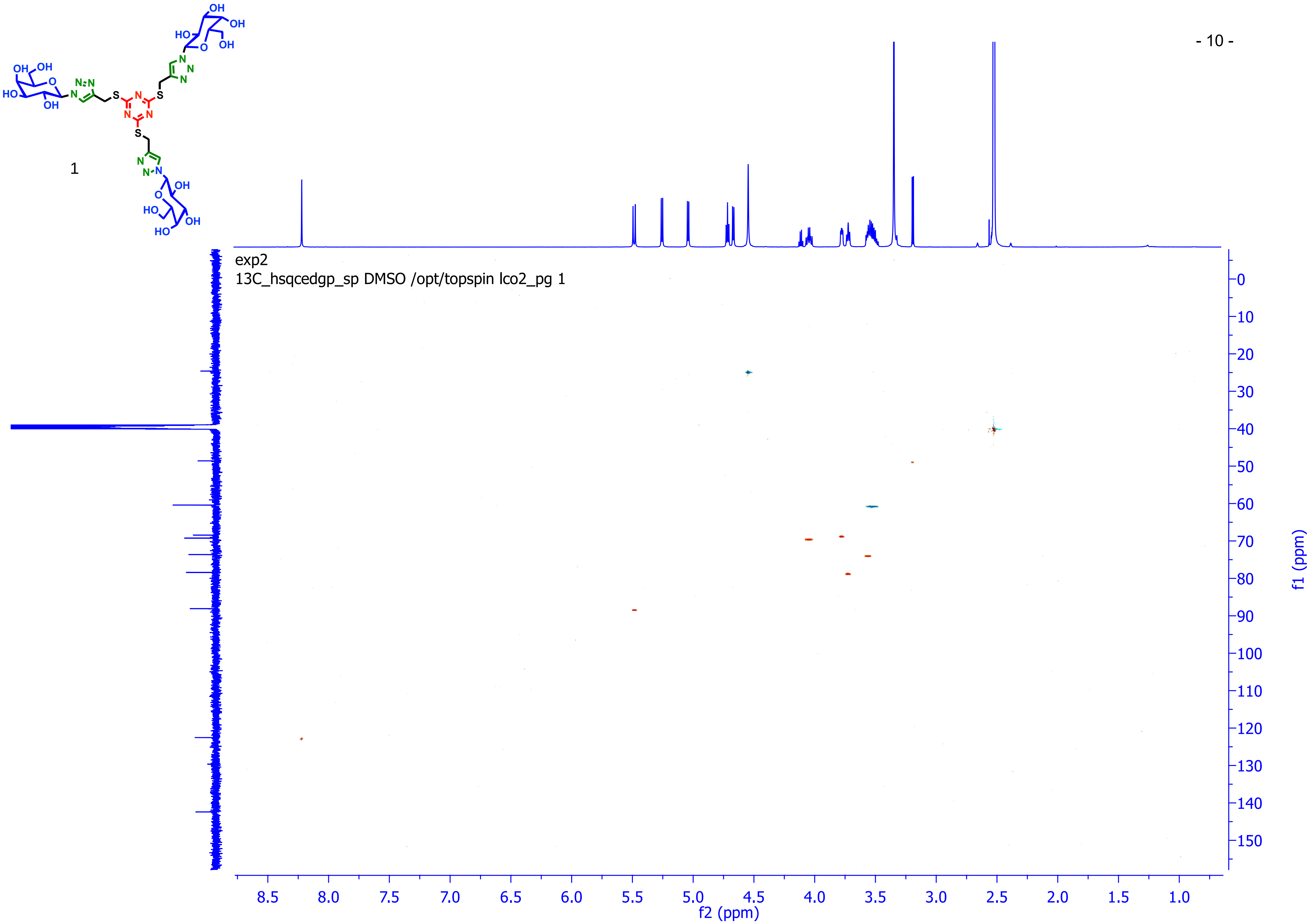
exp2
13C_hsqcedgp_sp DMSO /opt/topspin lco2_pg 1
f2 (ppm)
f1 (ppm)

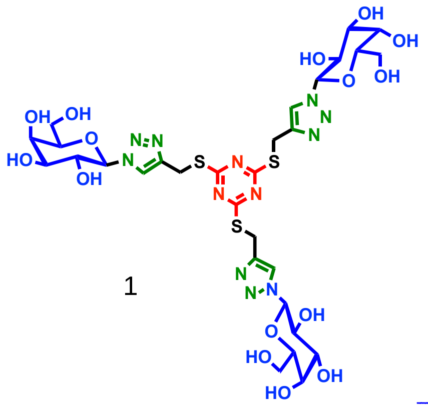

HO
OH
OH
N
N
S
1

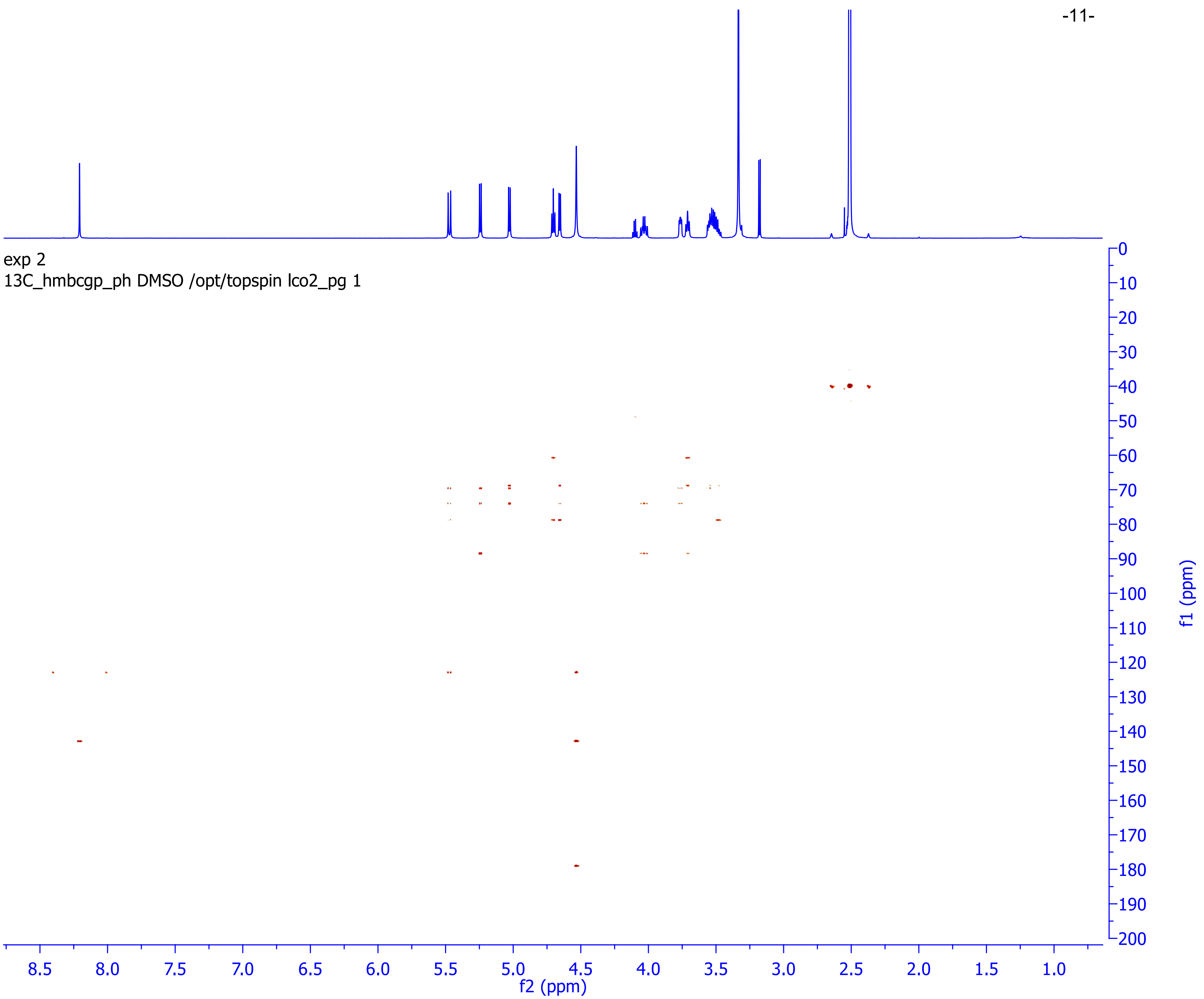

exp 2
13C_hmbcgp_ph DMSO /opt/topspin lco2_pg 1
f2 (ppm)
f1 (ppm)

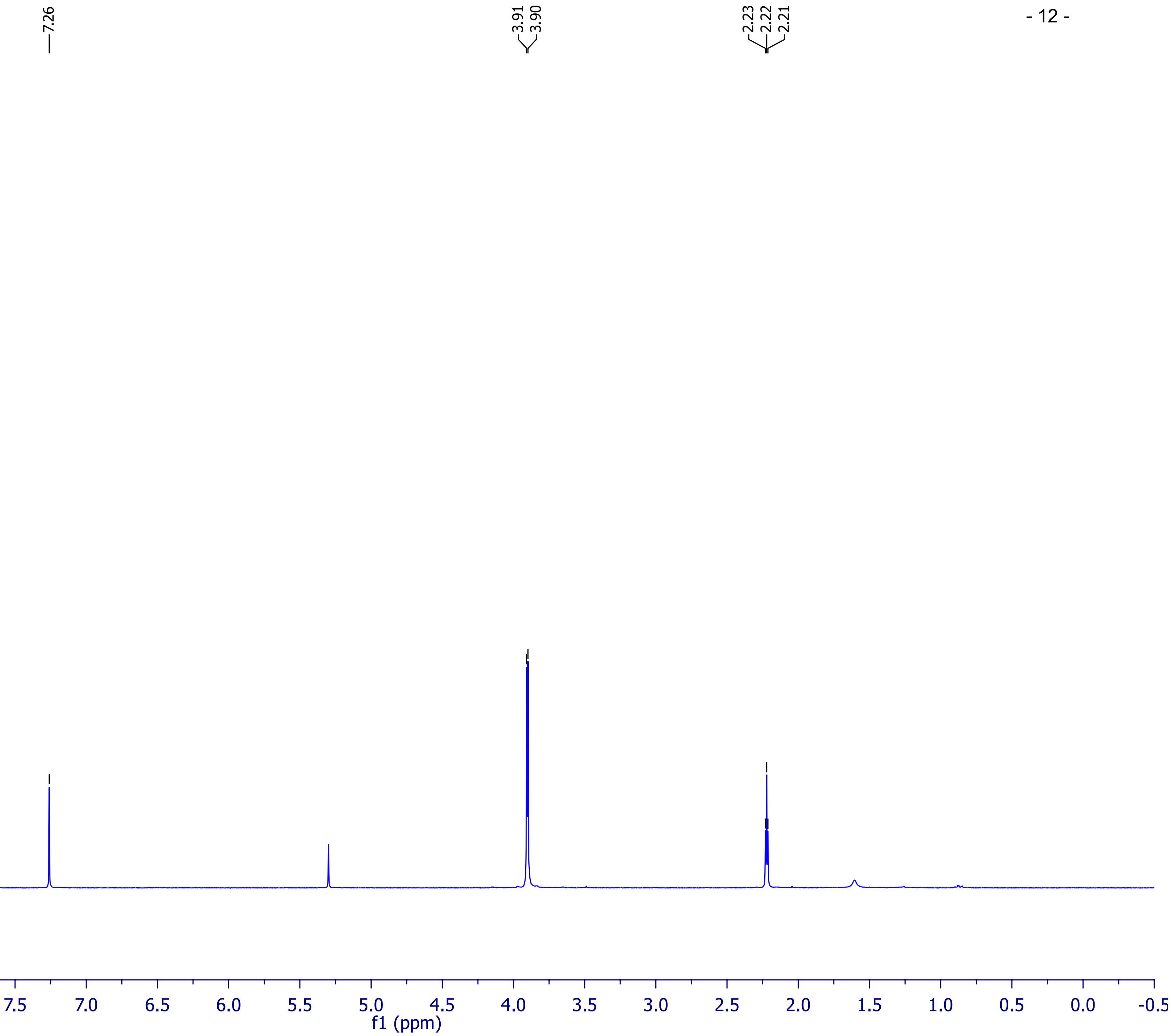
exp1 CDCl3 300MHz
7.26
3.91
3.90
2.23
2.22
2.21
10.0
9.5
9.0
8.5
8.0
7.5
7.0
6.5
6.0
5.5
5.0
4.5
4.0
3.5
3.0
2.5
2.0
1.5
1.0
0.5
0.0
-0.5
f1 (ppm)

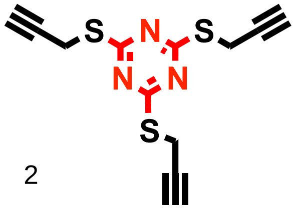
S
N
S
N
N
S

2

CDCl3 exp 1 300 MHz

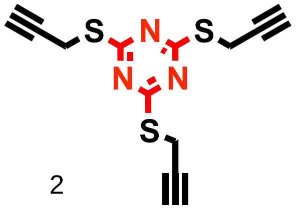

2

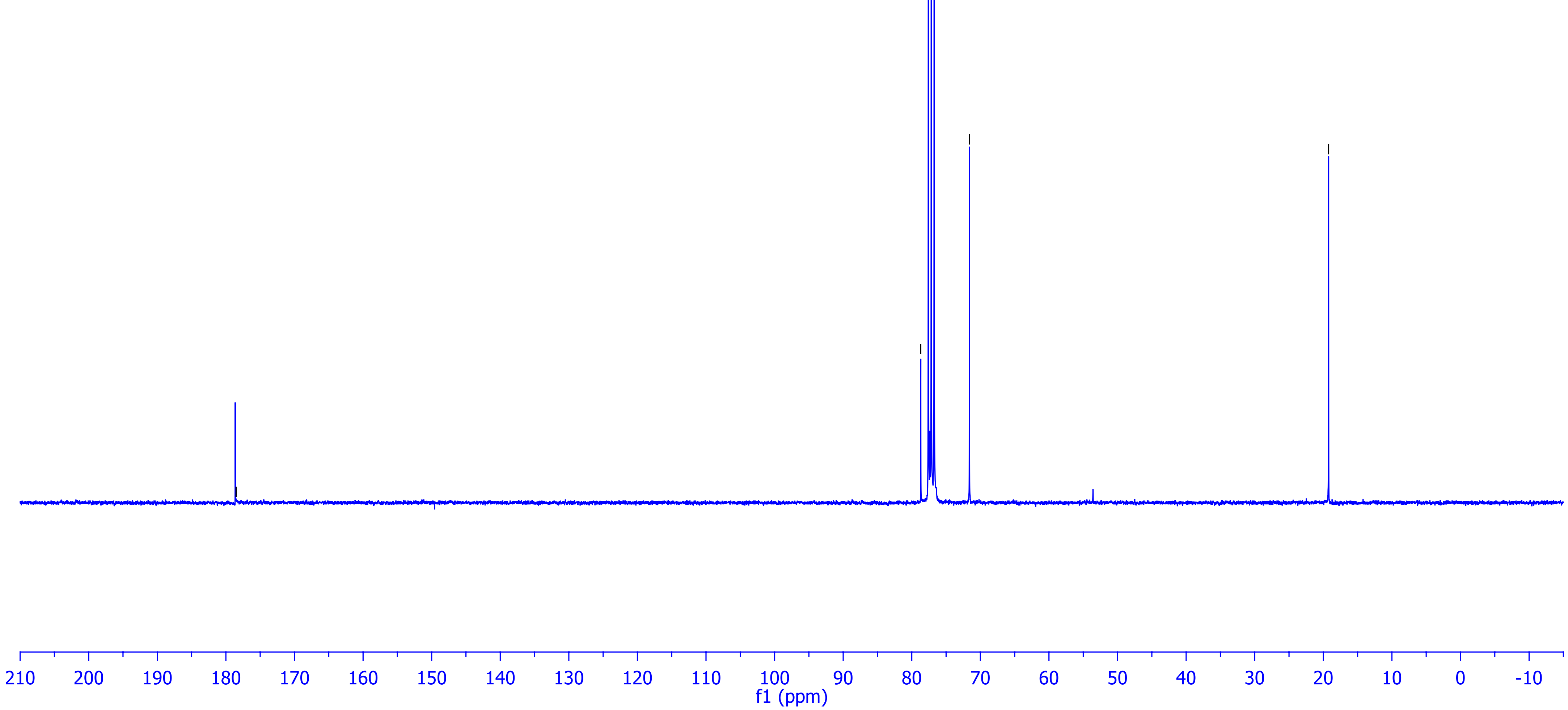

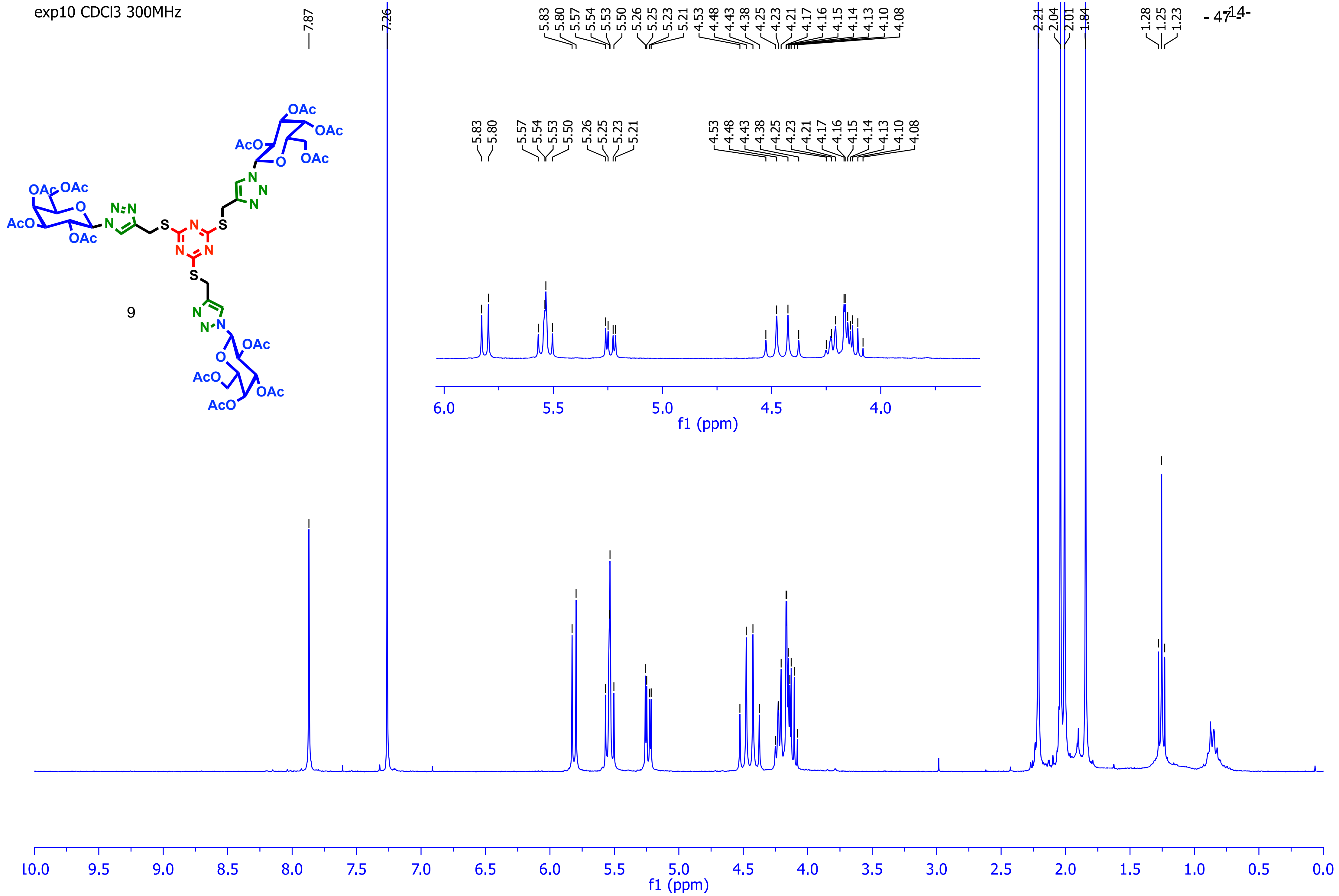
exp10 CDCl3 300MHz
7.87
7.26
5.83 5.80 5.57 5.54 5.53 5.50 5.26 5.25 5.23 5.21 4.53 4.48 4.43 4.38 4.25 4.23 4.21 4.17 4.16 4.15 4.14 4.13 4.10 4.08
2.21 2.04 2.01 1.84
1.28 1.25 1.23
OAc
AcO
O
N
S
9
f1 (ppm)
10.0 9.5 9.0 8.5 8.0 7.5 7.0 6.5 6.0 5.5 5.0 4.5 4.0 3.5 3.0 2.5 2.0 1.5 1.0 0.5 0.0
6.0 5.5 5.0 4.5 4.0

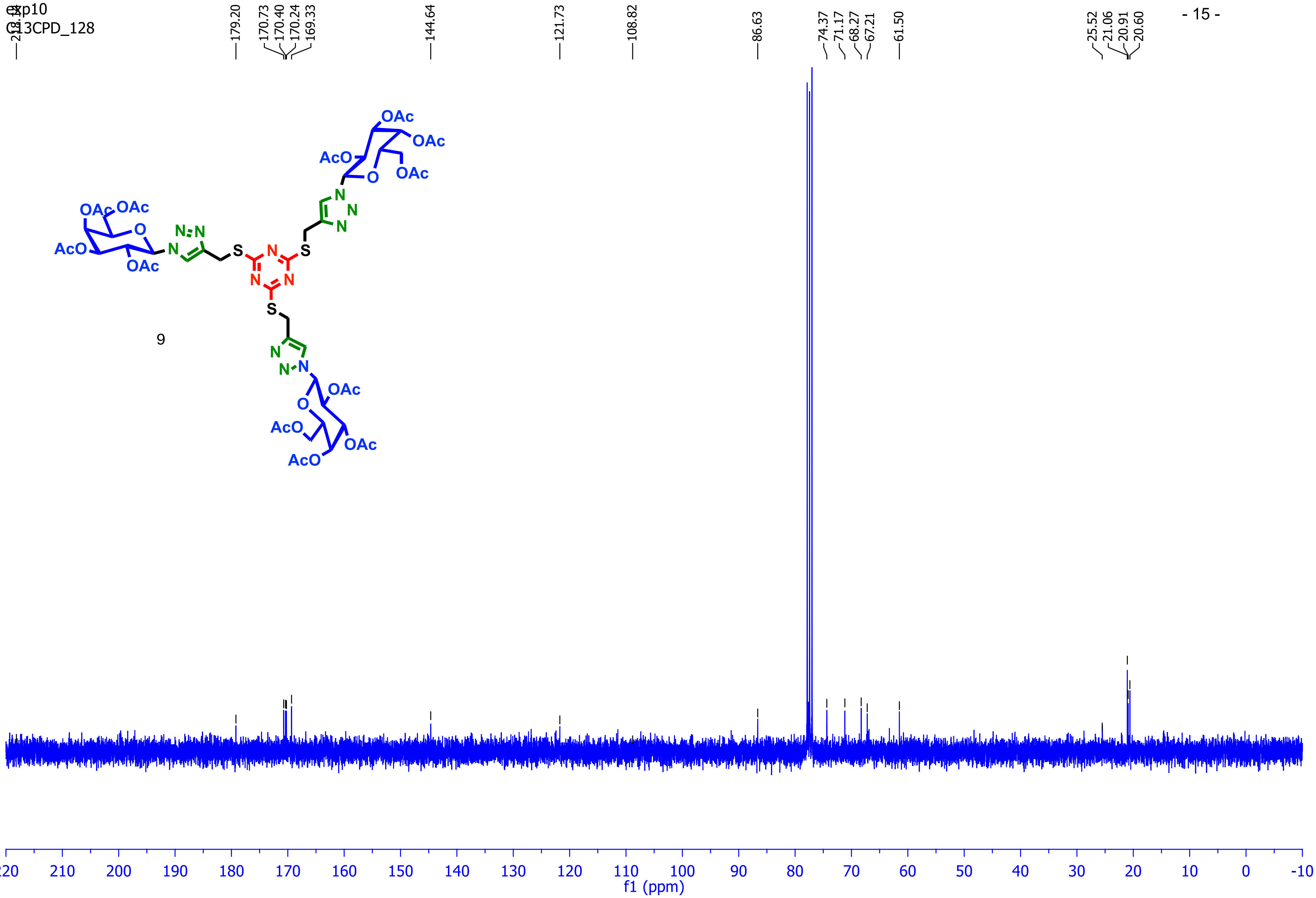
exp10
C13CPD_128
179.20
170.73
170.40
170.24
169.33
144.64
121.73
108.82
86.63
74.37
71.17
68.27
67.21
61.50
25.52
21.06
20.91
20.60
OAc
AcO
O
N
S
220 210 200 190 180 170 160 150 140 130 120 110 100 90 80 70 60 50 40 30 20 10 0 -10
f1 (ppm)

9

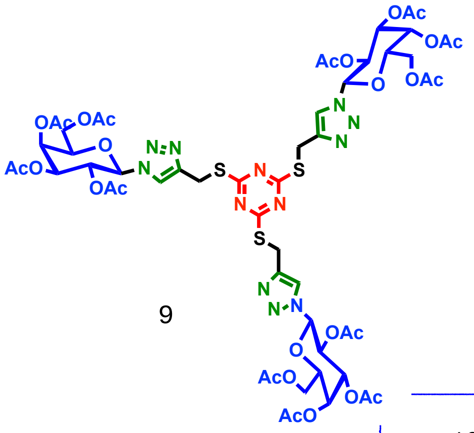
OAc
OAc
AcO
O
OAc
N
N
N
OAc OAc
AcO
O
OAc
N
N
N
S
S
N
N
N
S
N
N
N
OAc
O
AcO
AcO
OAc

9

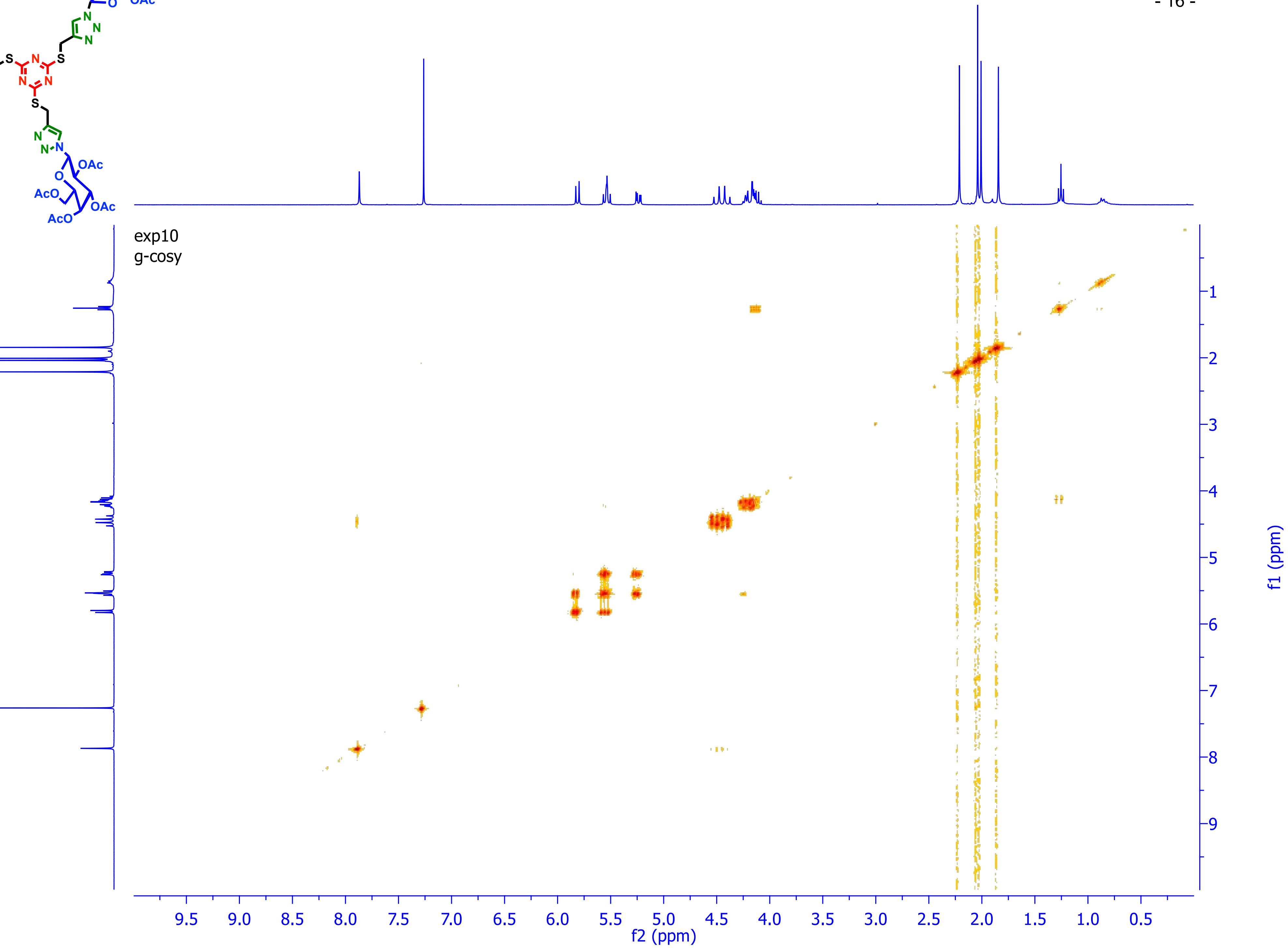
exp10
g-cosy
f1 (ppm)
f2 (ppm)
9.5 9.0 8.5 8.0 7.5 7.0 6.5 6.0 5.5 5.0 4.5 4.0 3.5 3.0 2.5 2.0 1.5 1.0 0.5
1 2 3 4 5 6 7 8 9

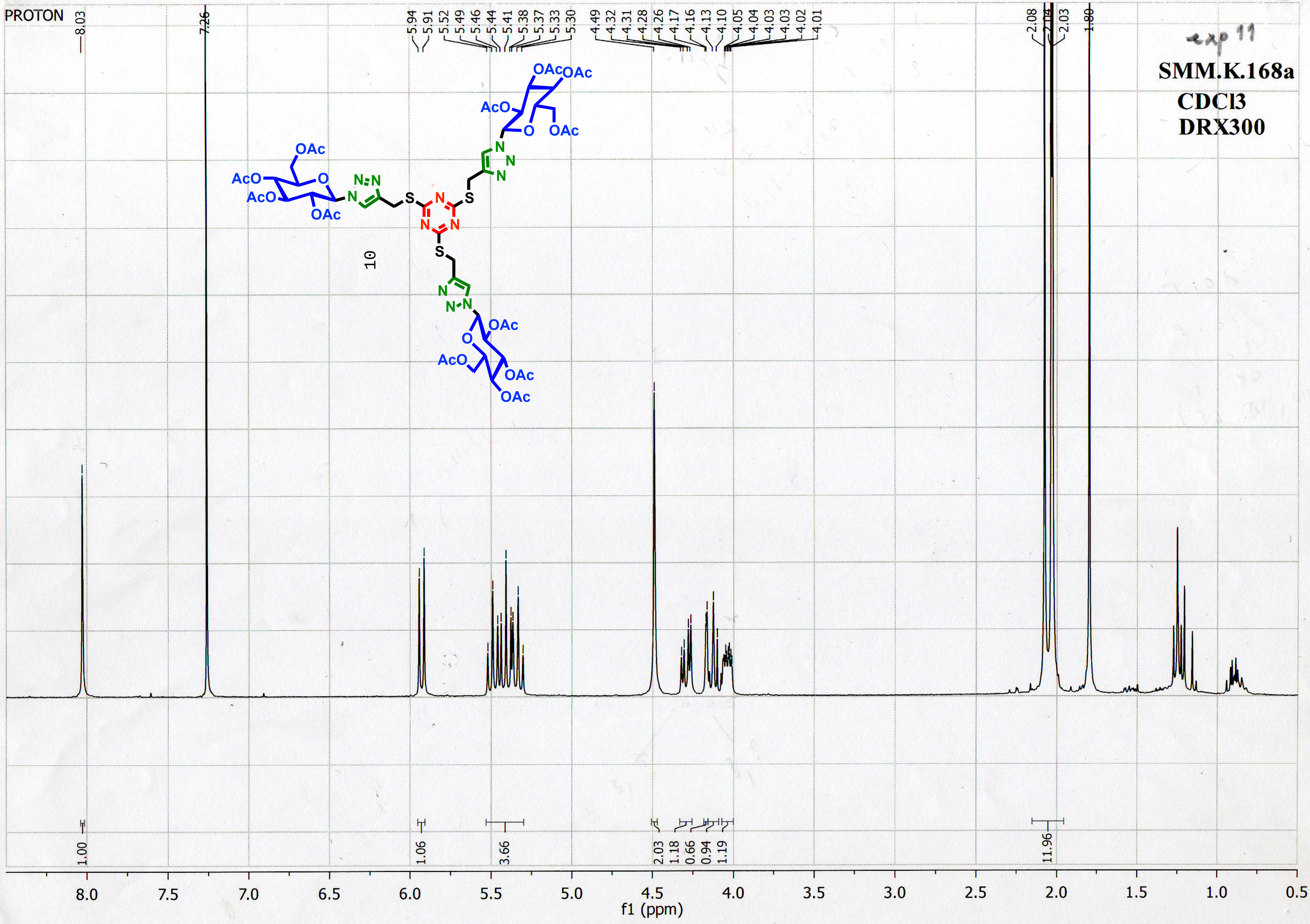
PROTON
8.03
7.26
5.94
5.91
5.52
5.49
5.46
5.44
5.41
5.38
5.37
5.33
5.30
4.49
4.32
4.31
4.28
4.26
4.17
4.16
4.13
4.10
4.05
4.04
4.03
4.03
4.02
4.01
2.08
2.04
2.03
1.80
exp 11
SMM.K.168a
CDCl3
DRX300
10
AcO
OAc
O
N
S
1.00
1.06
3.66
2.03
1.18
0.66
0.94
1.19
11.96
f1 (ppm)

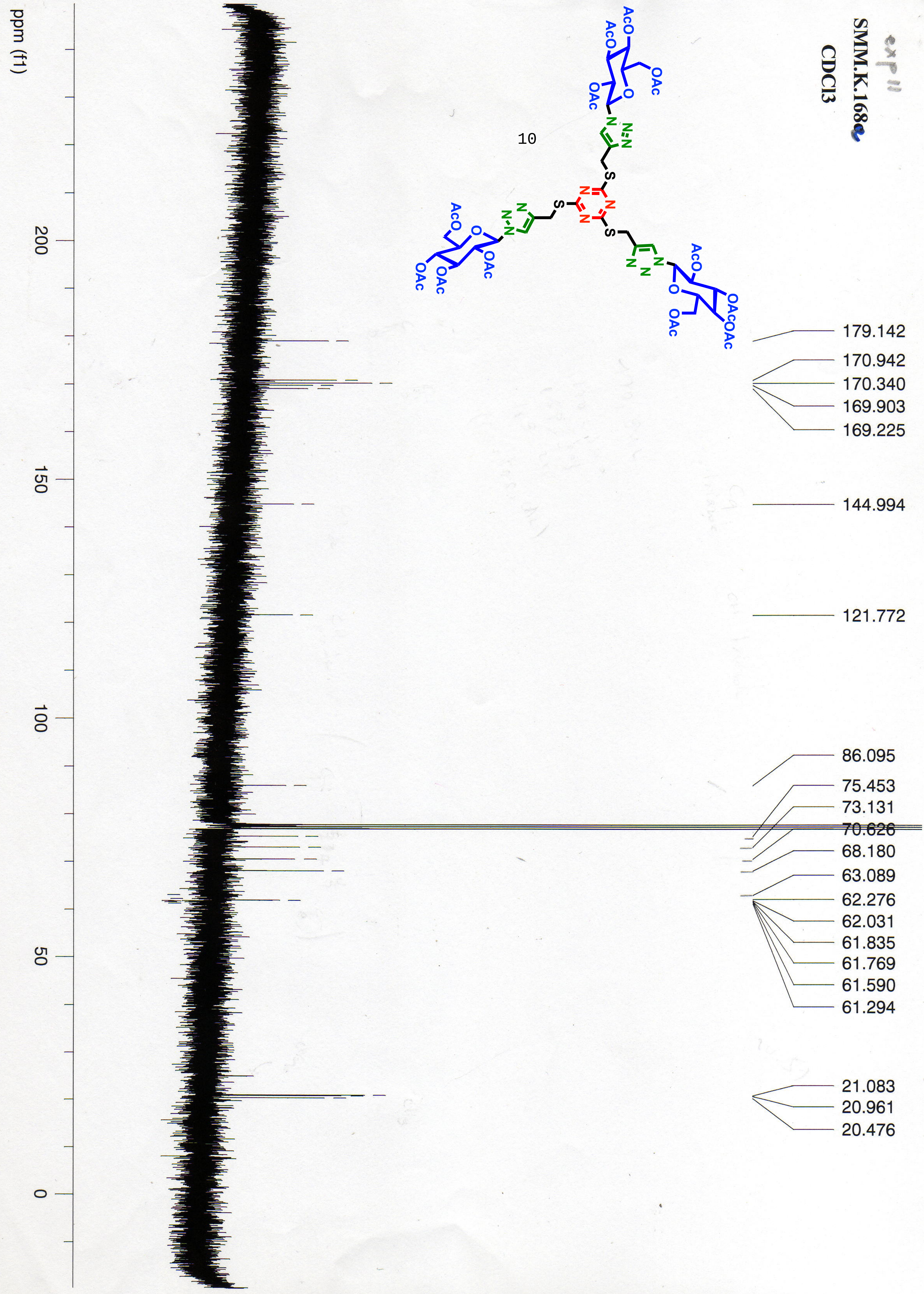
SMM.K.168
CDCl3
10
AcO
OAc
N
S
179.142
170.942
170.340
169.903
169.225
144.994
121.772
86.095
75.453
73.131
70.626
68.180
63.089
62.276
62.031
61.835
61.769
61.590
61.294
21.083
20.961
20.476
ppm (f1)
200
150
100
50
0

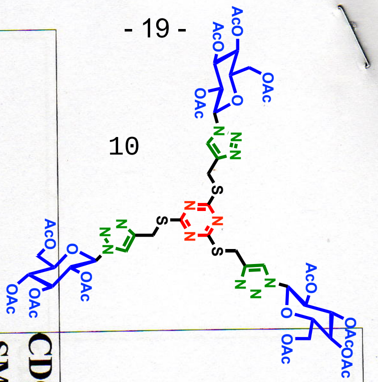

10

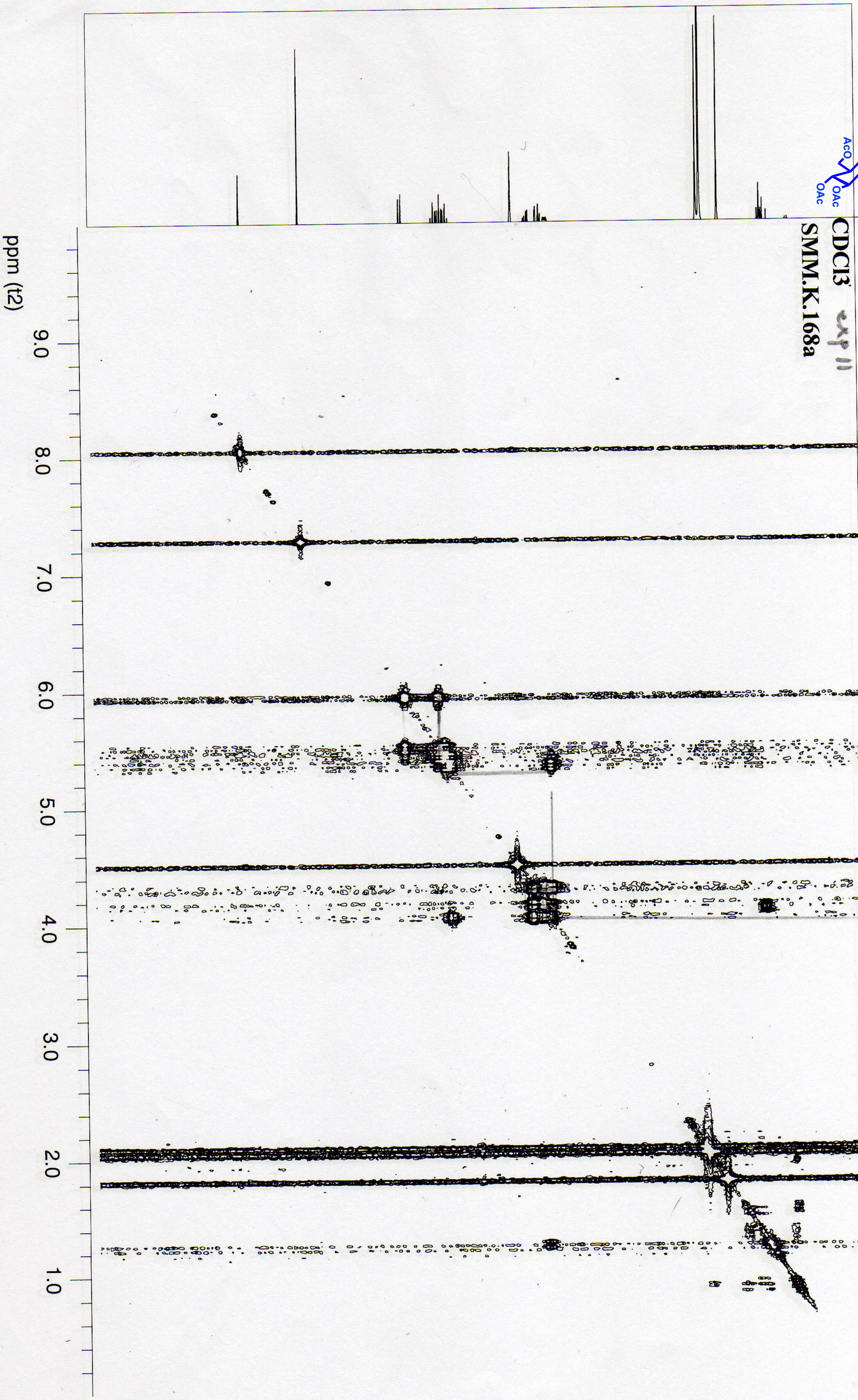

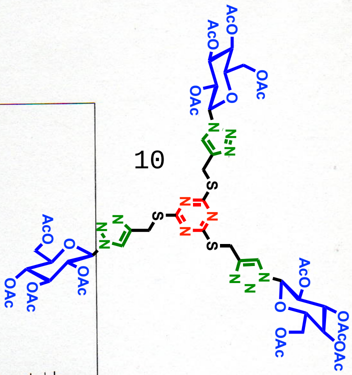

10

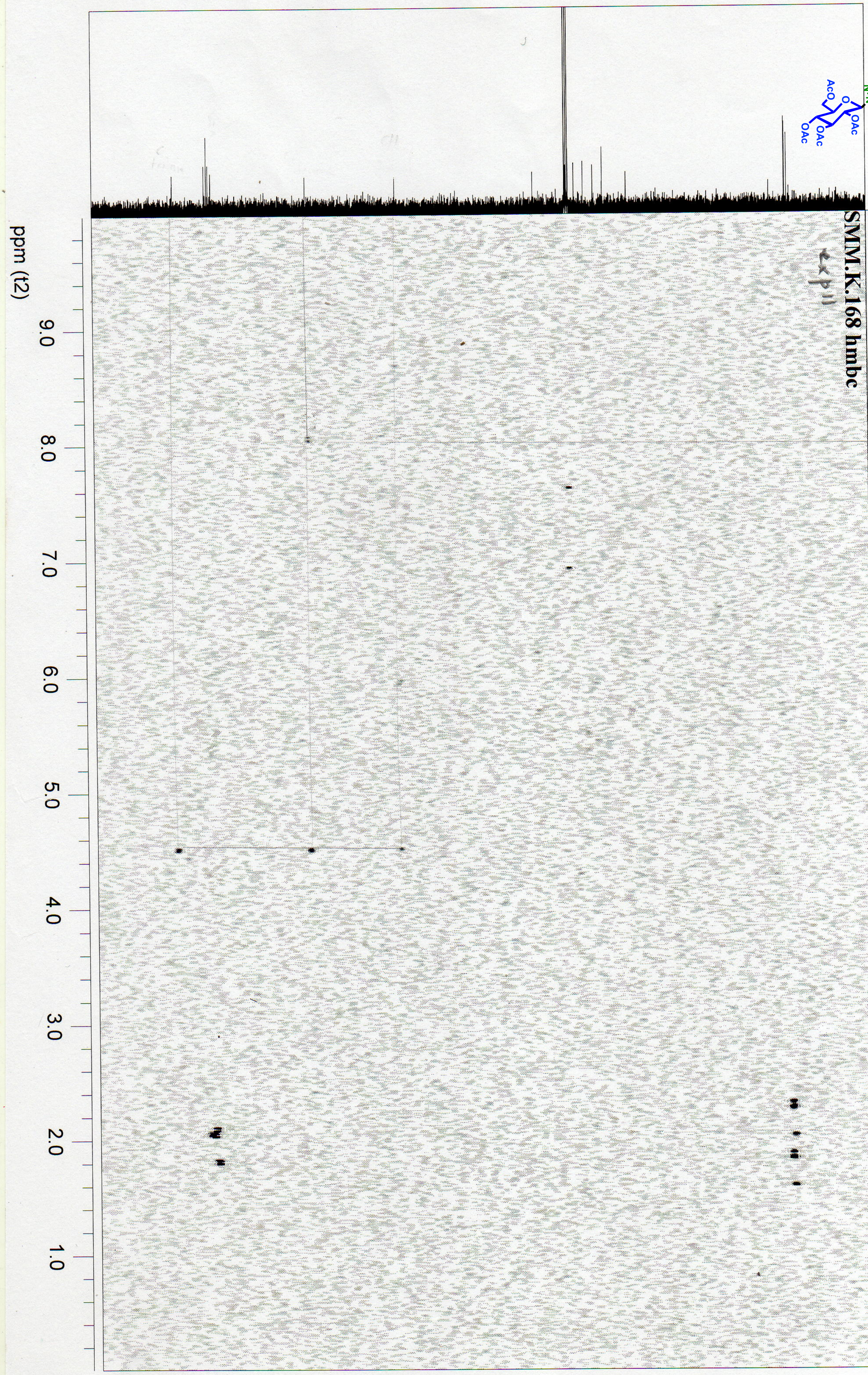

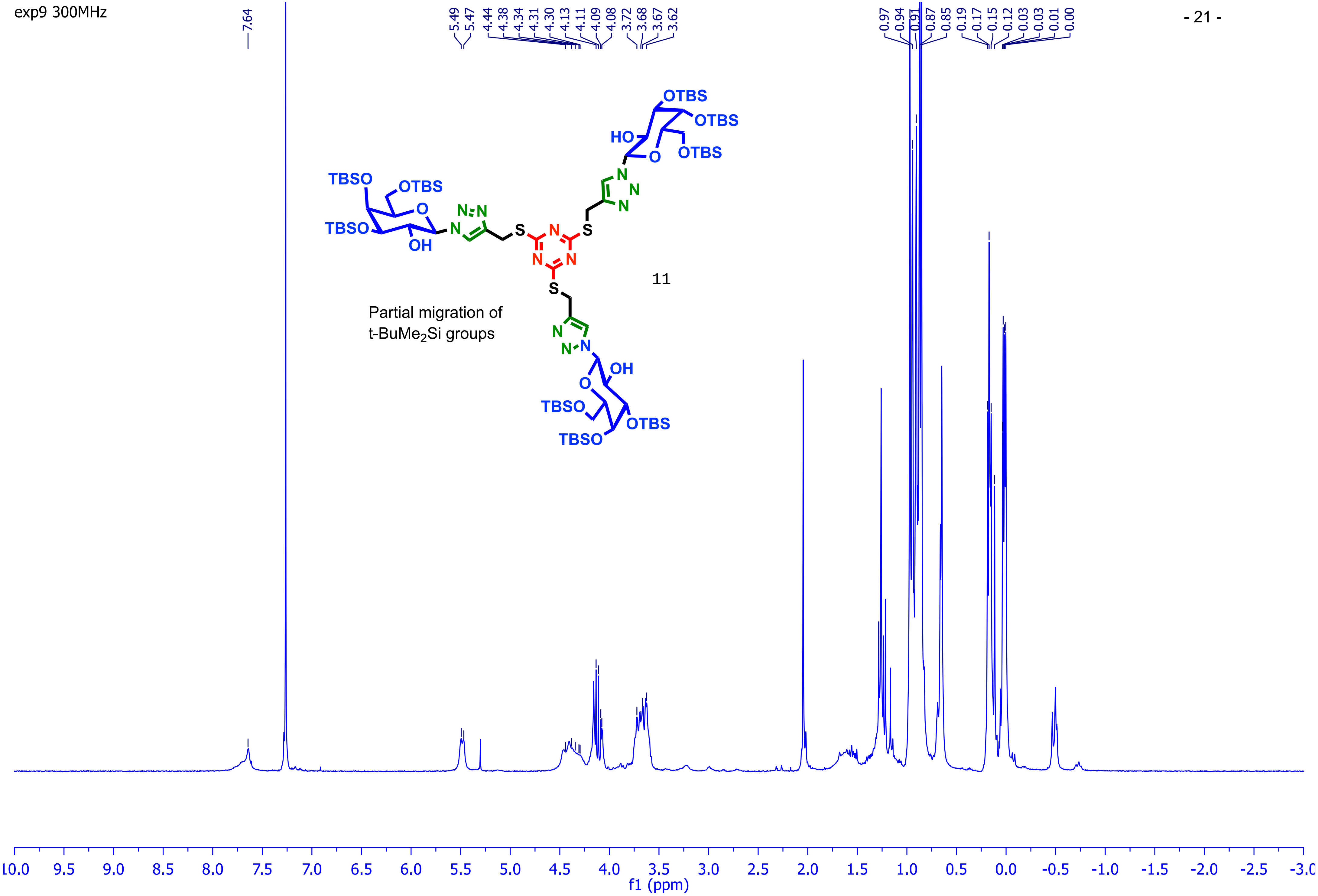
exp9 300MHz
7.64
5.49 5.47 4.44 4.38 4.34 4.31 4.30 4.13 4.11 4.09 4.08 3.72 3.68 3.67 3.62
0.97 0.94 0.91 0.87 0.85 0.19 0.17 0.15 0.12 0.03 0.03 0.01 0.00
OTBS
OTBS
HO
O
OTBS
TBSO
OTBS
N
N
N
O
N
N
TBSO
N
S
N
S
OH
N
N
N
11
S
Partial migration of
t-BuMe2Si groups
N
N
N
OH
O
TBSO
OTBS
TBSO
10.0 9.5 9.0 8.5 8.0 7.5 7.0 6.5 6.0 5.5 5.0 4.5 4.0 3.5 3.0 2.5 2.0 1.5 1.0 0.5 0.0 -0.5 -1.0 -1.5 -2.0 -2.5 -3.0
f1 (ppm)

exp 8
SHM0.89 / LCO2 PG
PROTON CDCl3 v3 CCRMN 2

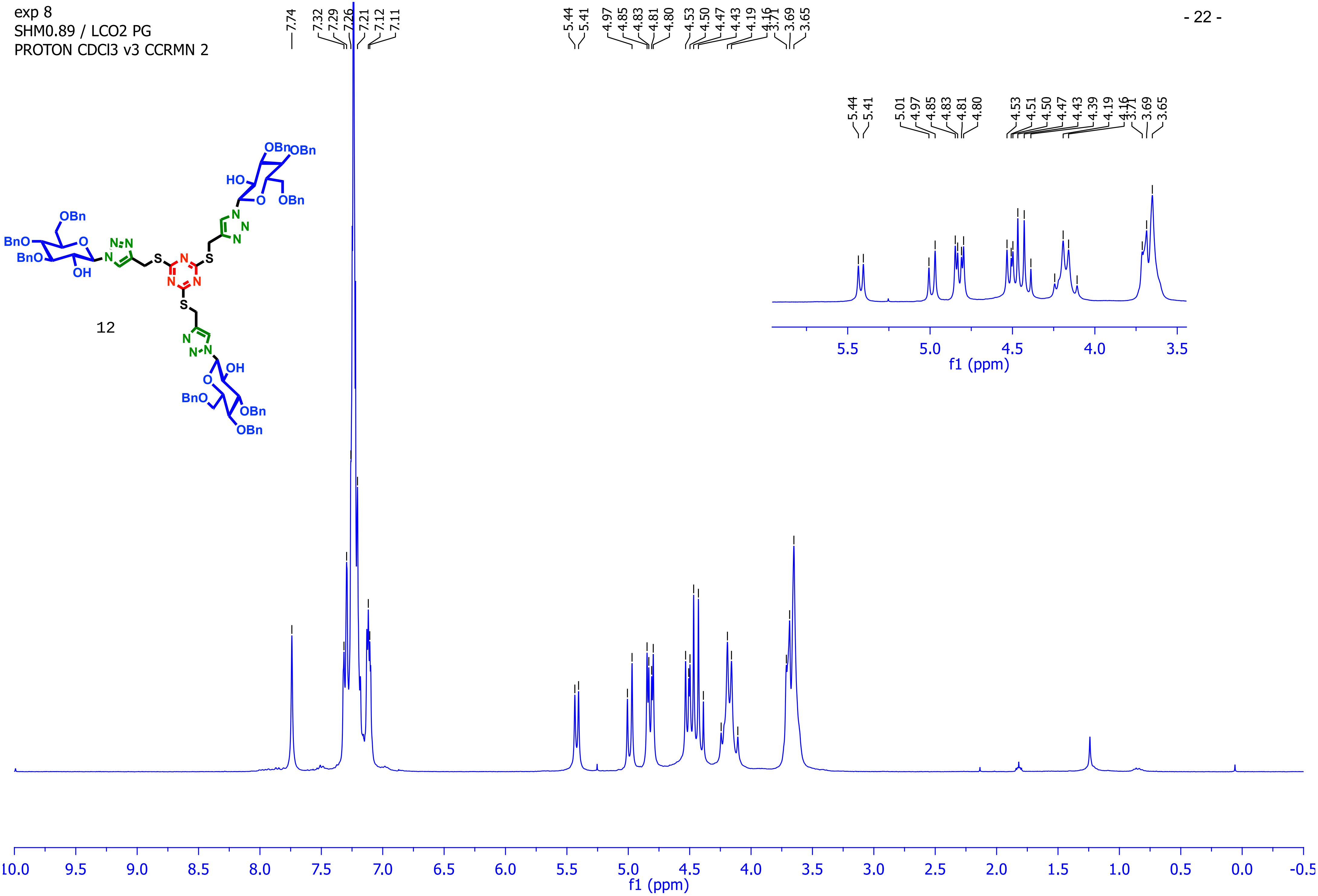

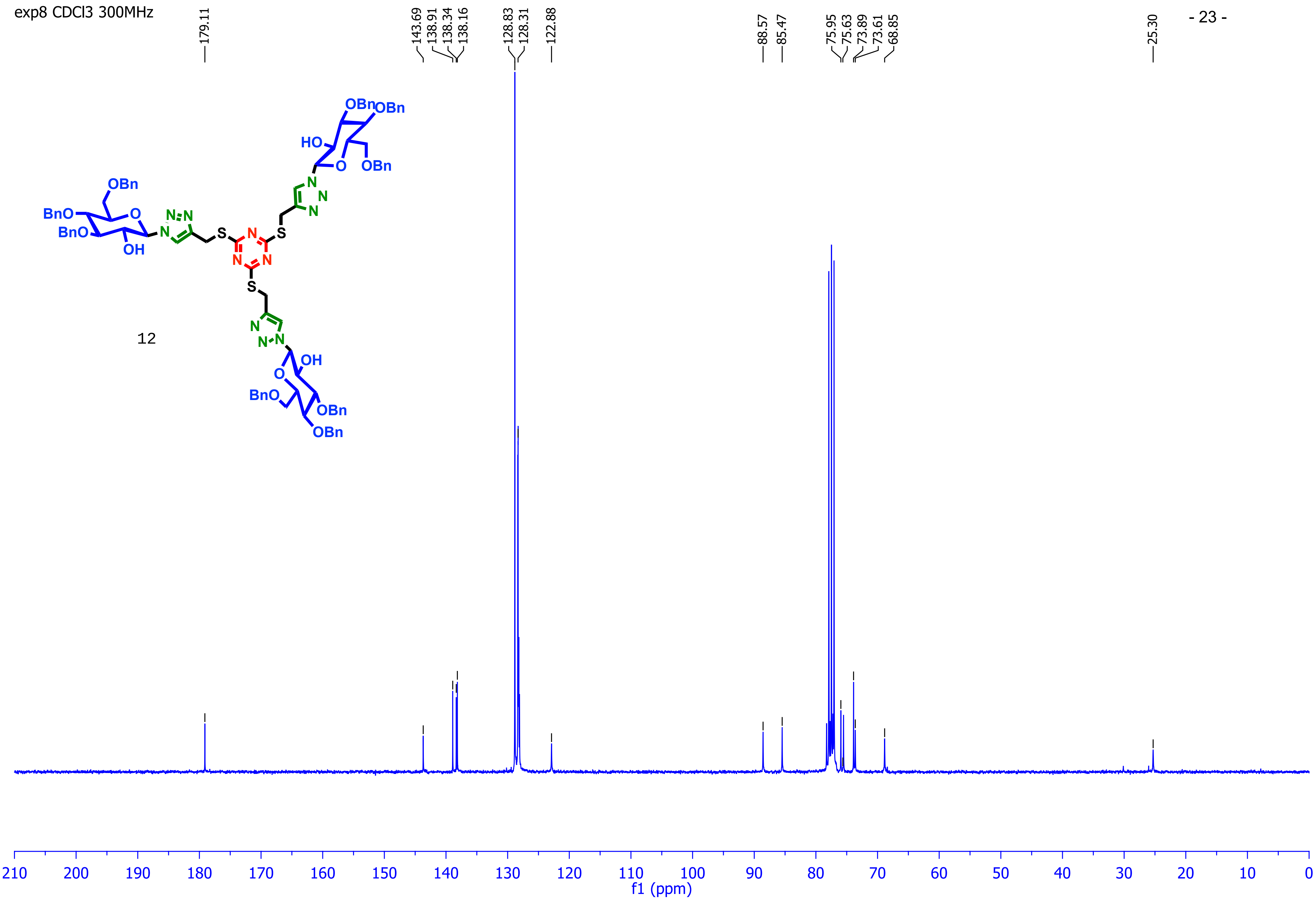
exp8 CDCl3 300MHz
179.11
143.69
138.91
138.34
138.16
128.83
128.31
122.88
88.57
85.47
75.95
75.63
73.89
73.61
68.85
25.30
OBn
BnO
HO
OH
O
N
S
12
210 200 190 180 170 160 150 140 130 120 110 100 90 80 70 60 50 40 30 20 10 0
f1 (ppm)

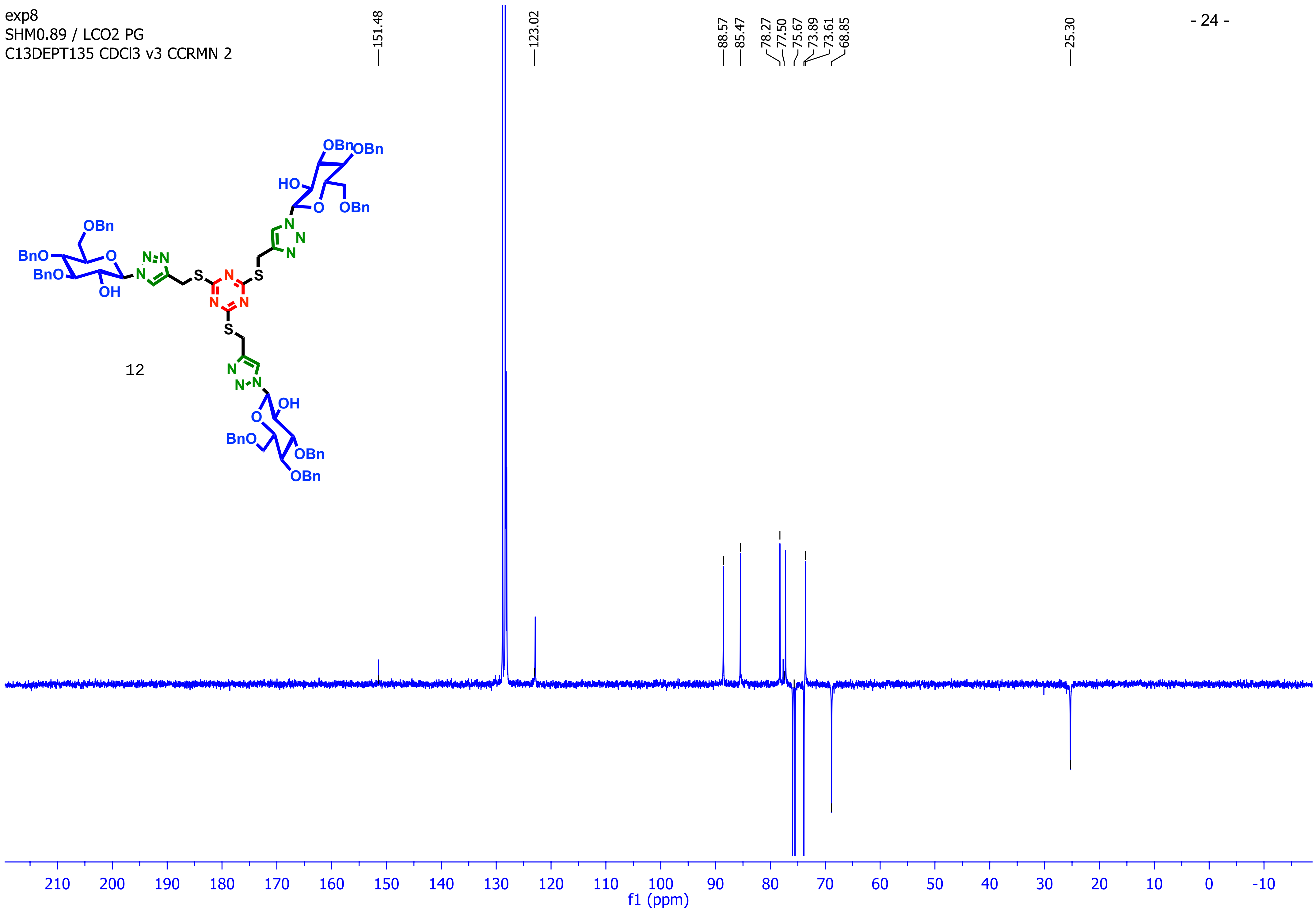
exp8
SHM0.89 / LCO2 PG
C13DEPT135 CDCl3 v3 CCRMN 2
151.48
123.02
88.57
85.47
78.27
77.50
75.67
73.89
73.61
68.85
25.30
OBn
OBn
HO
O
OBn
OBn
BnO
O
N
N
N
BnO
OH
S
S
N
N
N
N
N
N
S
12
N
N
N
OH
O
BnO
OBn
OBn
210
200
190
180
170
160
150
140
130
120
110
100
90
80
70
60
50
40
30
20
10
0
-10
f1 (ppm)

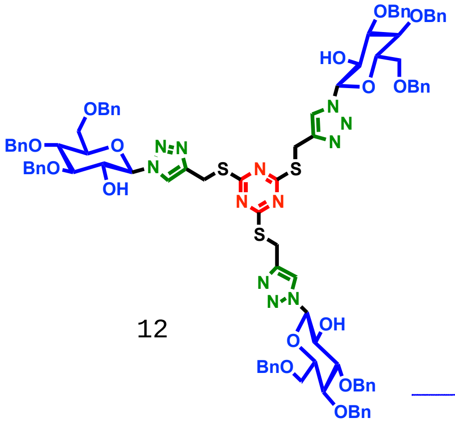
OBn
OBn
HO
O
OBn
N
N
N
OBn
BnO
O
BnO
N
N
N
N
S
N
S
OH
N
N
N
S
S
N
N
N
OH
O
BnO
OBn
OBn

12

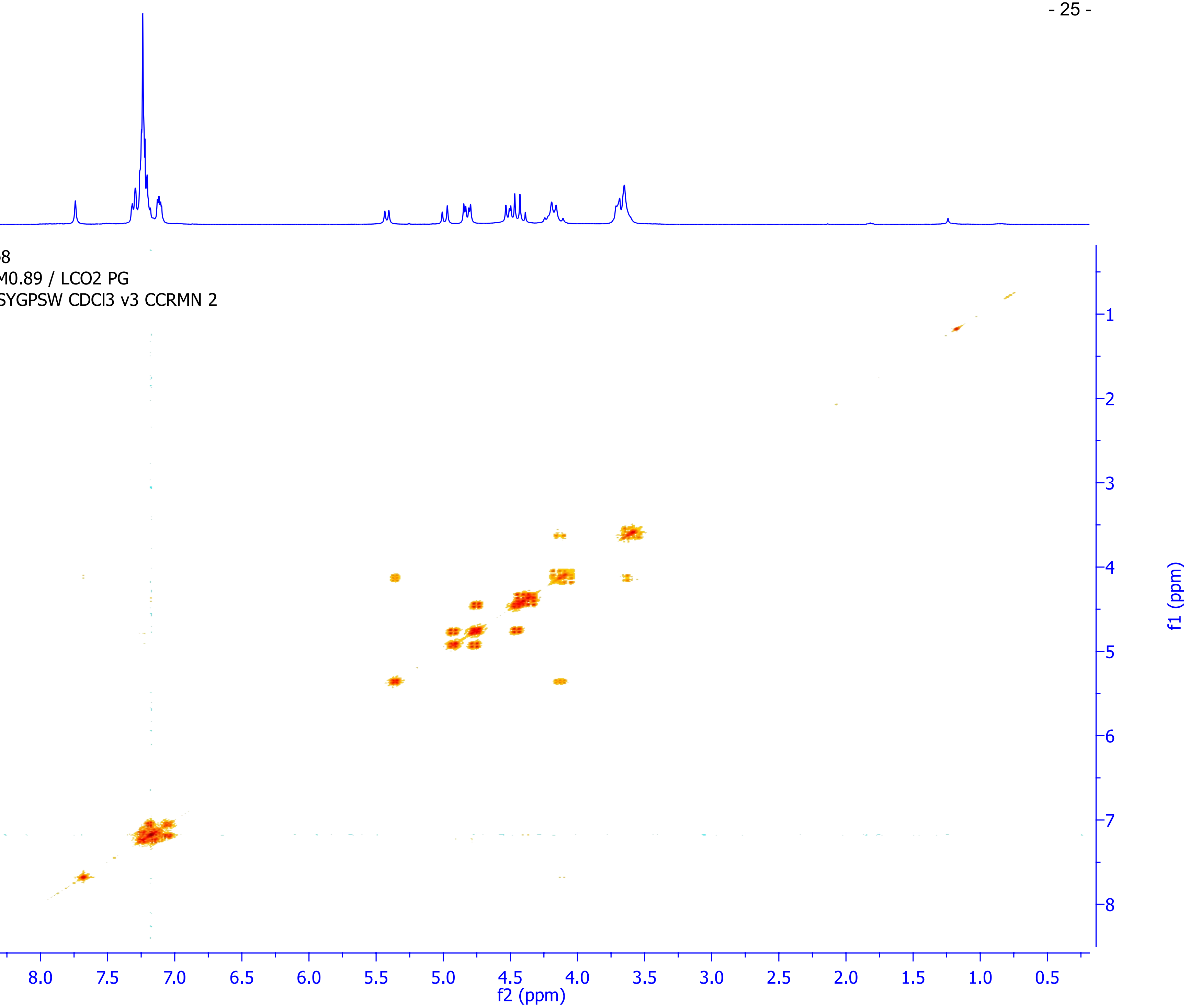
exp8
SHM0.89 / LCO2 PG
COSYGPSW CDCl3 v3 CCRMN 2
f1 (ppm)
f2 (ppm)
8.0
7.5
7.0
6.5
6.0
5.5
5.0
4.5
4.0
3.5
3.0
2.5
2.0
1.5
1.0
0.5
1
2
3
4
5
6
7
8

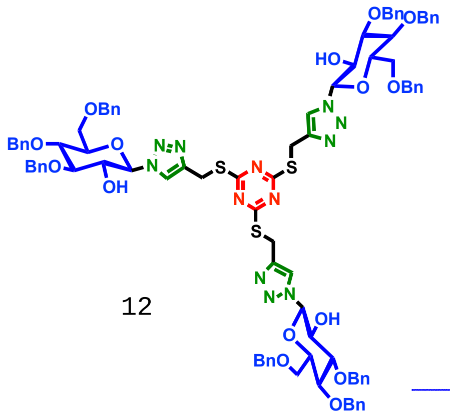
OBn
OBn
HO
O
OBn
OBn
BnO
BnO
O
OH
N=N
N
N
N
N
S
S
S
N
N
N
N
N
N
N
OH
O
BnO
OBn
OBn

12

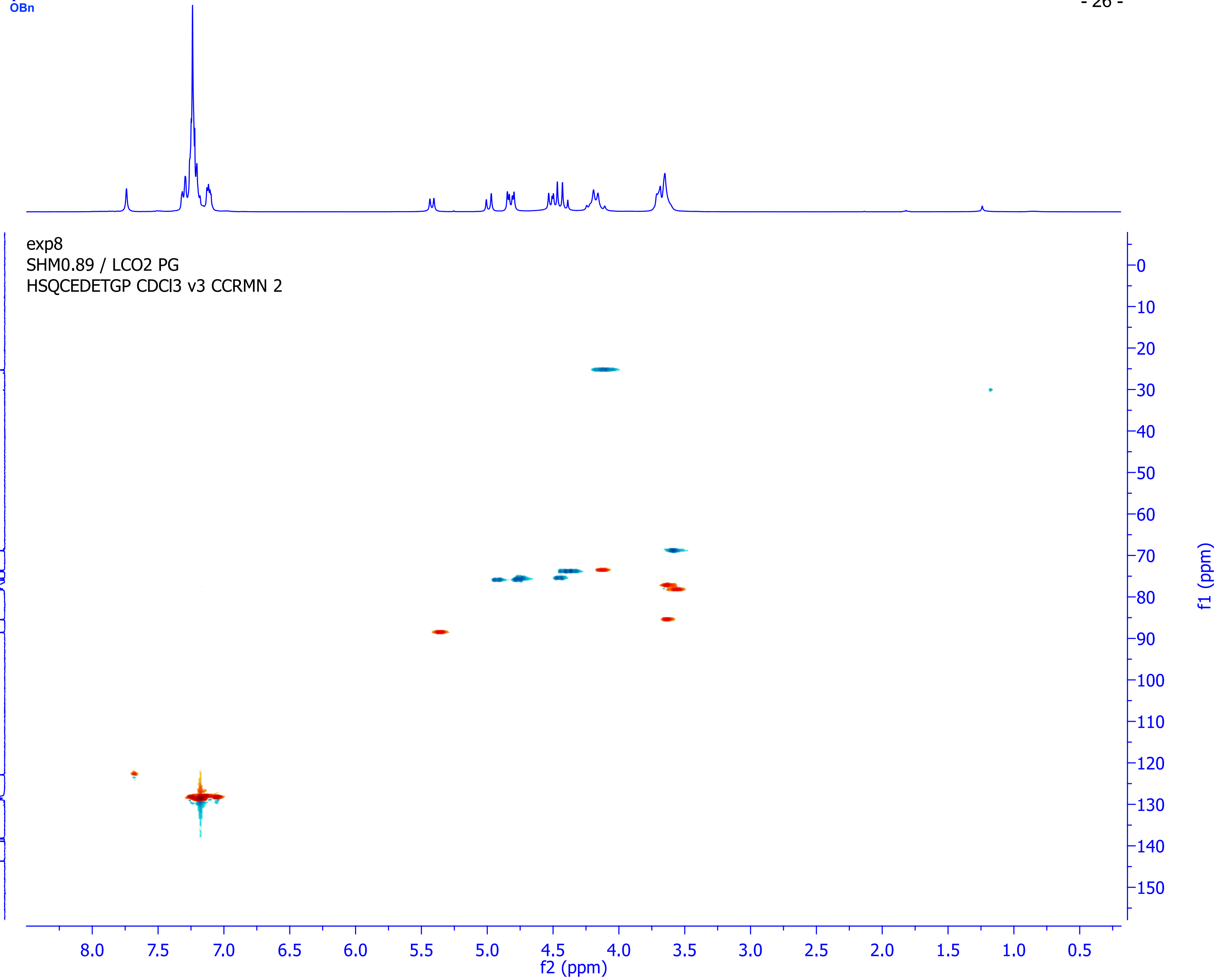
exp8
SHM0.89 / LCO2 PG
HSQCEDETGP CDCl3 v3 CCRMN 2
8.0 7.5 7.0 6.5 6.0 5.5 5.0 4.5 4.0 3.5 3.0 2.5 2.0 1.5 1.0 0.5
f2 (ppm)
0 10 20 30 40 50 60 70 80 90 100 110 120 130 140 150
f1 (ppm)

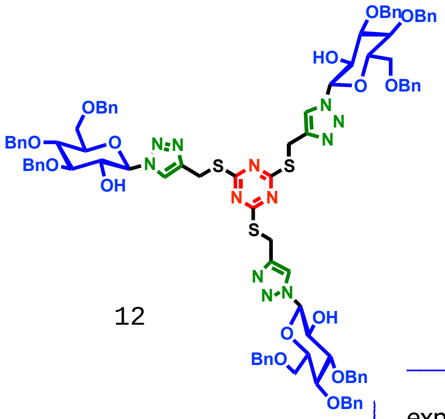
OBn
OBn
HO
O
OBn
OBn
BnO
BnO
O
N
N
N
OH
S
S
N
N
N
N
N
N
S
N
N
N
OH
O
BnO
OBn
OBn

12

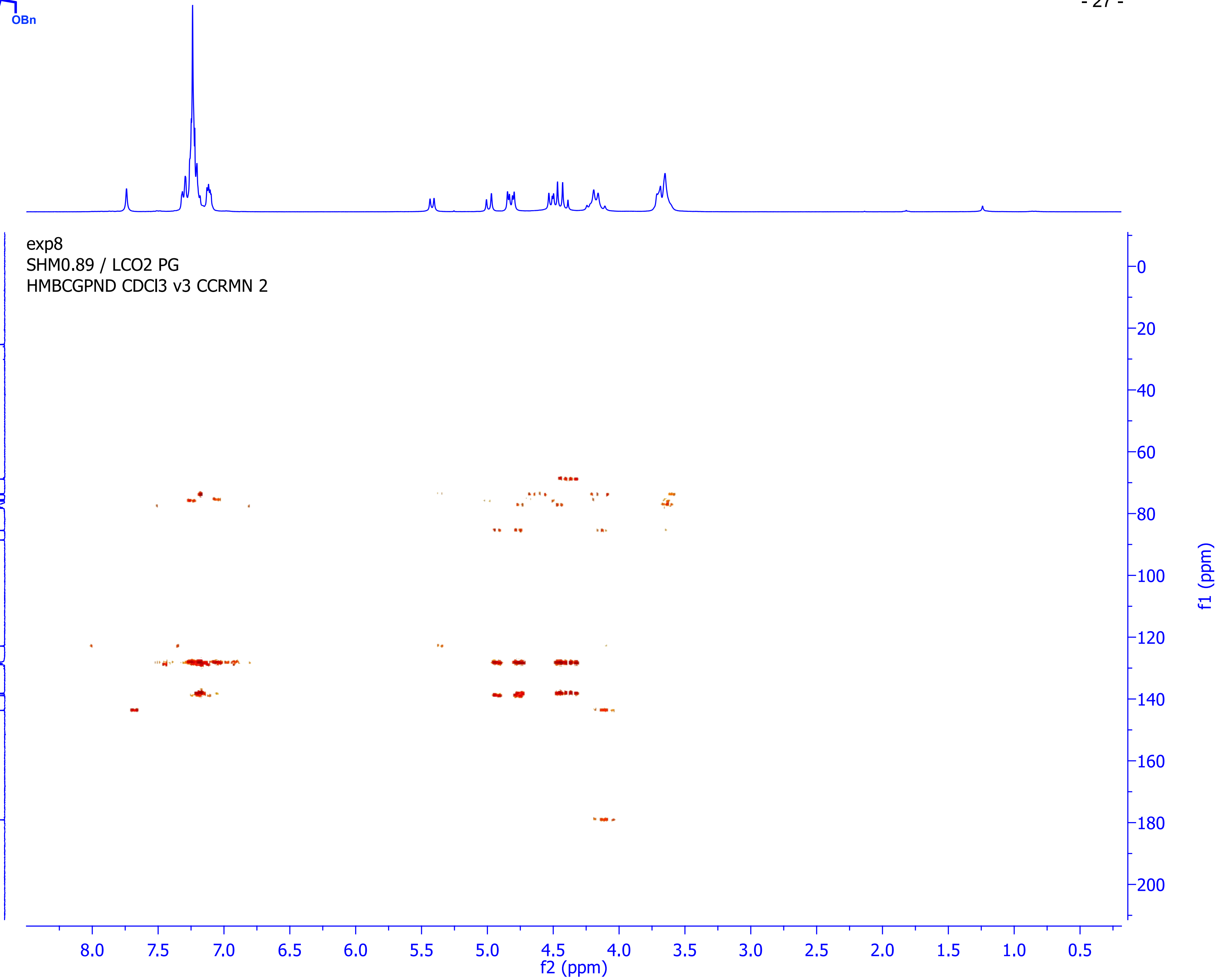
exp8
SHM0.89 / LCO2 PG
HMBCGPND CDCl3 v3 CCRMN 2
f1 (ppm)
f2 (ppm)
0
20
40
60
80
100
120
140
160
180
200
8.0
7.5
7.0
6.5
6.0
5.5
5.0
4.5
4.0
3.5
3.0
2.5
2.0
1.5
1.0
0.5

exp 5 DMSO 300 MHz

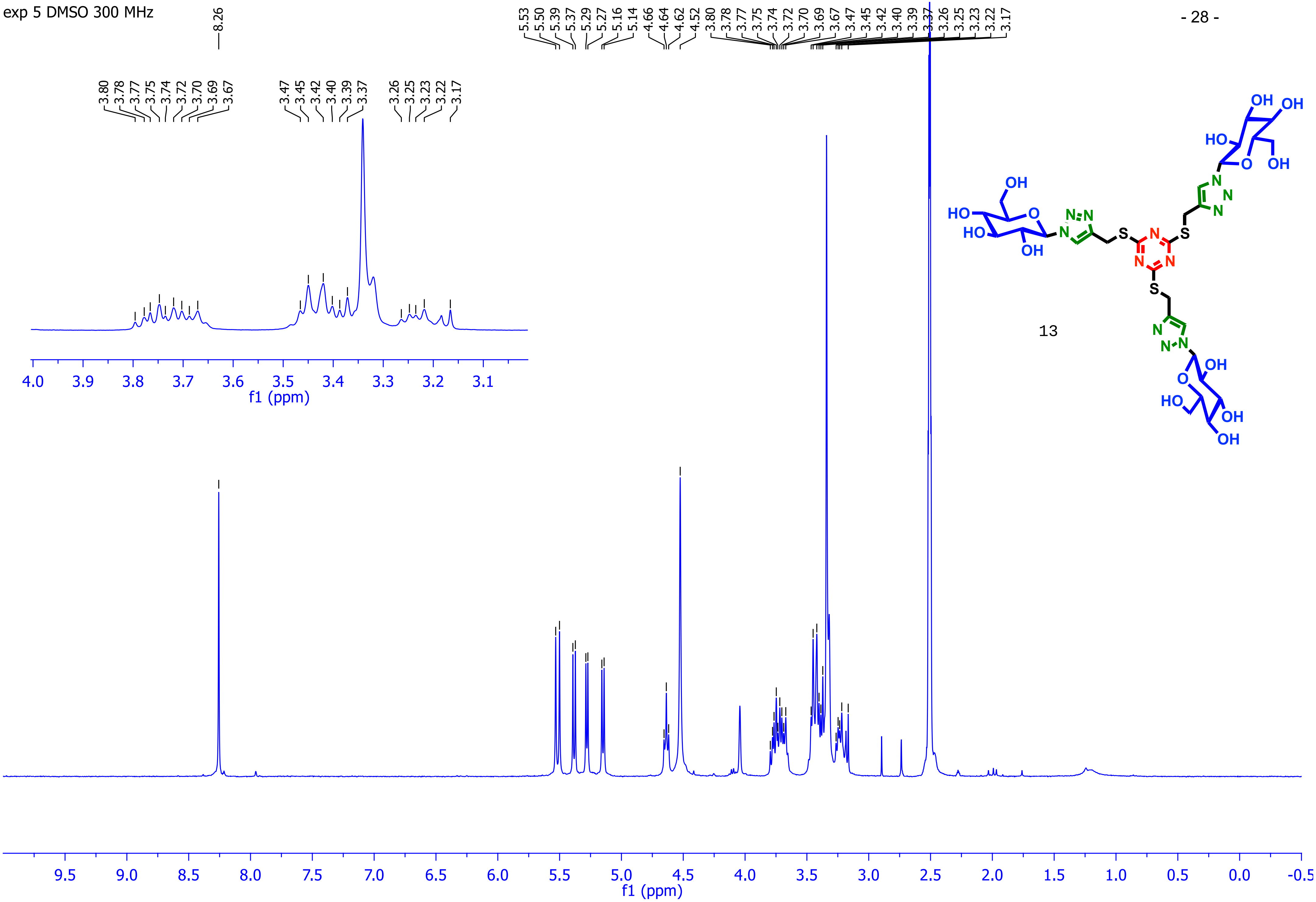

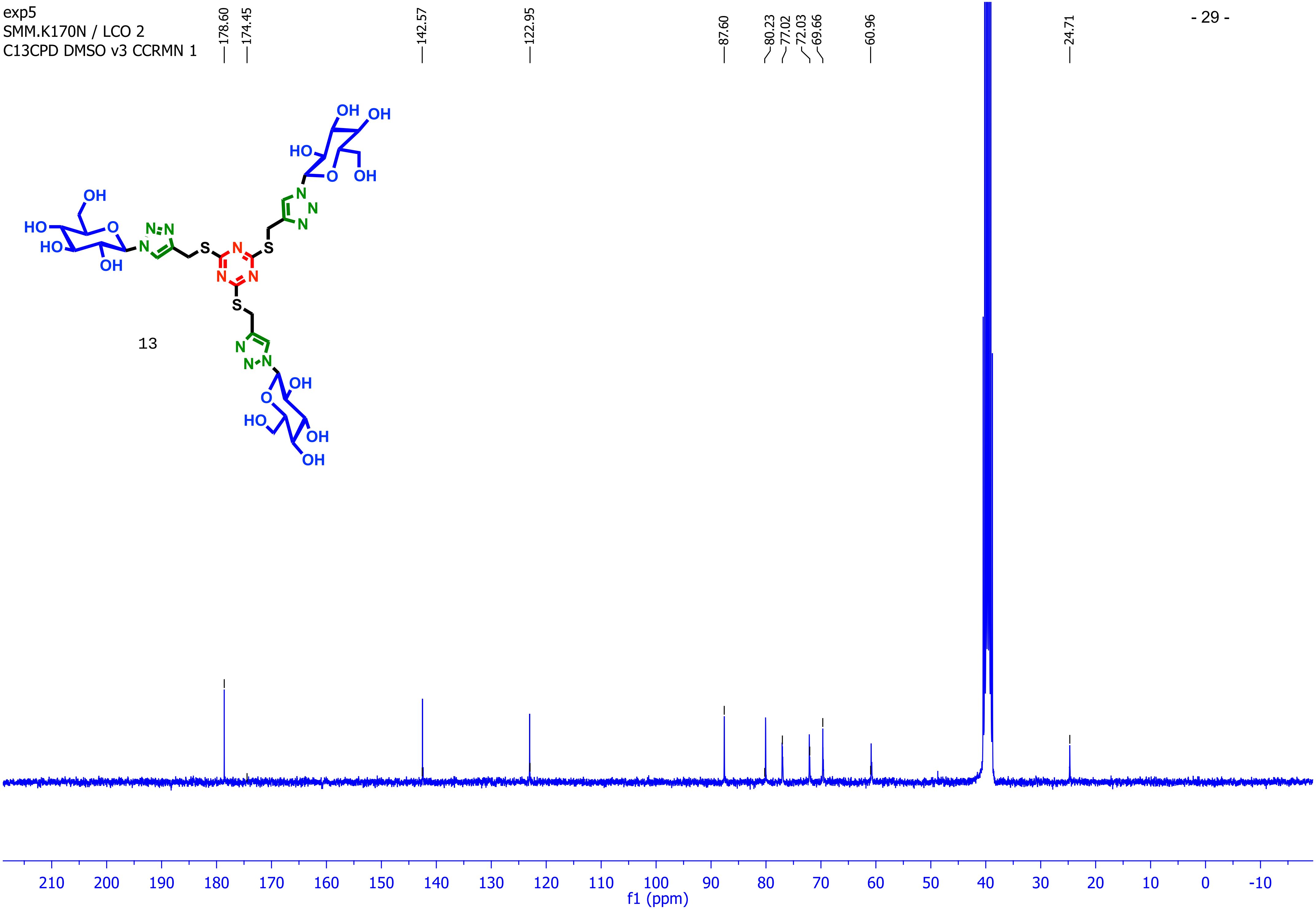
exp5
SMM.K170N / LCO 2
C13CPD DMSO v3 CCRMN 1
178.60
174.45
142.57
122.95
87.60
80.23
77.02
72.03
69.66
60.96
24.71
13
210 200 190 180 170 160 150 140 130 120 110 100 90 80 70 60 50 40 30 20 10 0 -10
f1 (ppm)

exp 5
SMM.K170N / LCO 2
C13DEPT135 DMSO v3 CCRMN 1

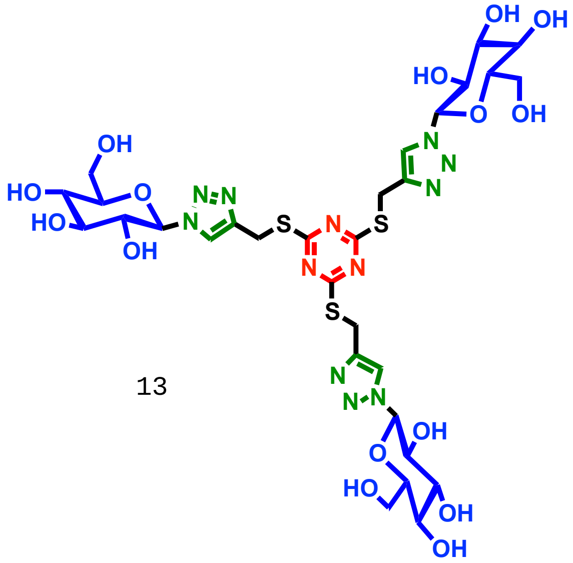

13

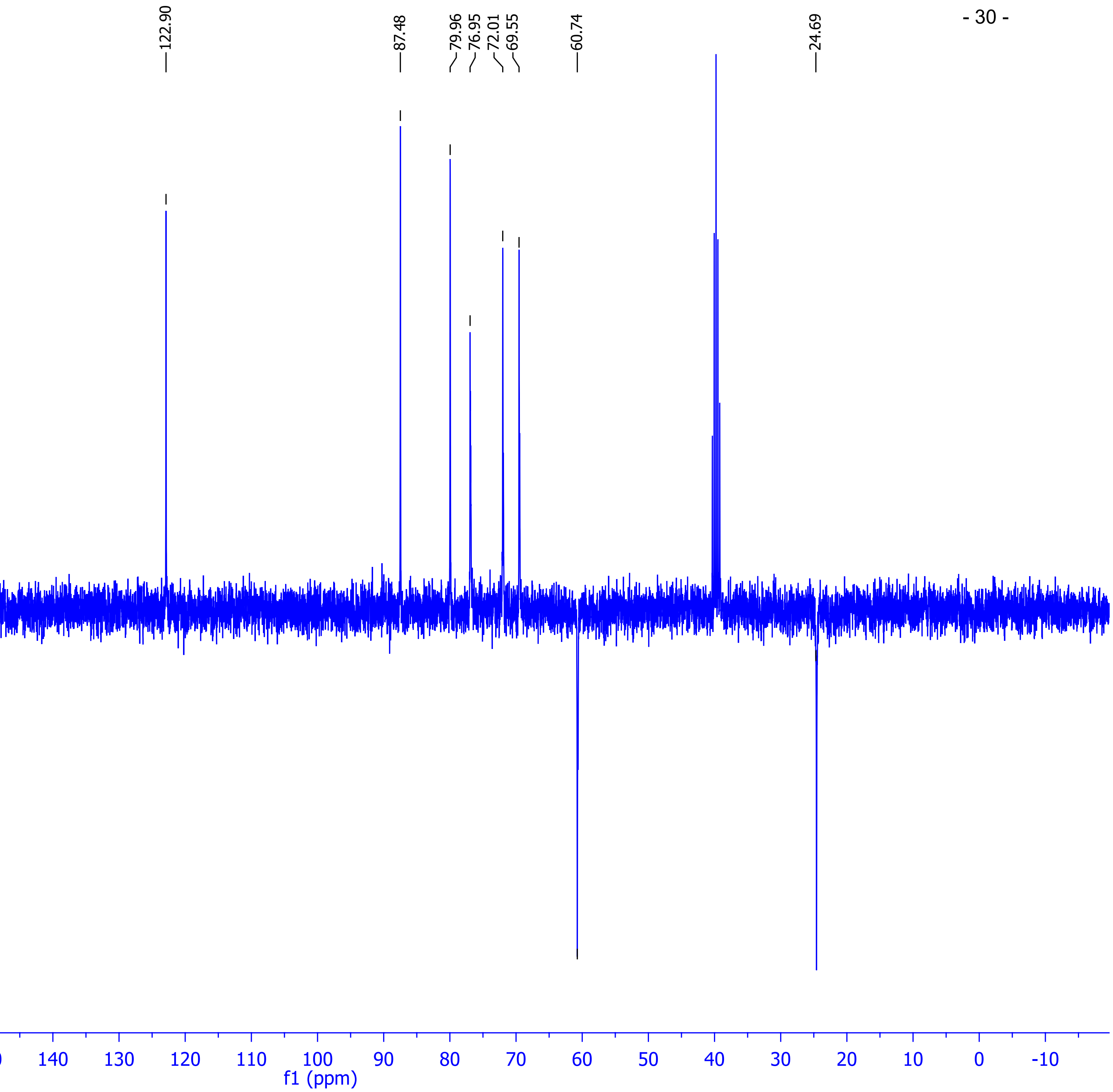

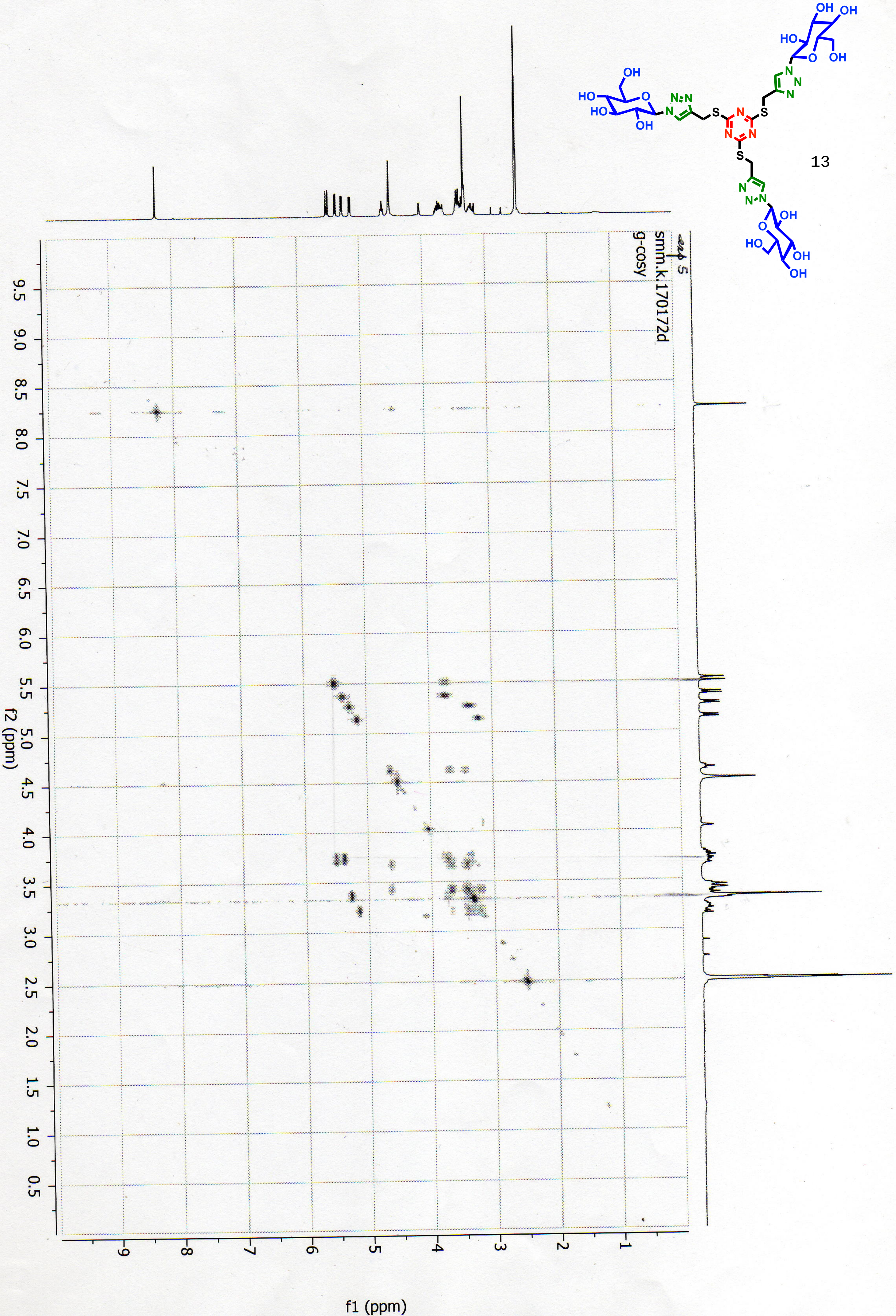

13

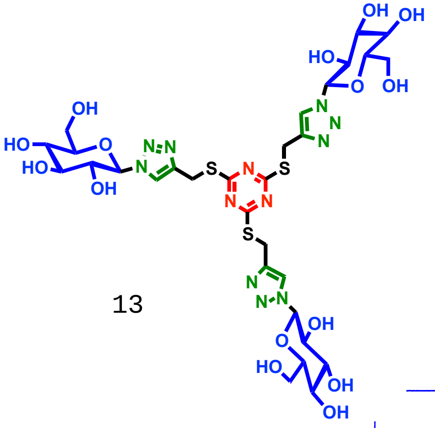

13

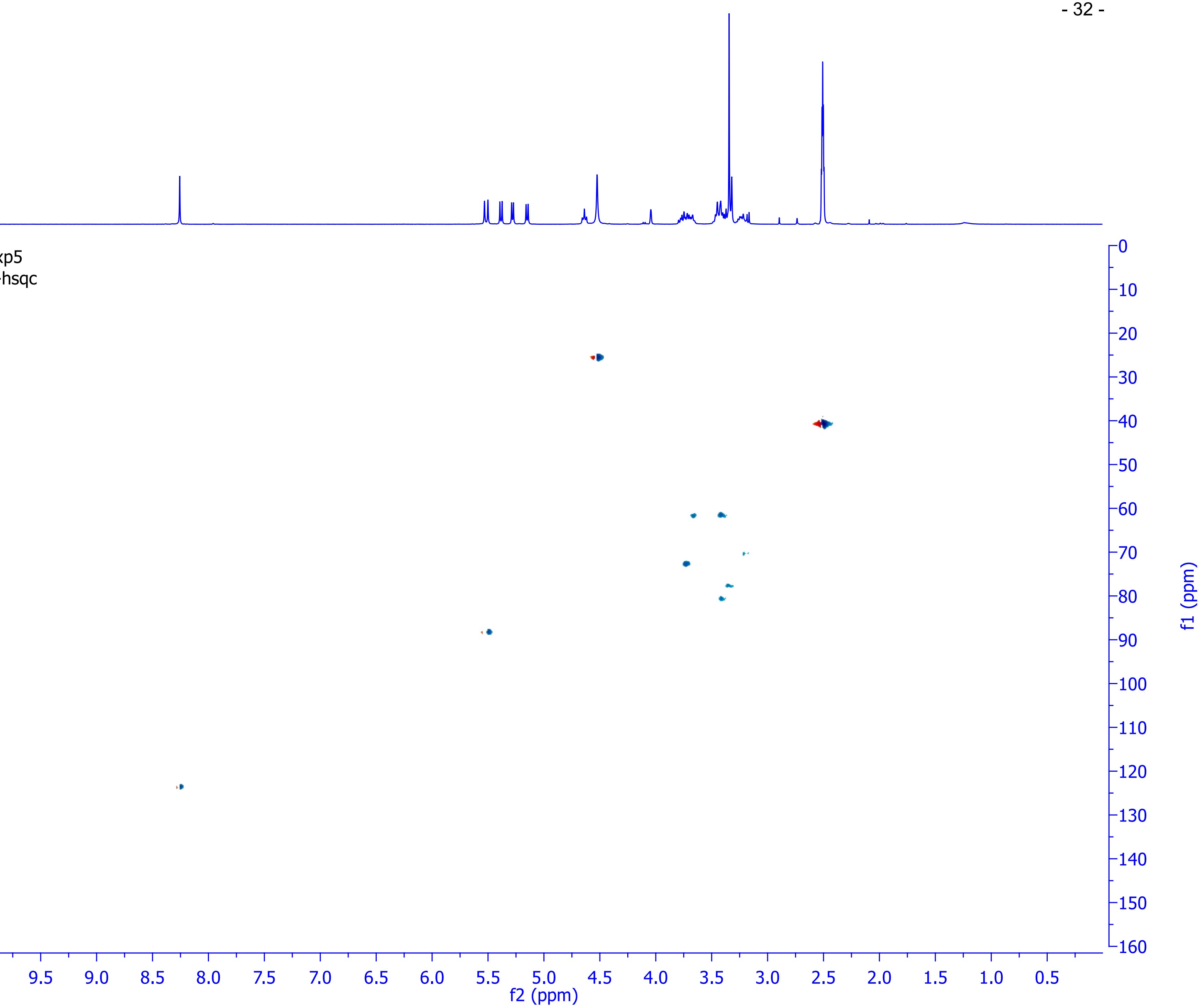

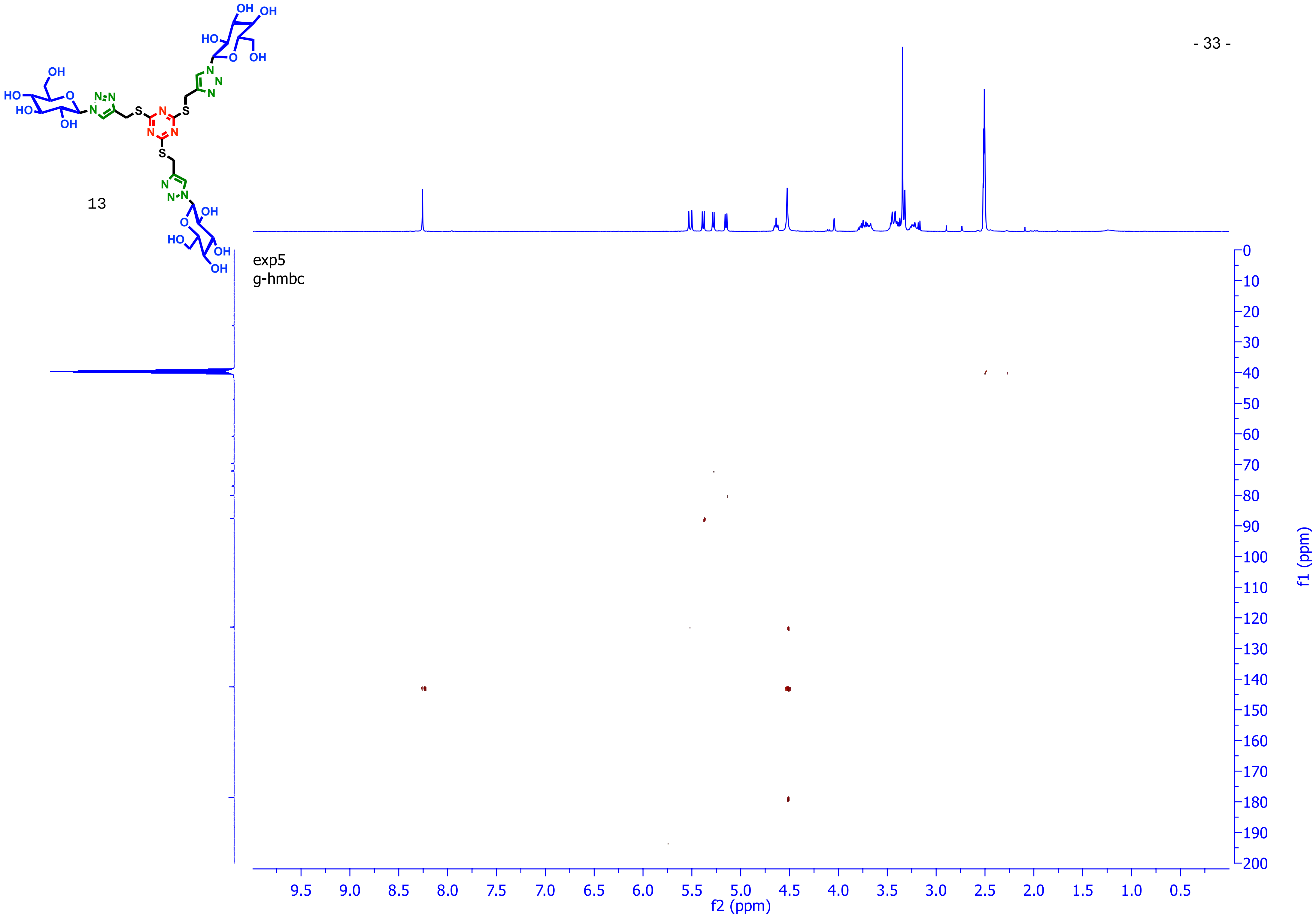
13
exp5
g-hmbc
f2 (ppm)
f1 (ppm)

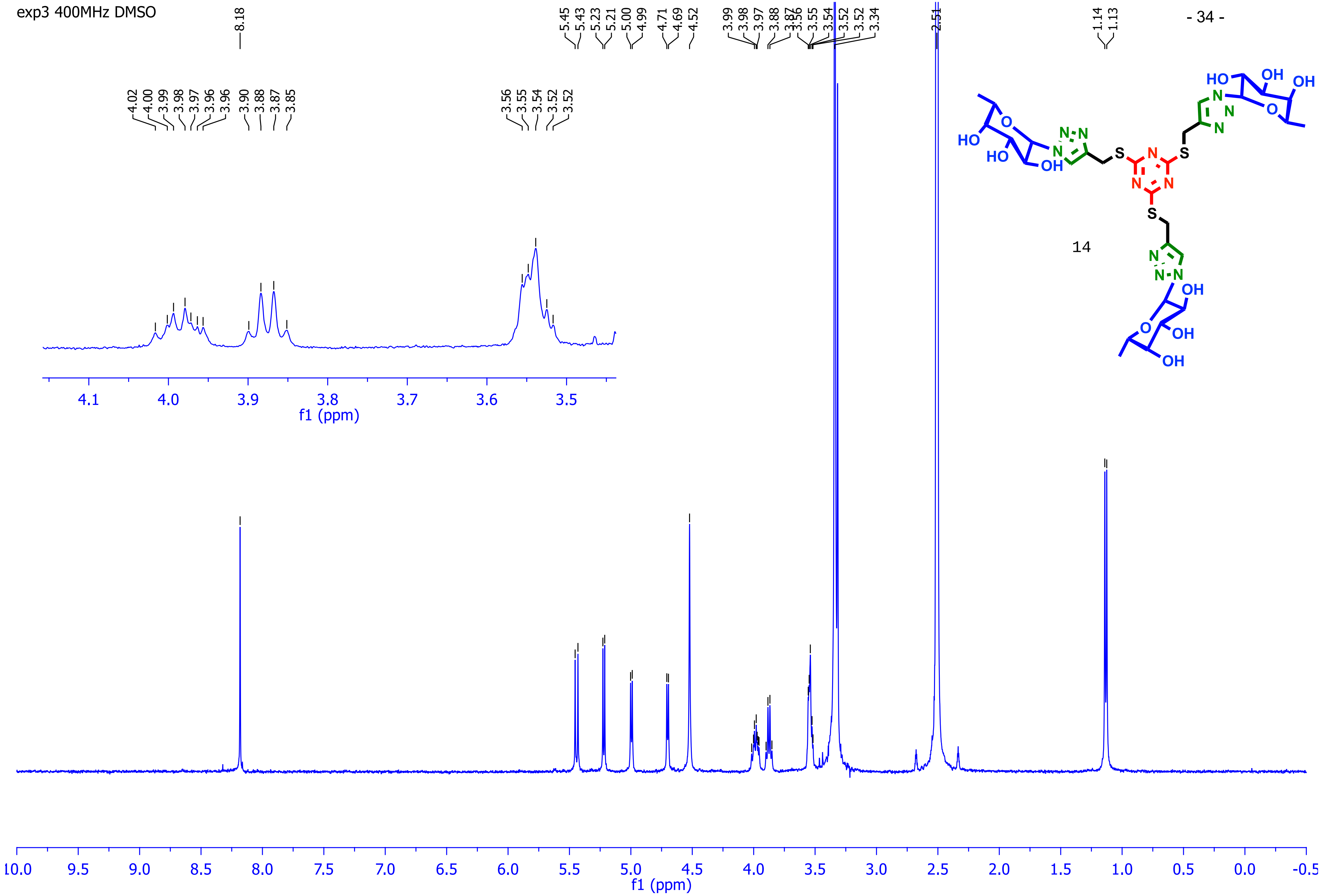
exp3 400MHz DMSO
8.18
5.45
5.43
5.23
5.21
5.00
4.99
4.71
4.69
4.52
3.99
3.98
3.97
3.88
3.87
3.56
3.55
3.54
3.52
3.52
3.34
2.51
1.14
1.13
4.02
4.00
3.99
3.98
3.97
3.96
3.96
3.90
3.88
3.87
3.85
3.56
3.55
3.54
3.52
3.52
4.1
4.0
3.9
3.8
3.7
3.6
3.5
f1 (ppm)
HO
OH
OH
O
N
N
N
HO
HO
OH
S
S
S
OH
OH
OH
14
10.0
9.5
9.0
8.5
8.0
7.5
7.0
6.5
6.0
5.5
5.0
4.5
4.0
3.5
3.0
2.5
2.0
1.5
1.0
0.5
0.0
-0.5
f1 (ppm)

exp3 DMSO 400MHz

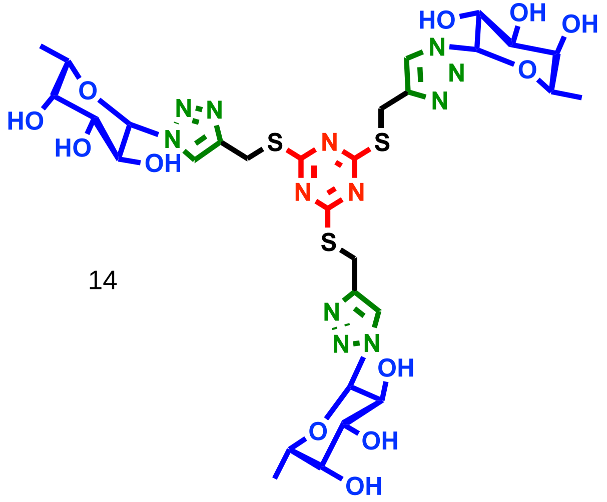

14

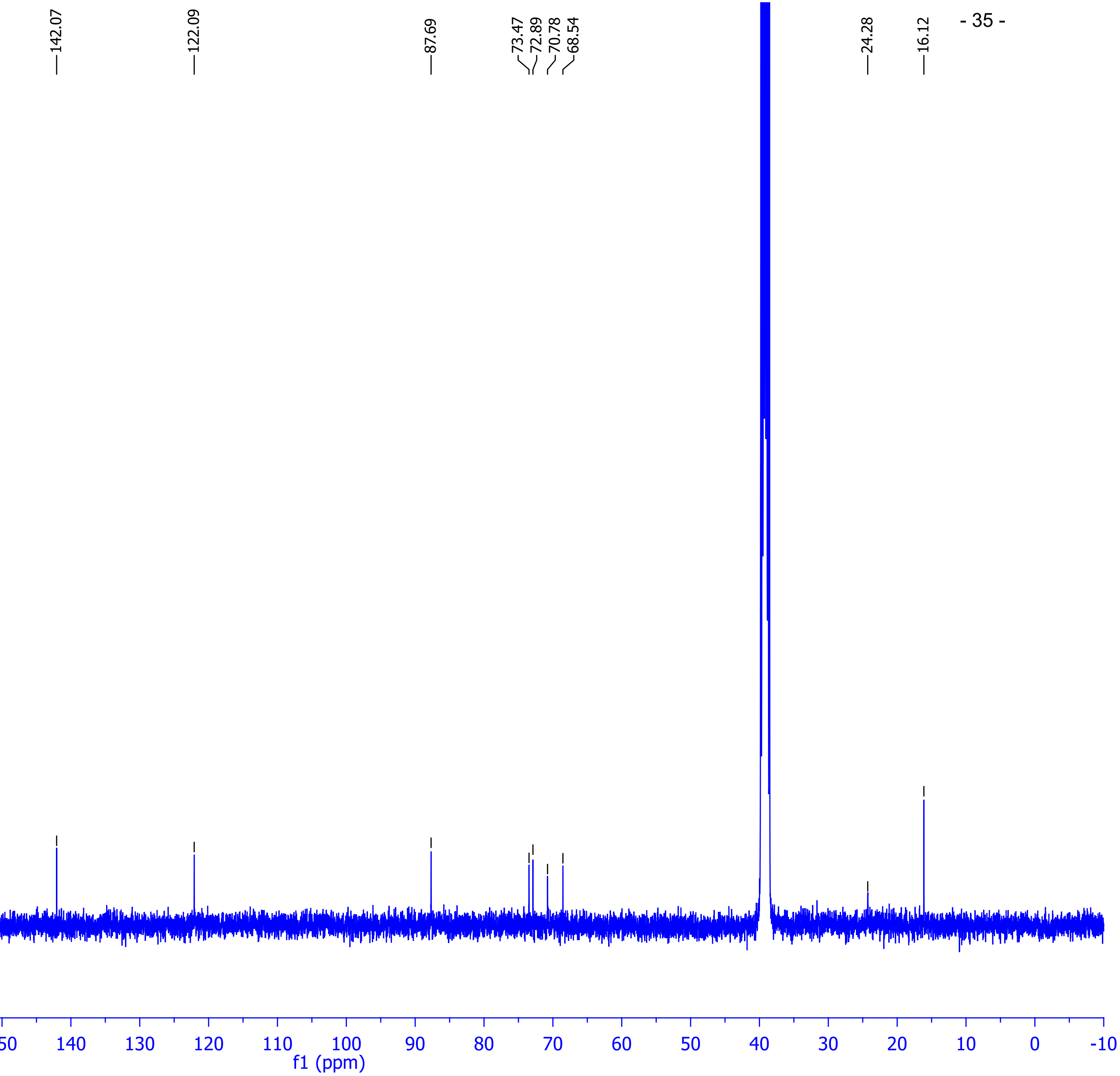

expl3
SMM_02.83a / LCO2 PG
C13DEPT135 DMSO v4 CCRMN 6

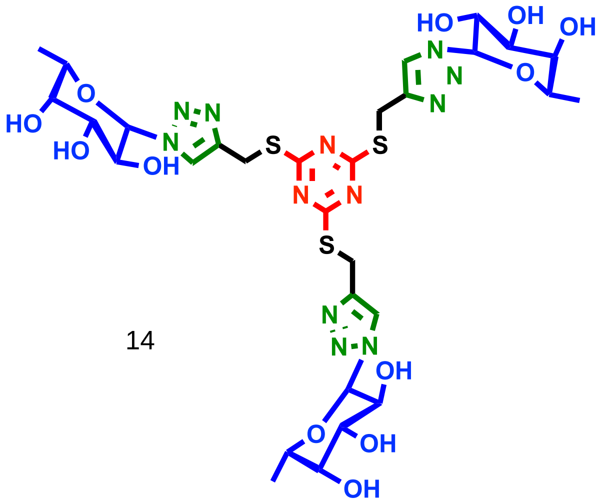

14

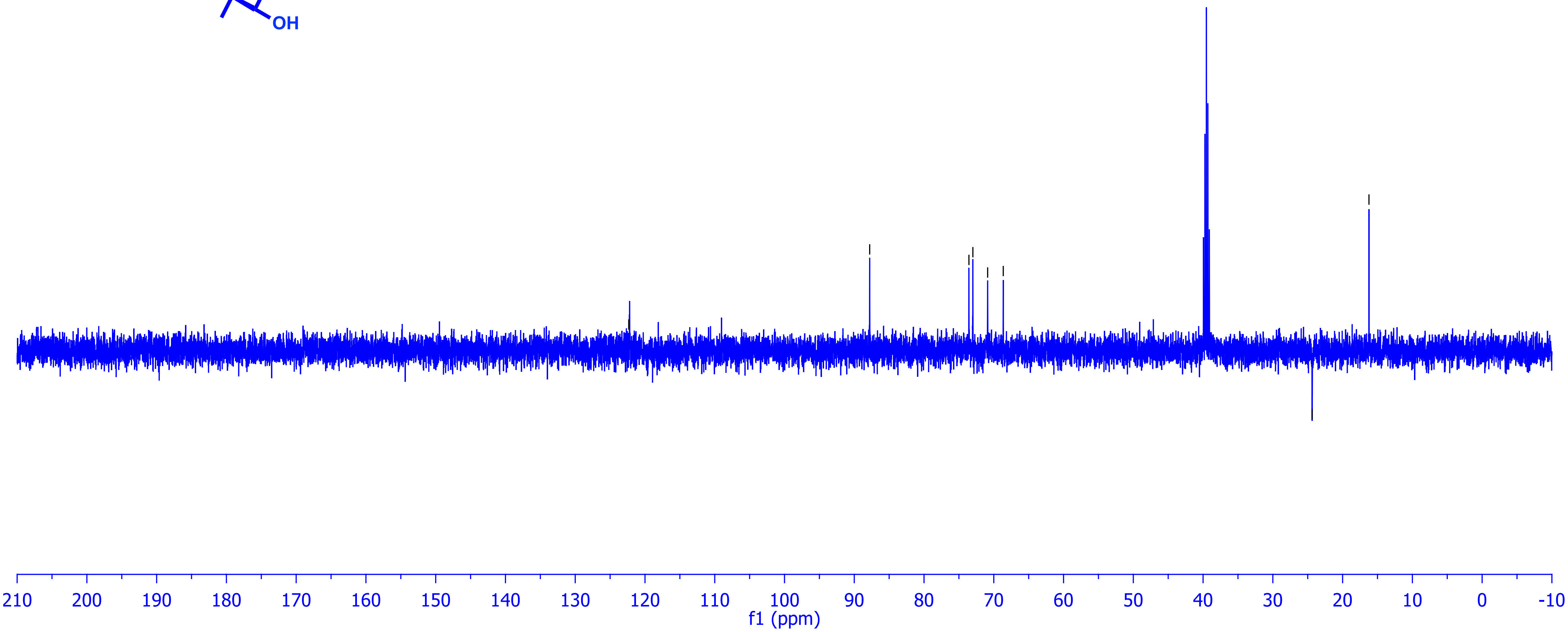

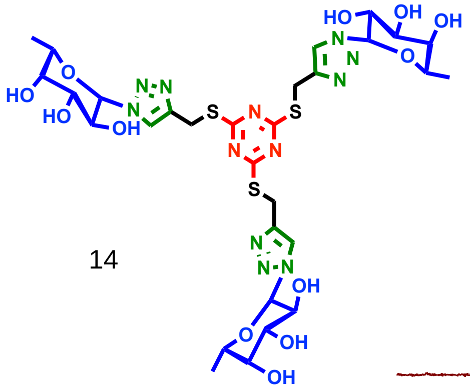
HO
OH
OH
O
N
N
N
HO
HO
OH
S
N
N
N
S
OH
O
OH
OH

14

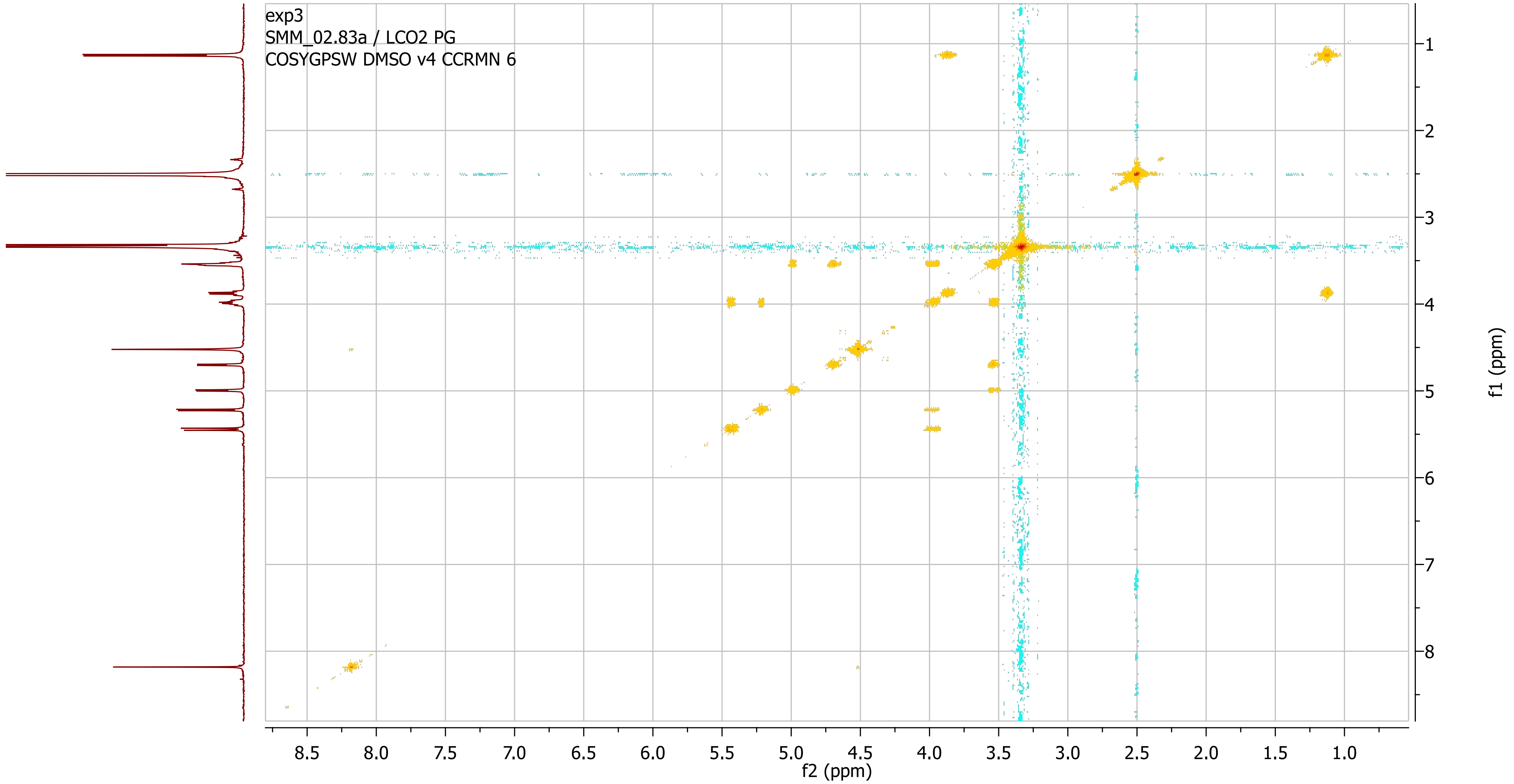
exp3
SMM_02.83a / LCO2 PG
COSYGPSW DMSO v4 CCRMN 6
f2 (ppm)
f1 (ppm)

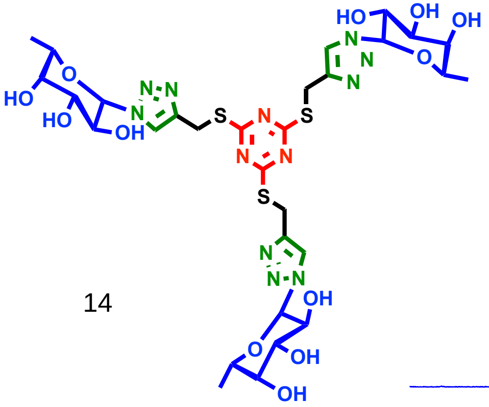

14

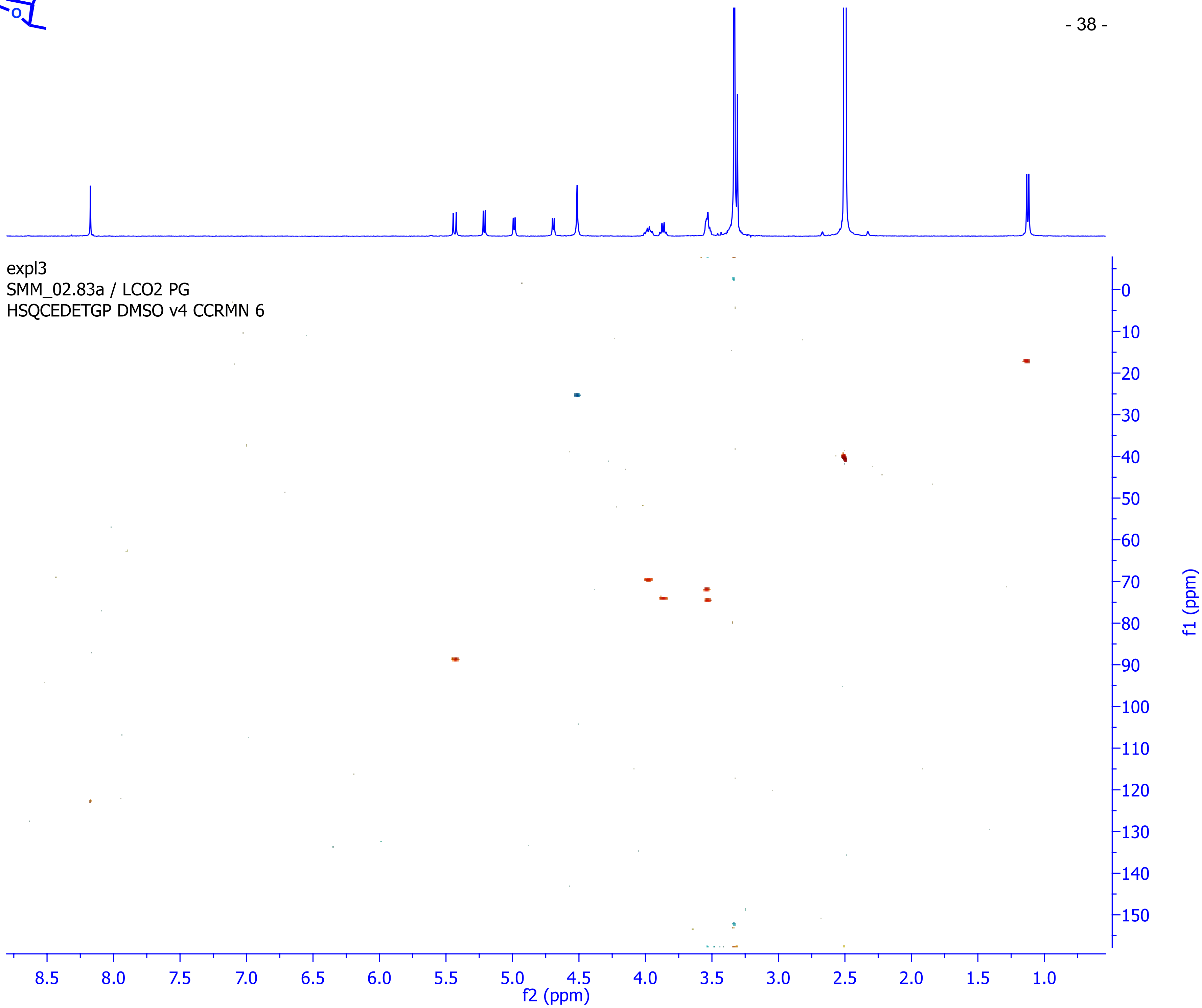
expl3
SMM_02.83a / LCO2 PG
HSQCEDETGP DMSO v4 CCRMN 6
f2 (ppm)
f1 (ppm)

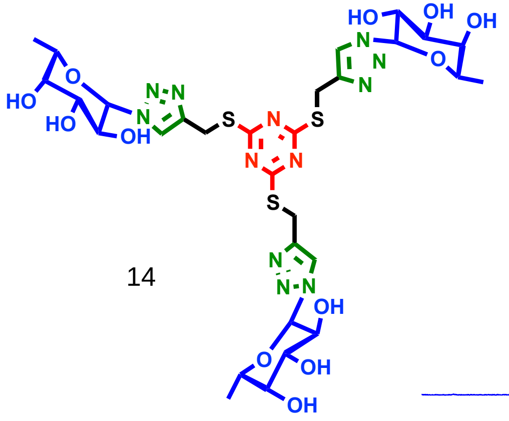

14

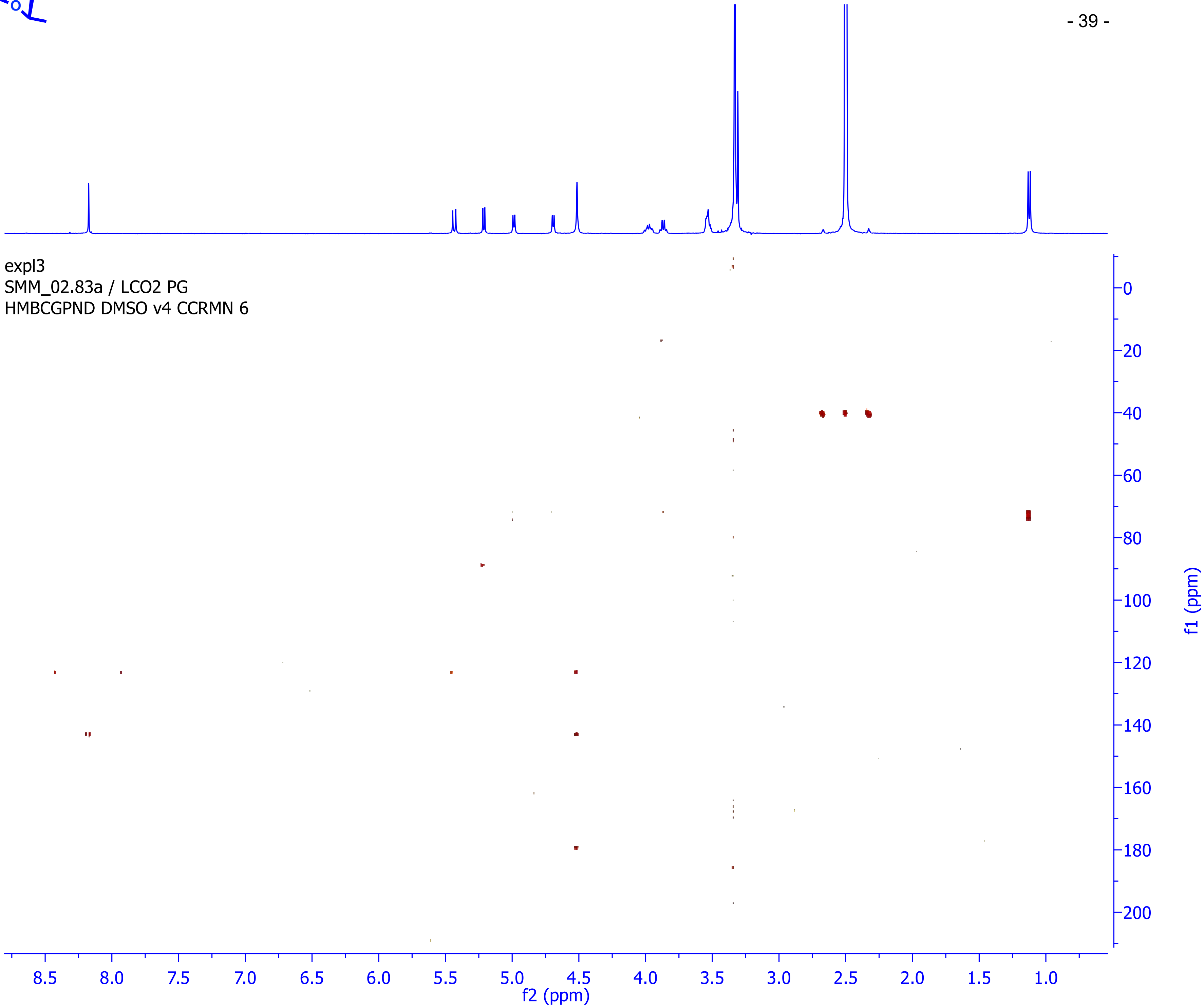

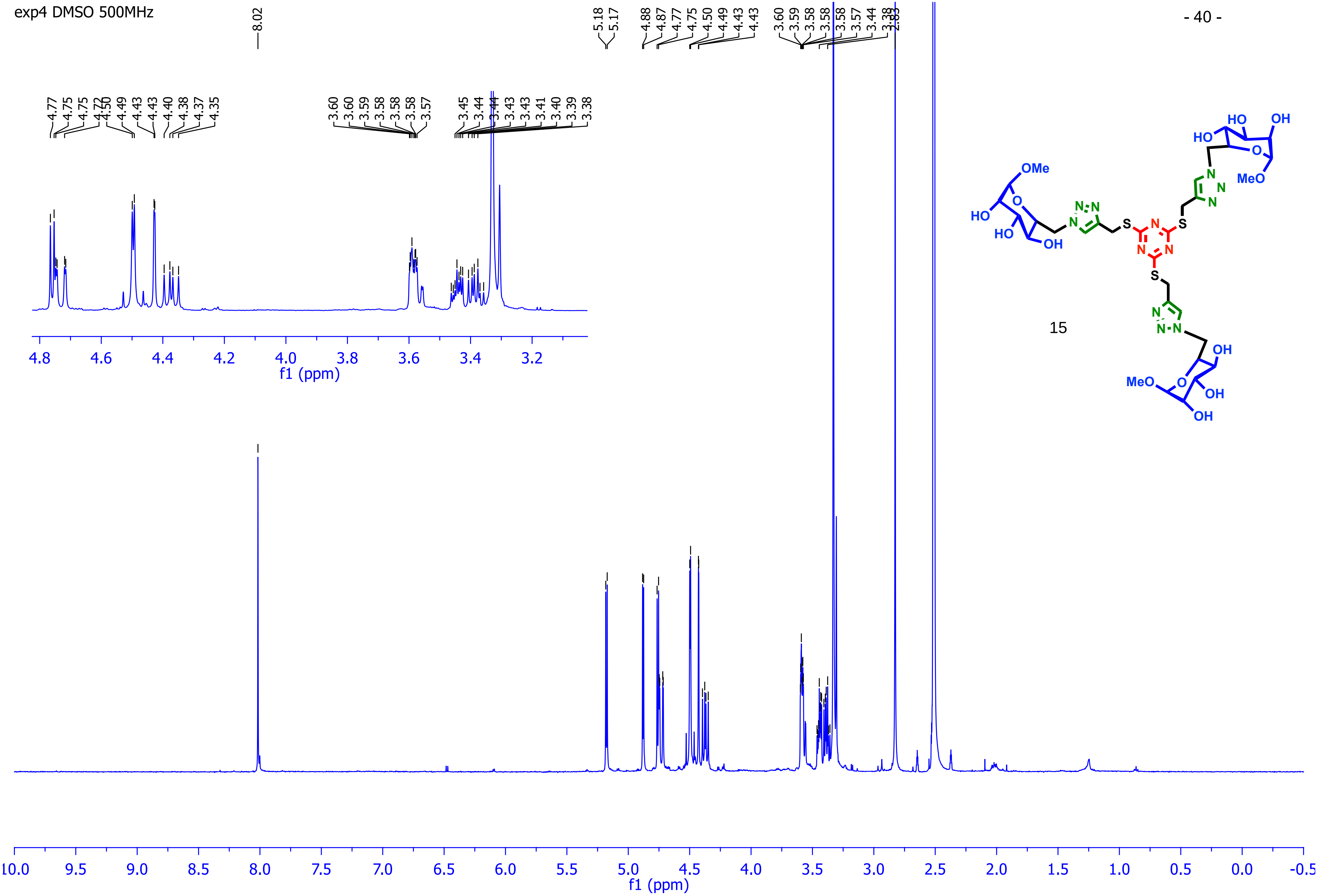
exp4 DMSO 500MHz
8.02
5.18
5.17
4.88
4.87
4.77
4.75
4.50
4.49
4.43
4.43
3.60
3.59
3.58
3.58
3.58
3.57
3.44
3.38
2.83
f1 (ppm)
OMe
HO
HO
OH
MeO
S
N
N
N
15

exp4 DMSO

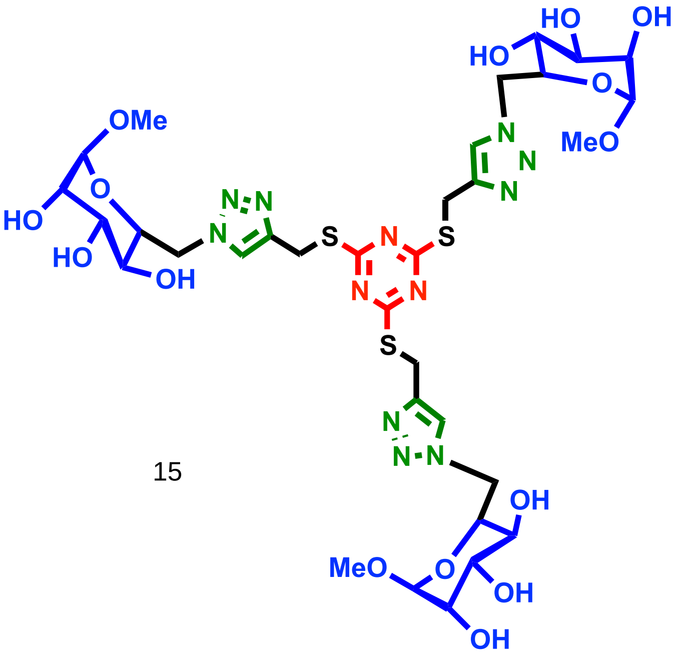
OMe
HO
O
HO
OH
N
N
N
S
N
N
N
S
S
HO
HO
OH
O
MeO
OH
MeO
O
OH
OH

15

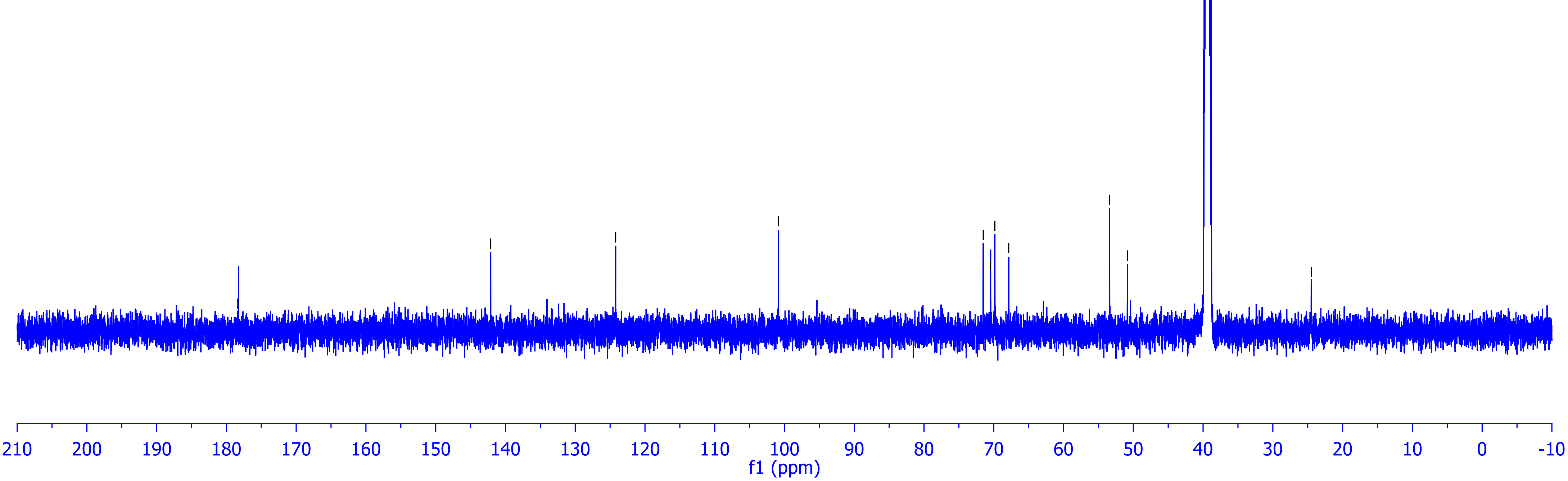
178.36
142.12
124.21
100.88
71.51
70.47
69.84
67.86
53.39
50.84
24.49
210 200 190 180 170 160 150 140 130 120 110 100 90 80 70 60 50 40 30 20 10 0 -10
f1 (ppm)

expl4
13C_dept135sp DMSO /opt/topspin lco2_pg 2

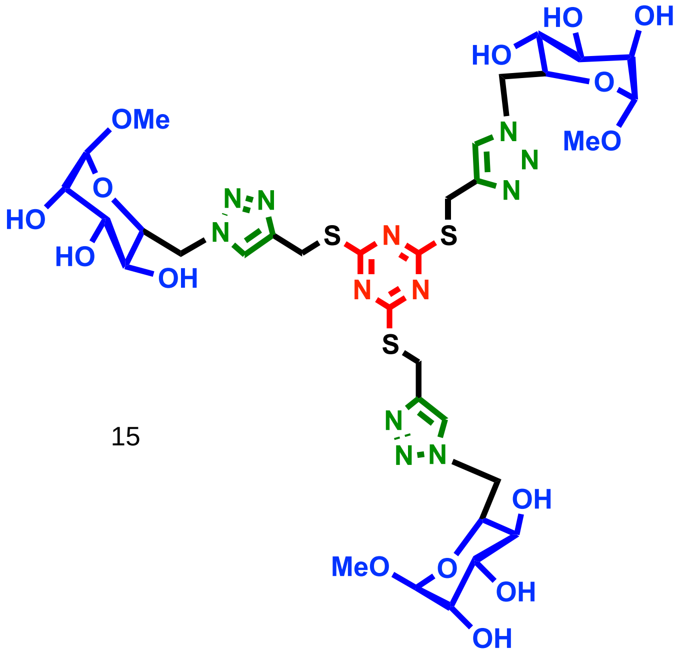

15

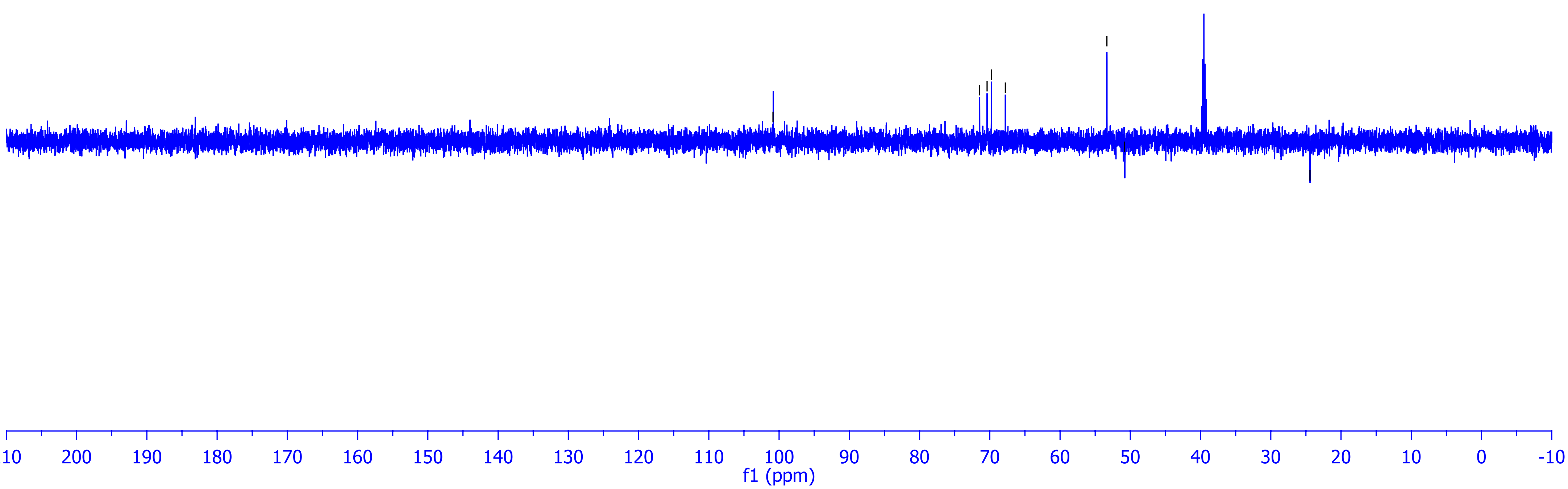

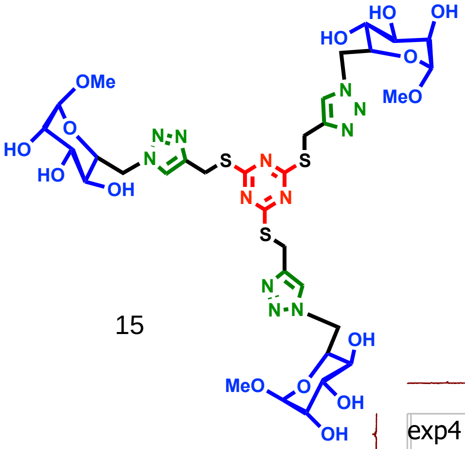
OMe
HO
HO
OH
MeO
OH
MeO
OH
OH

15

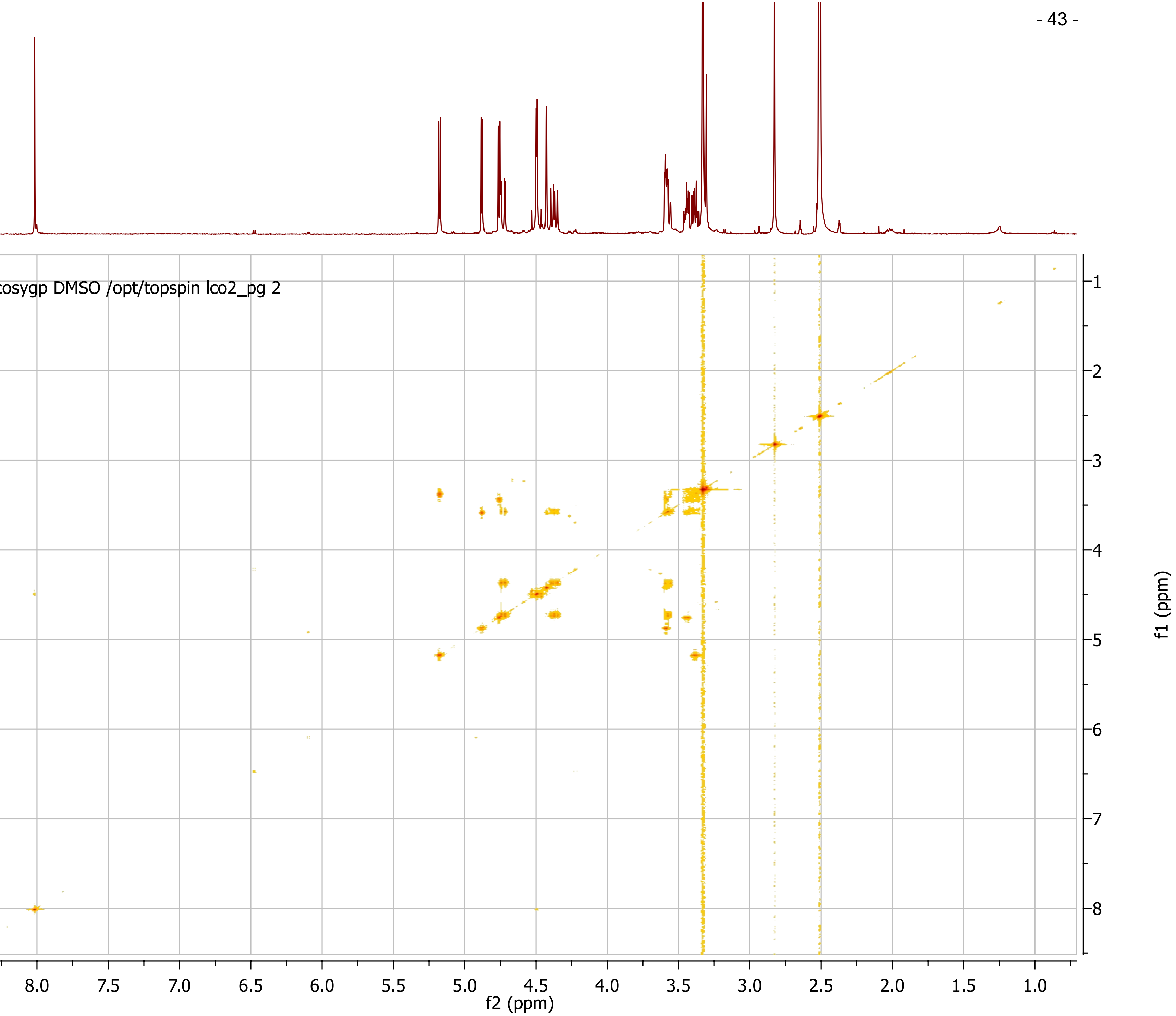
exp4
1H_cosygp DMSO /opt/topspin lco2_pg 2
f2 (ppm)
f1 (ppm)

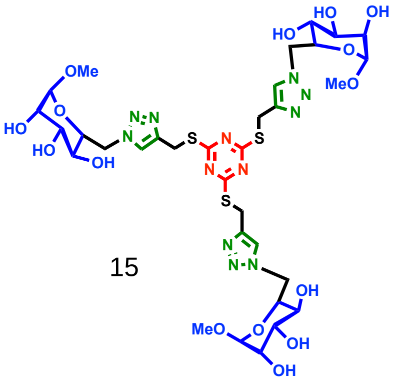

15

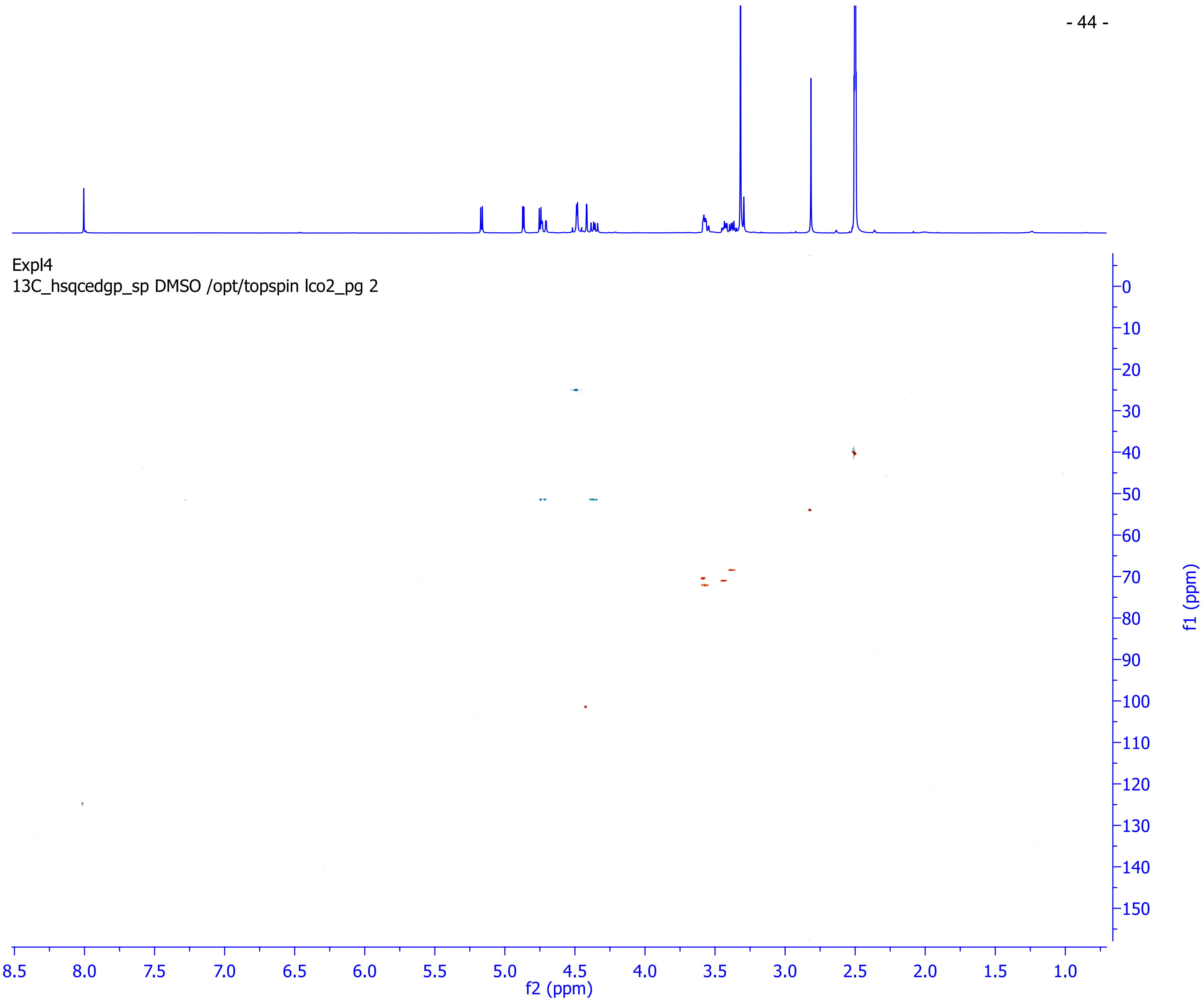

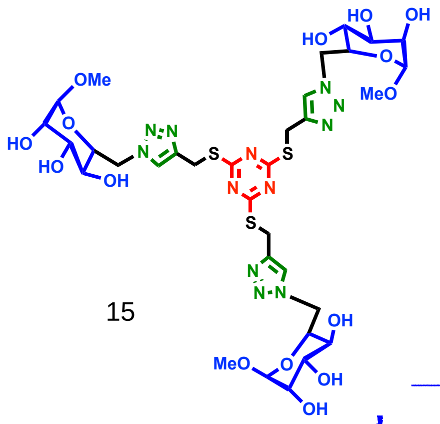
HO
OH
HO
MeO
OMe
HO
HO
OH
S
S
S
N
MeO
OH
OH
OH

15

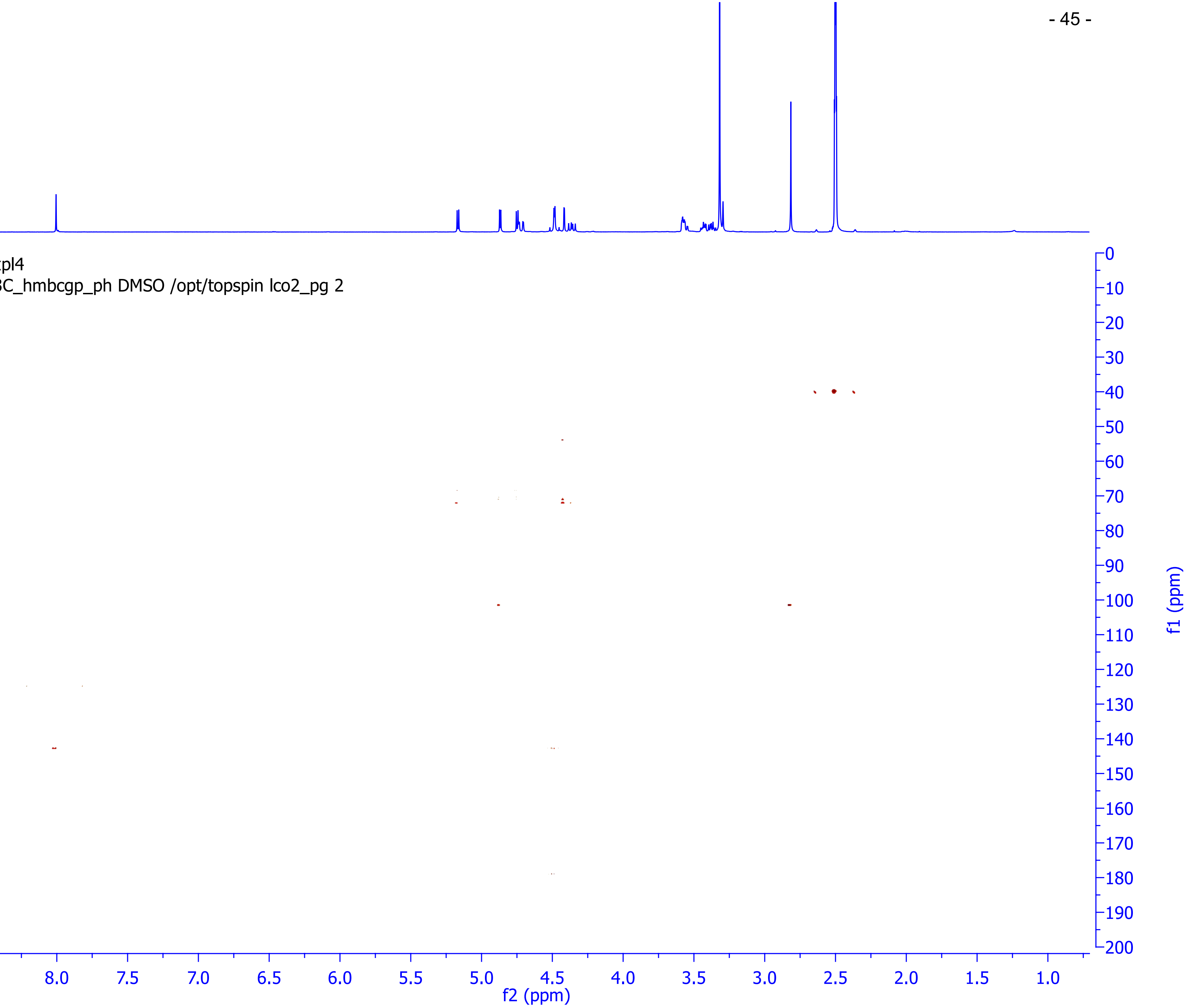
expl4
13C_hmbcgp_ph DMSO /opt/topspin lco2_pg 2
f2 (ppm)
f1 (ppm)

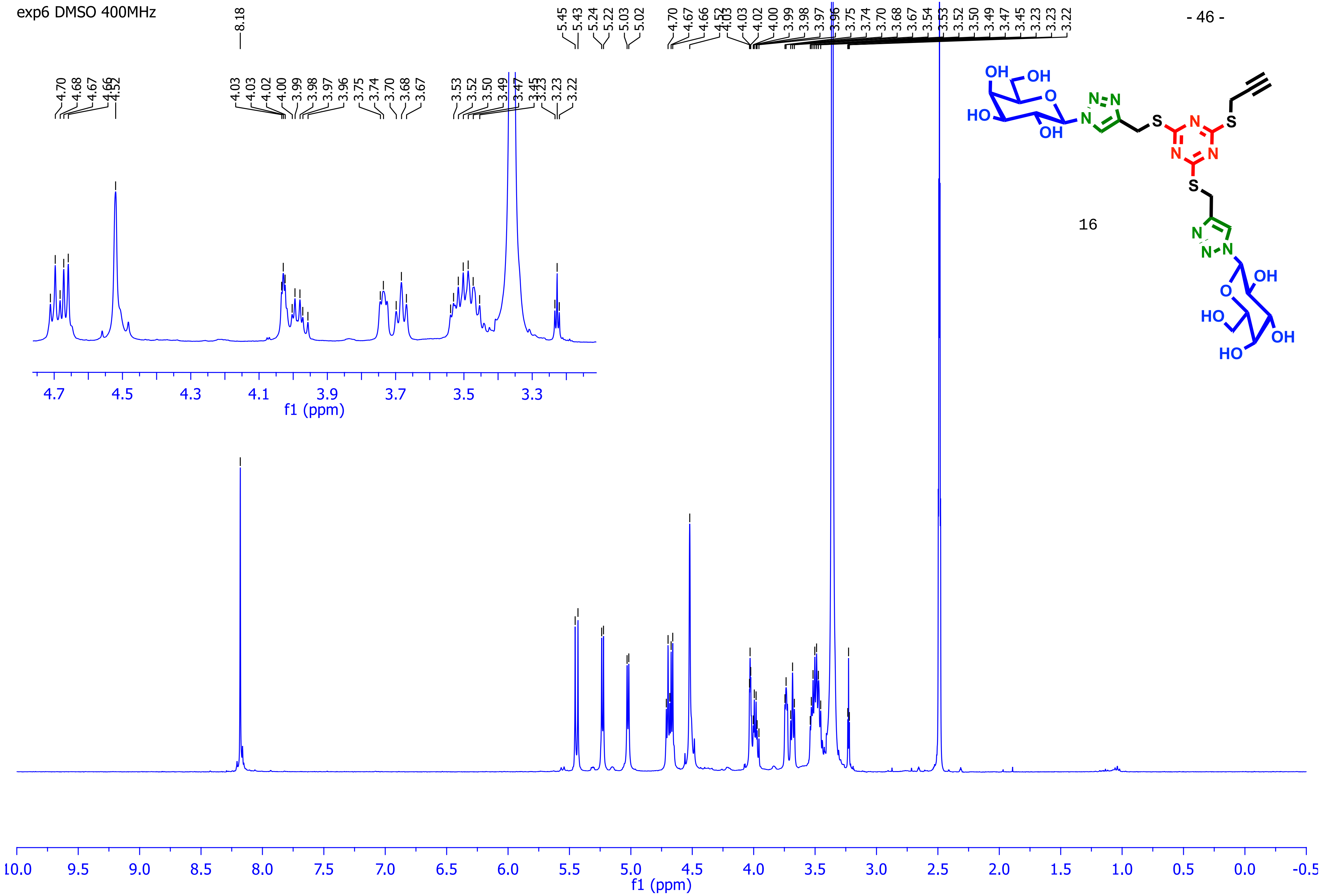
exp6 DMSO 400MHz
8.18
5.45
5.43
5.24
5.22
5.03
5.02
4.70
4.67
4.66
4.52
4.03
4.03
4.02
4.00
3.99
3.98
3.97
3.96
3.75
3.74
3.70
3.68
3.67
3.54
3.53
3.52
3.50
3.49
3.47
3.45
3.23
3.23
3.22
4.70
4.68
4.67
4.66
4.65
4.52
4.03
4.03
4.02
4.00
3.99
3.98
3.97
3.96
3.75
3.74
3.70
3.68
3.67
3.53
3.52
3.50
3.49
3.47
3.45
3.23
3.23
3.22
f1 (ppm)
f1 (ppm)
16

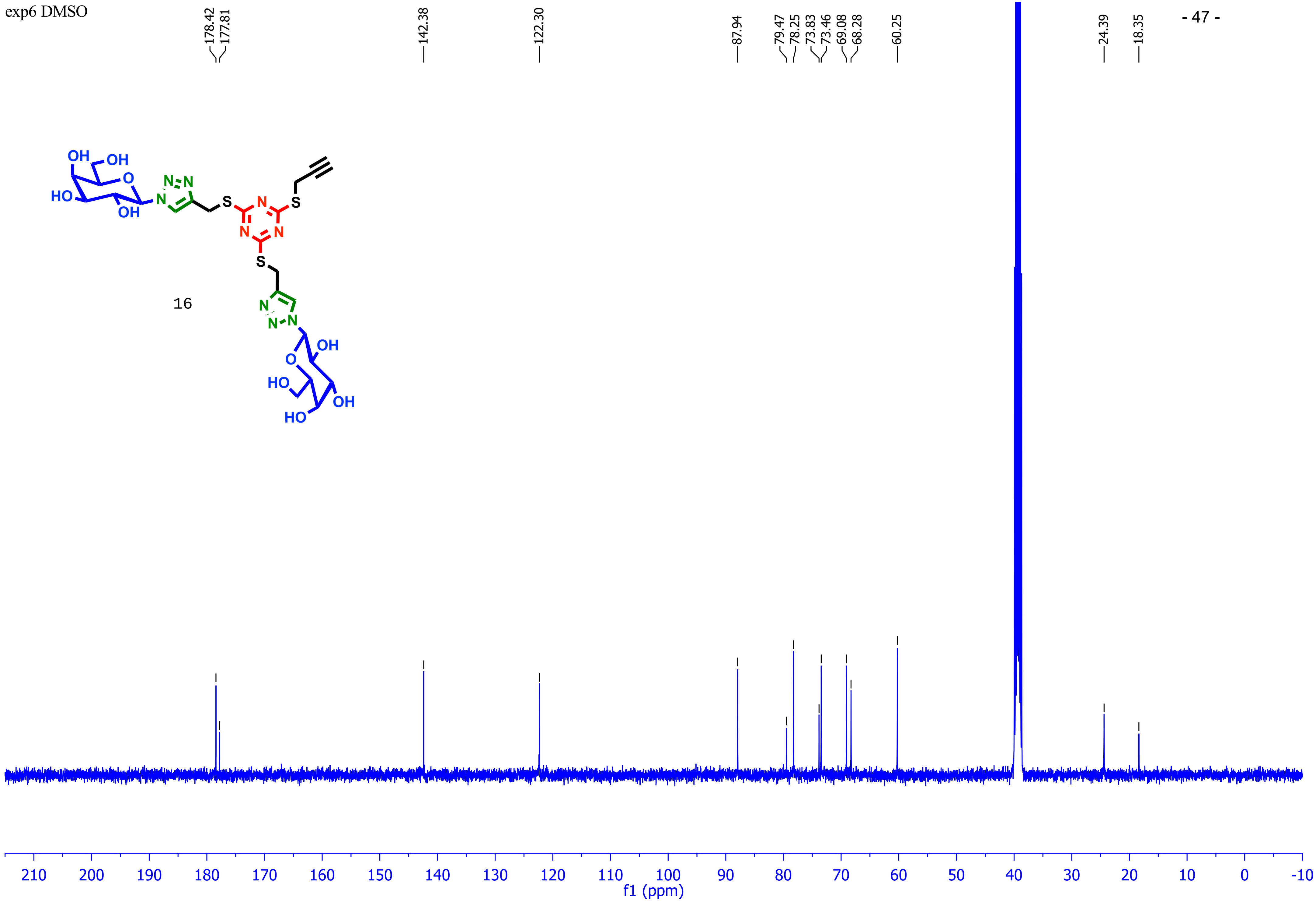
exp6 DMSO
178.42
177.81
142.38
122.30
87.94
79.47
78.25
73.83
73.46
69.08
68.28
60.25
24.39
18.35
16
210 200 190 180 170 160 150 140 130 120 110 100 90 80 70 60 50 40 30 20 10 0 -10
f1 (ppm)

exp6
SMM_0.76d / LCO2 PG
C13DEPT135 DMSO v4 CCRMN 5

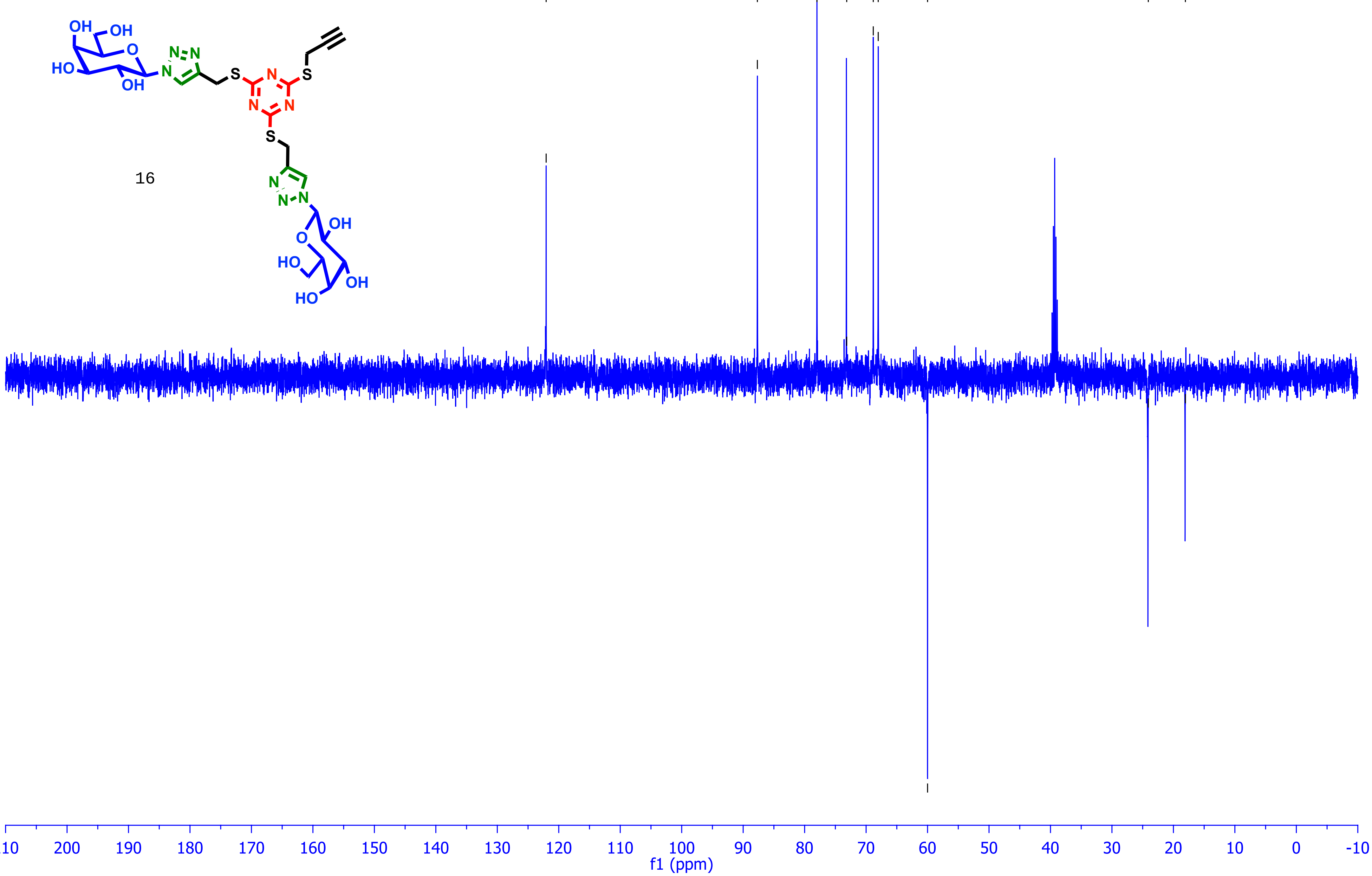

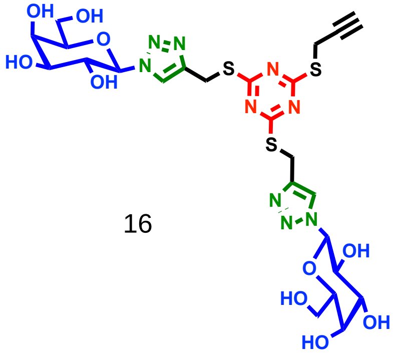
OH
OH
O
HO
OH
N
N
N
S
N
N
N
S
S
N
N
N
O
OH
HO
OH
HO

16

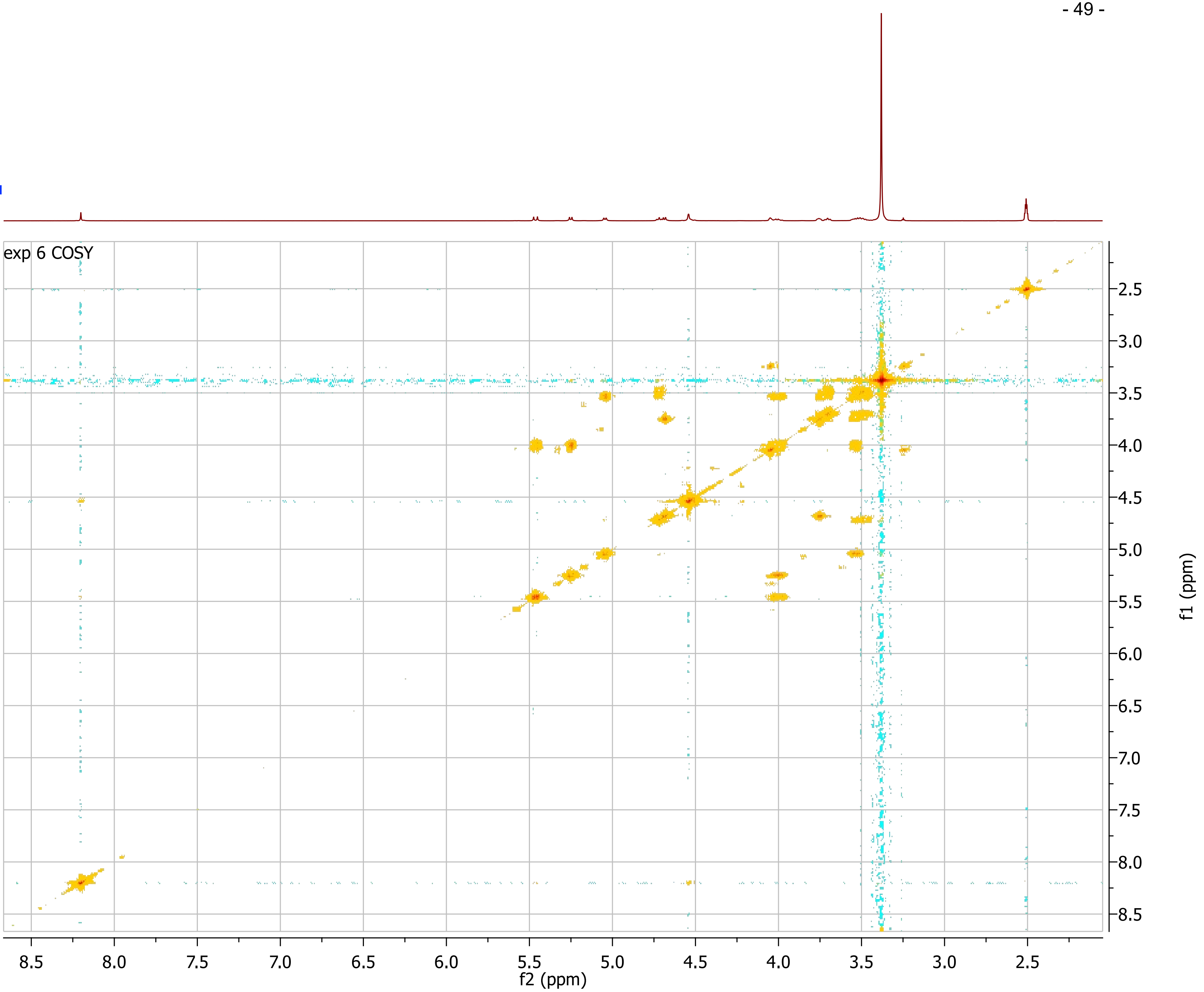
exp 6 COSY
f2 (ppm)
f1 (ppm)
8.5
8.0
7.5
7.0
6.5
6.0
5.5
5.0
4.5
4.0
3.5
3.0
2.5

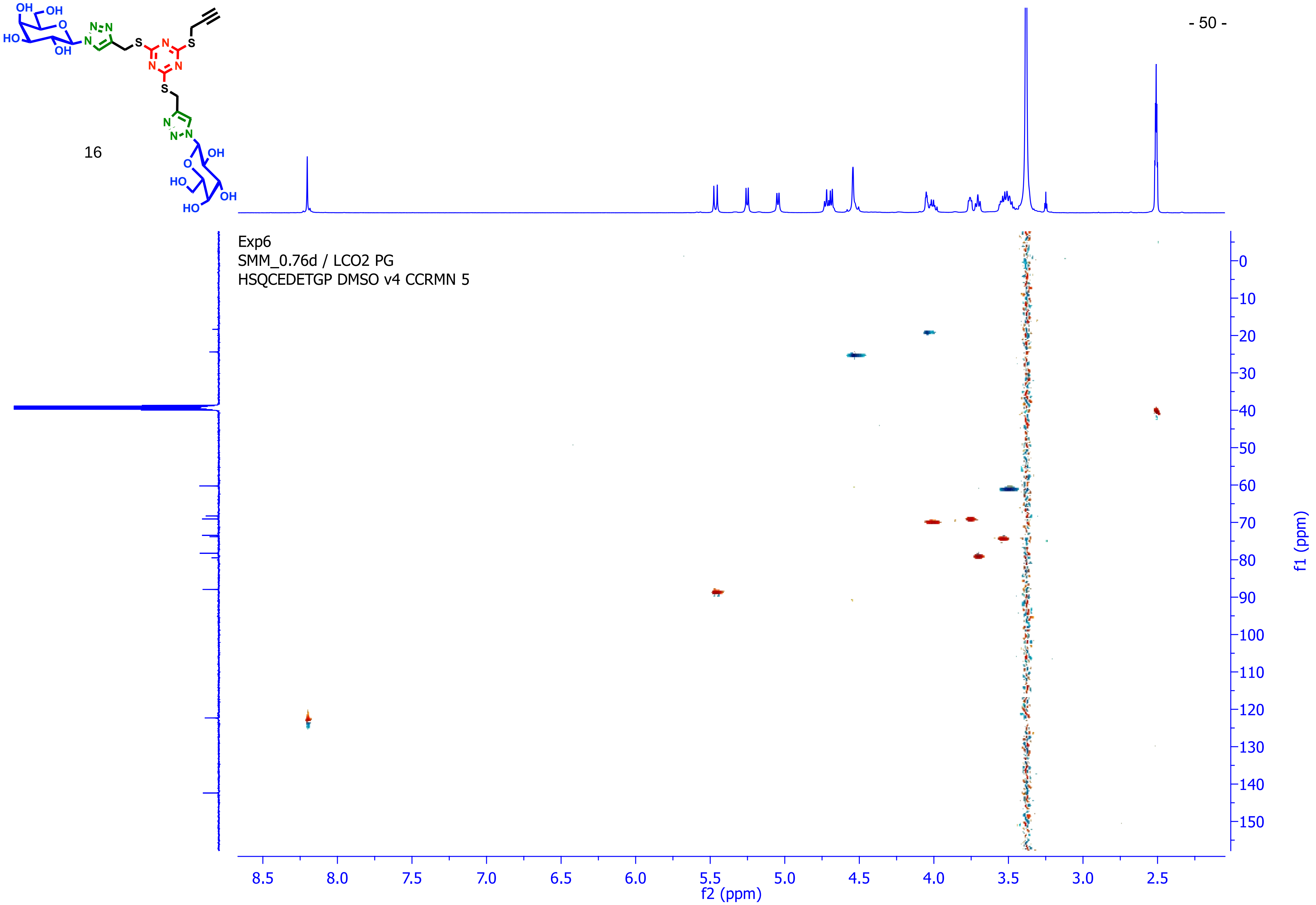
16
Exp6
SMM_0.76d / LCO2 PG
HSQCEDETGP DMSO v4 CCRMN 5
f2 (ppm)
f1 (ppm)

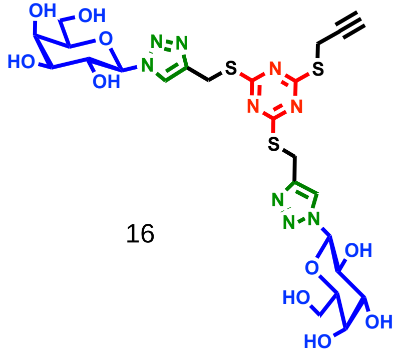
OH
OH
HO
O
OH
N
N
N
S
N
N
N
S
S
N
N
N
O
OH
HO
OH
HO

16

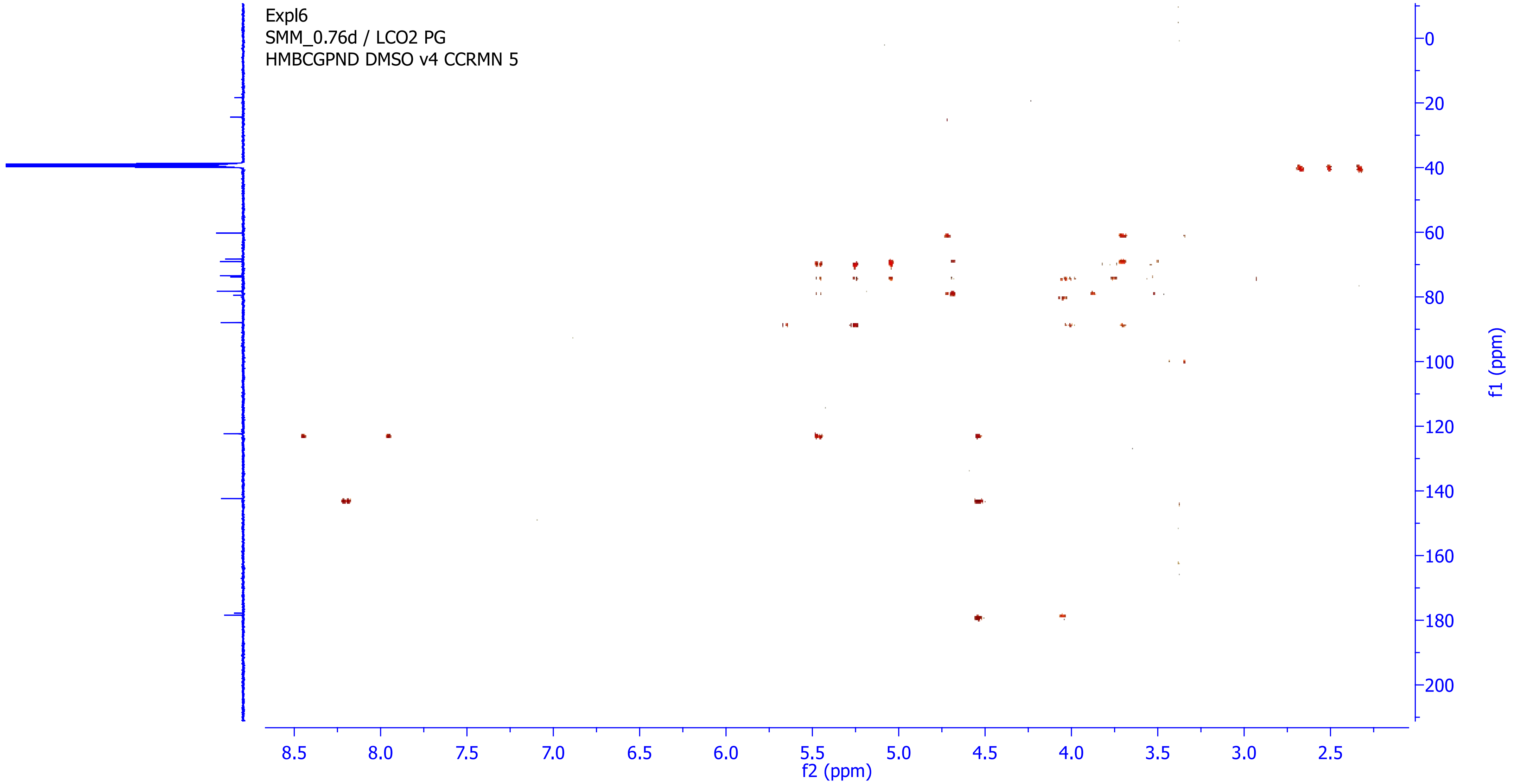
Expl6
SMM_0.76d / LCO2 PG
HMBCGPND DMSO v4 CCRMN 5
f2 (ppm)
f1 (ppm)
8.5
8.0
7.5
7.0
6.5
6.0
5.5
5.0
4.5
4.0
3.5
3.0
2.5
0
20
40
60
80
100
120
140
160
180
200

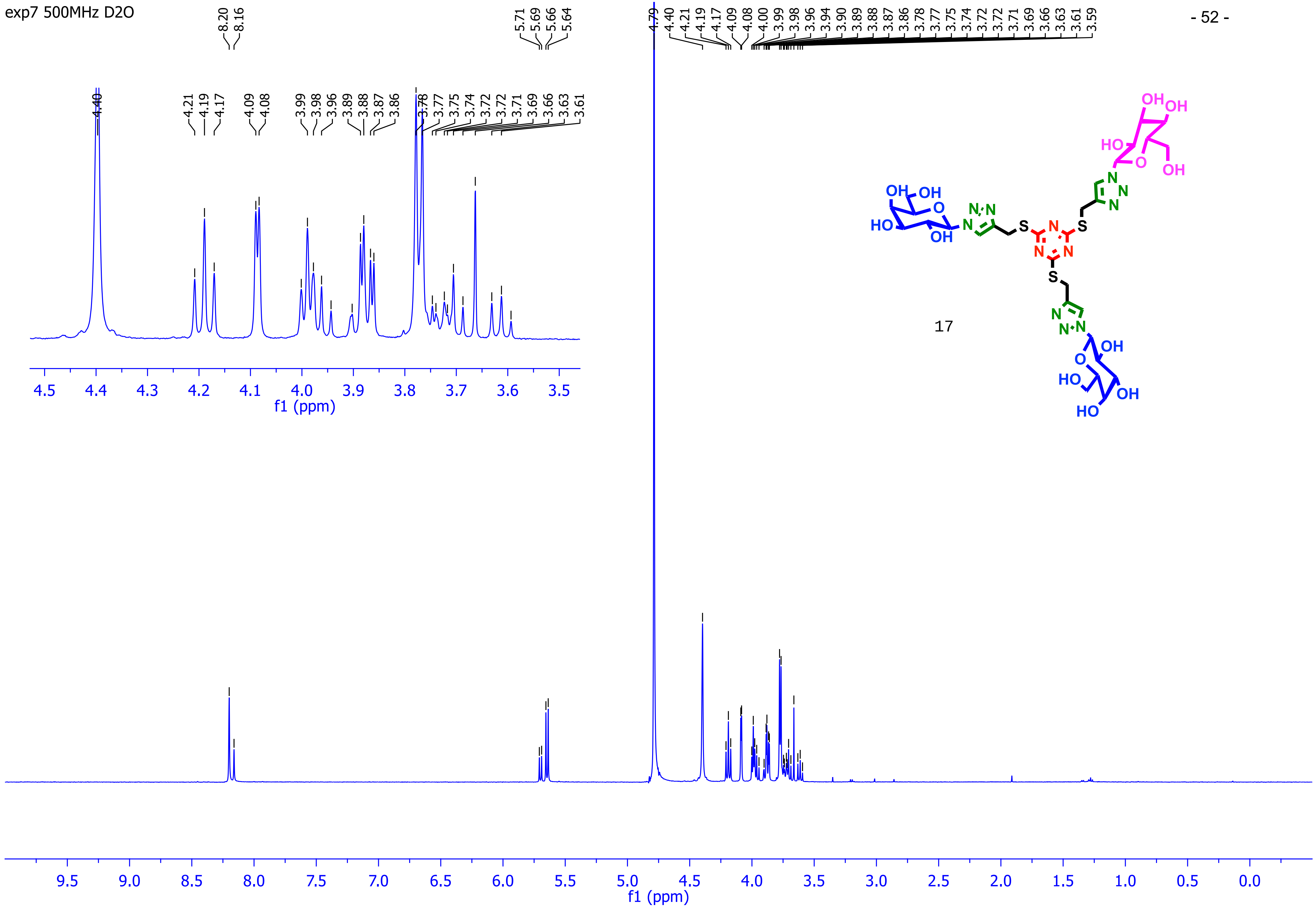
exp7 500MHz D2O
8.20
8.16
5.71
5.69
5.66
5.64
4.79
4.40
4.21
4.19
4.17
4.09
4.08
4.00
3.99
3.98
3.96
3.94
3.90
3.89
3.88
3.87
3.86
3.78
3.77
3.75
3.74
3.72
3.72
3.71
3.69
3.66
3.63
3.61
3.59
f1 (ppm)
9.5 9.0 8.5 8.0 7.5 7.0 6.5 6.0 5.5 5.0 4.5 4.0 3.5 3.0 2.5 2.0 1.5 1.0 0.5 0.0
4.5 4.4 4.3 4.2 4.1 4.0 3.9 3.8 3.7 3.6 3.5
17

expl7
SMM-1-75A lco2PG M. Smadhi 2.2mg/D2O
13C_BB D2O /opt/topspin nmr 3

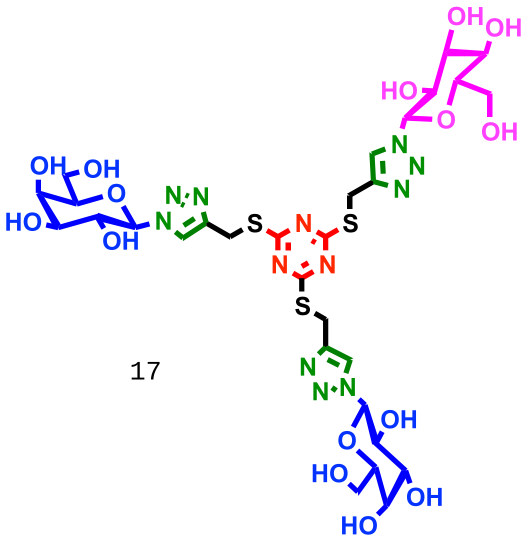

17

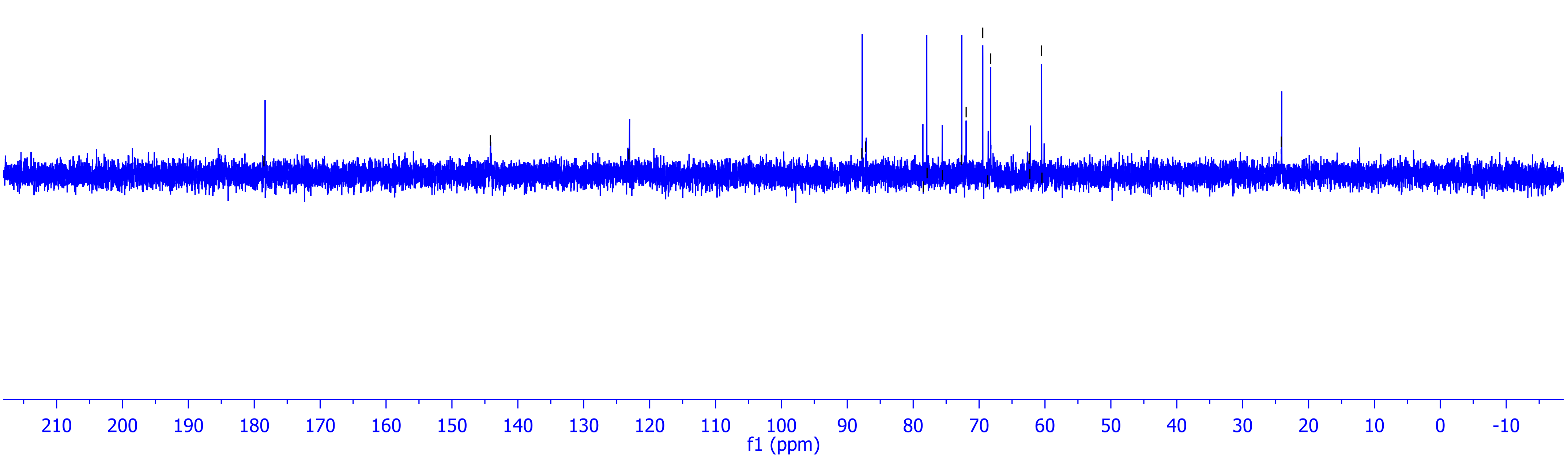

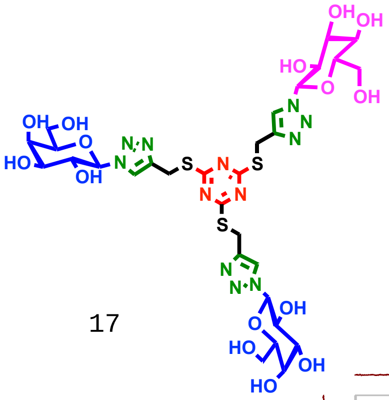
OH
OH
HO
O
OH
OH
OH
HO
O
OH
N
N
N
N
N
N
S
S
N
N
N
S
N
N
N
OH
O
HO
OH
HO

17

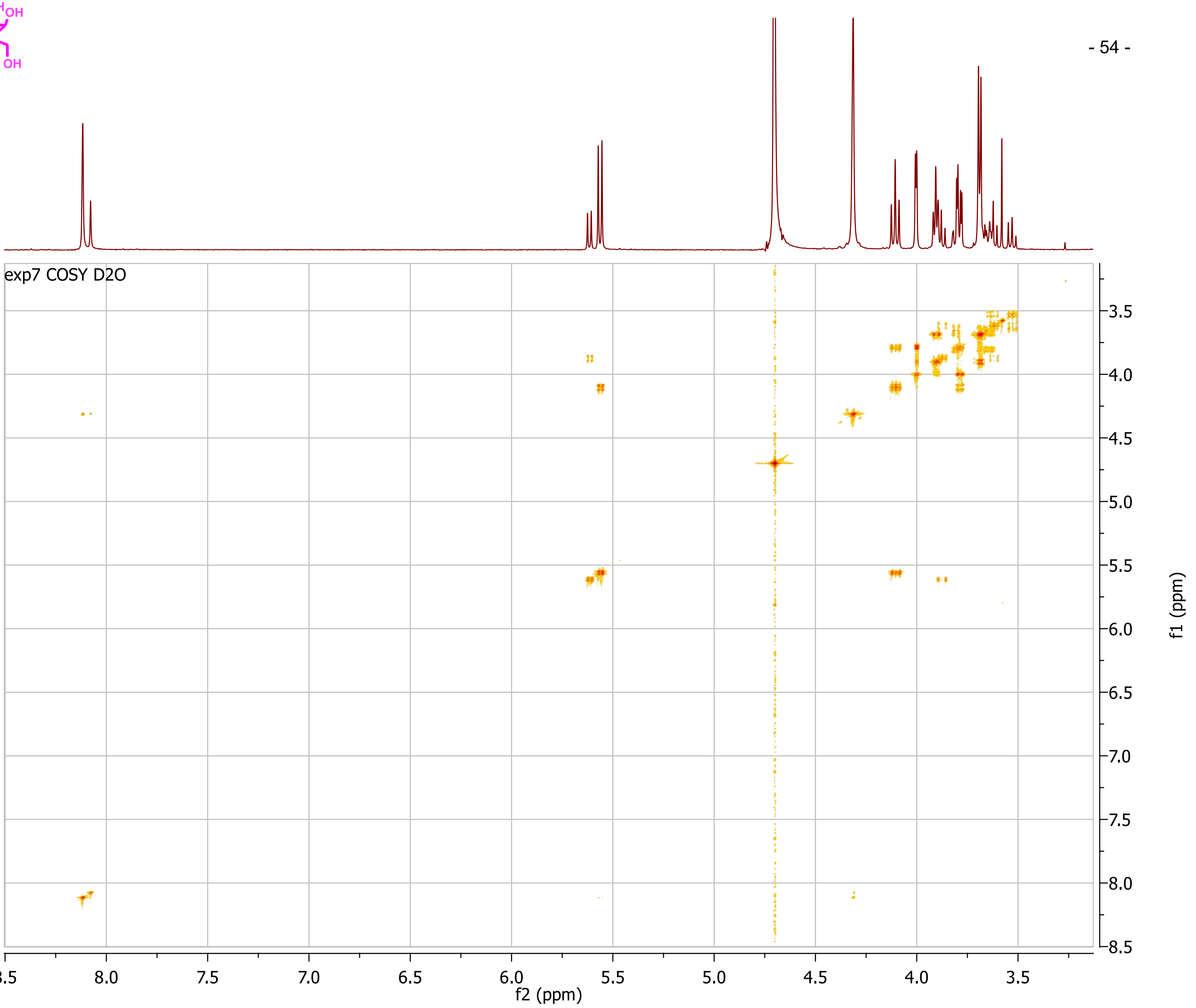
exp7 COSY D2O
f2 (ppm)
f1 (ppm)
8.5
8.0
7.5
7.0
6.5
6.0
5.5
5.0
4.5
4.0
3.5

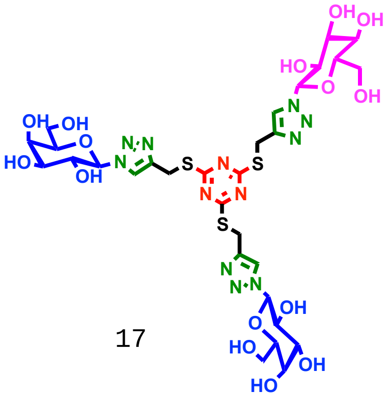

17

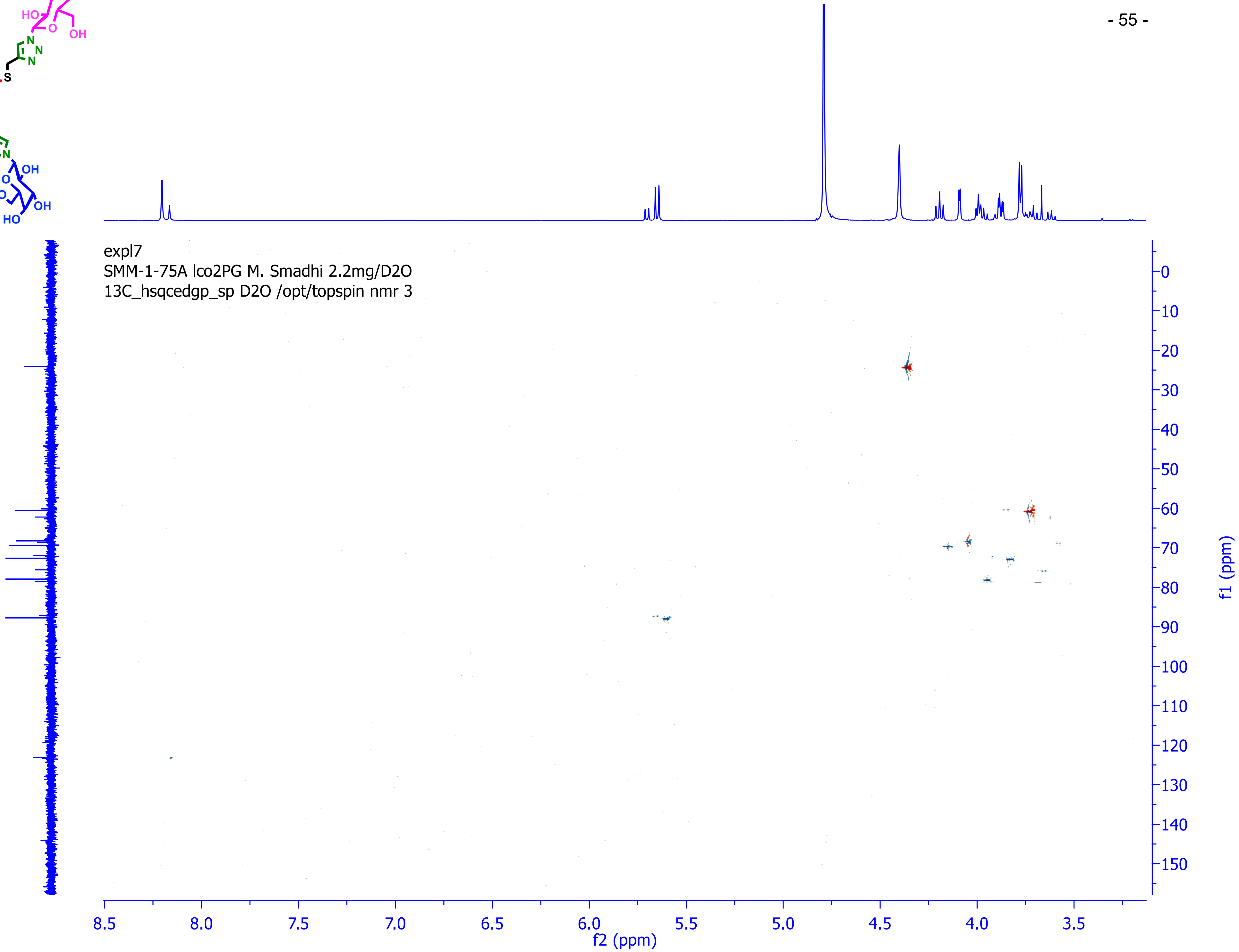
expl7
SMM-1-75A lco2PG M. Smadhi 2.2mg/D2O
13C_hsqcedgp_sp D2O /opt/topspin nmr 3
f2 (ppm)
f1 (ppm)

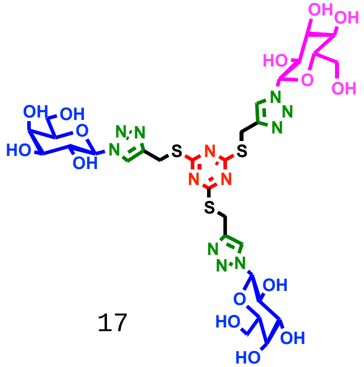
OH
OH
HO
O
OH
N
N
N
OH
OH
O
HO
OH
N
N
N
S
N
N
N
S
S
N
N
N
OH
O
HO
OH
HO

17

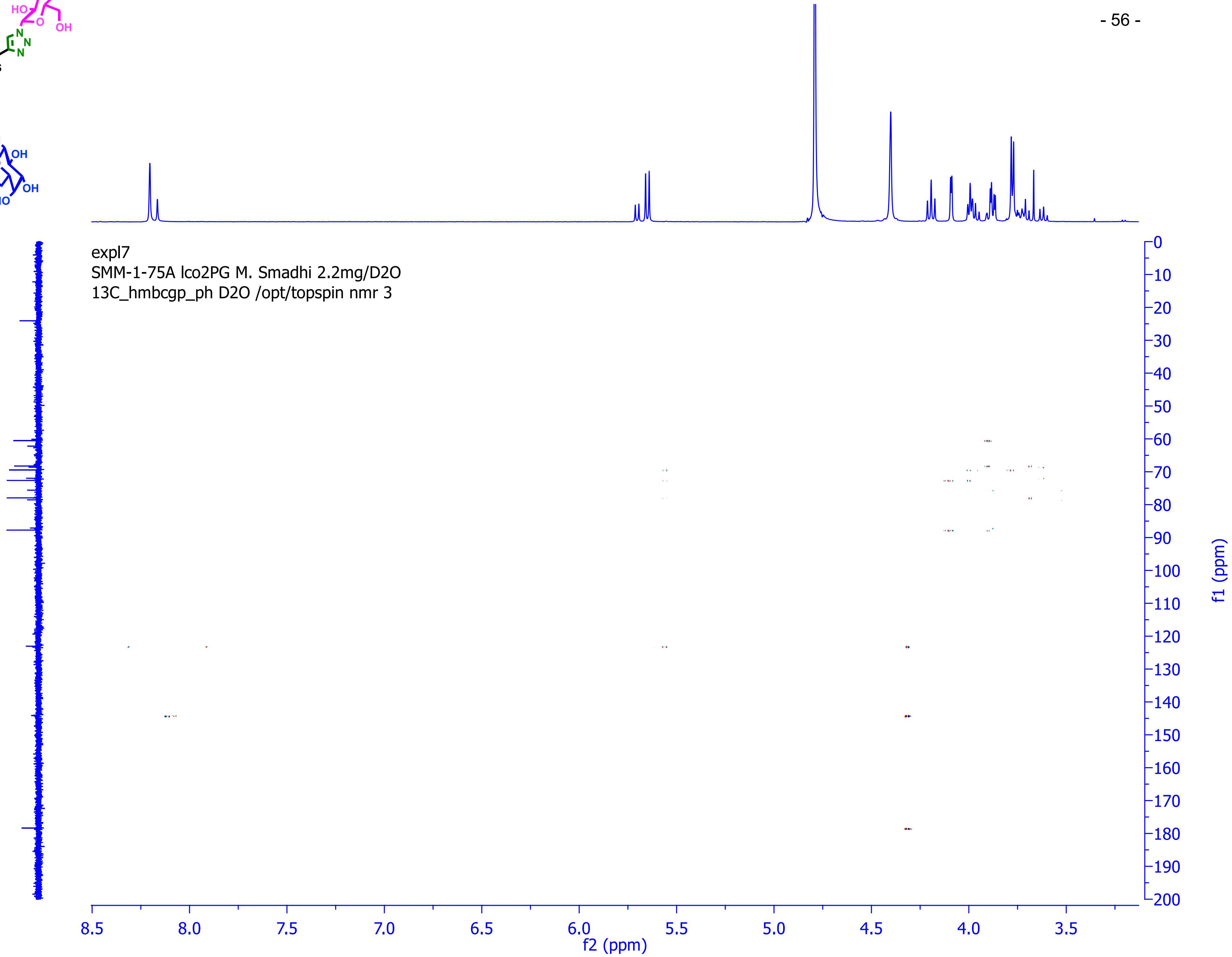
expl7
SMM-1-75A lco2PG M. Smadhi 2.2mg/D2O
13C_hmbcgp_ph D2O /opt/topspin nmr 3
f2 (ppm)
f1 (ppm)
8.5
8.0
7.5
7.0
6.5
6.0
5.5
5.0
4.5
4.0
3.5
0
10
20
30
40
50
60
70
80
90
100
110
120
130
140
150
160
170
180
190
200

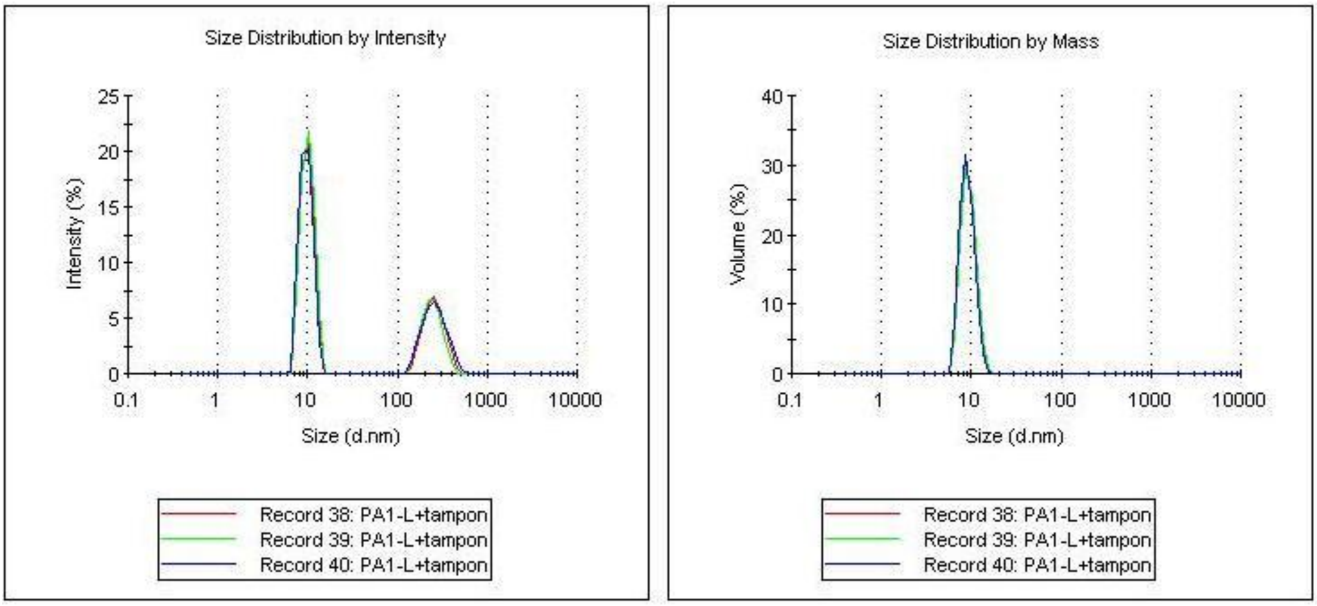

Dynamic light scattering experiments of lecA + buffer. Distribution by intensity (right) and by mass (left) at 3 minute intervals.

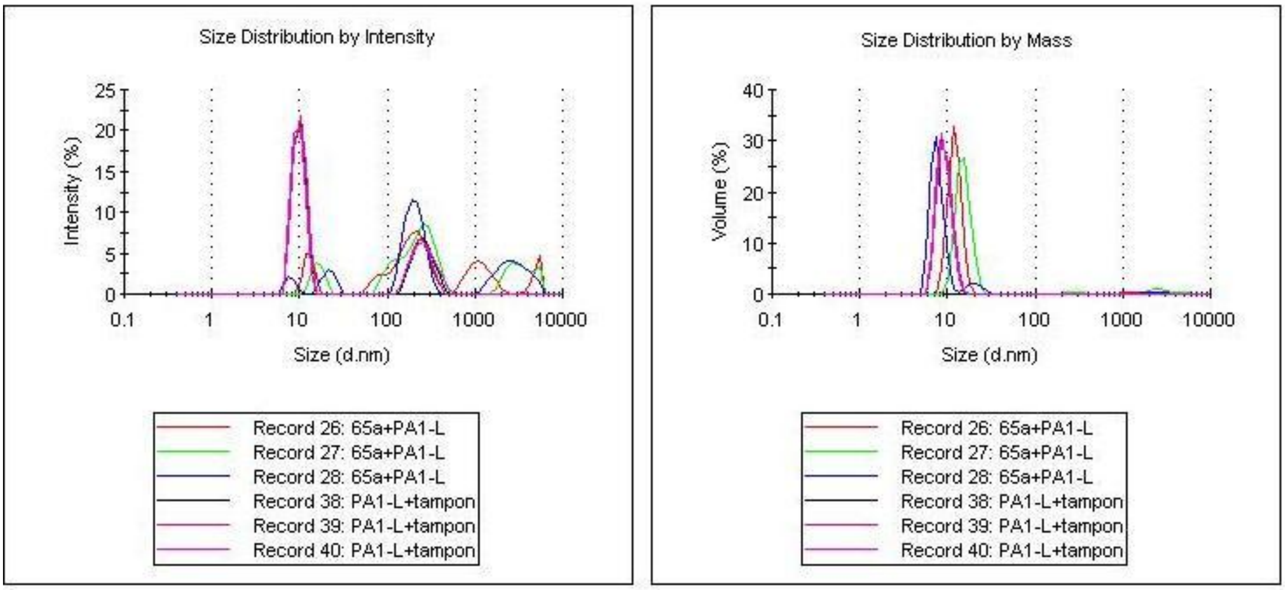

Dynamic light scattering experiments of lecA + glycocluster 1 (200 micromolar) Distribution by intensity (right) and by mass (left) at 3 minute intervals.

Cpd 13

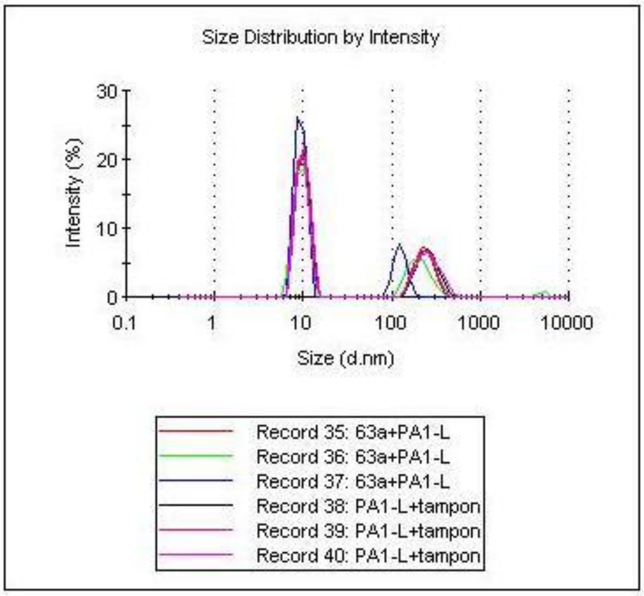

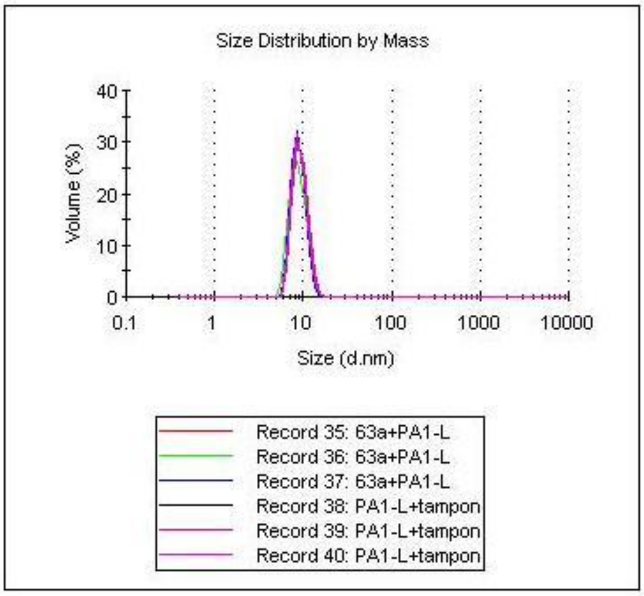

Cpd 17

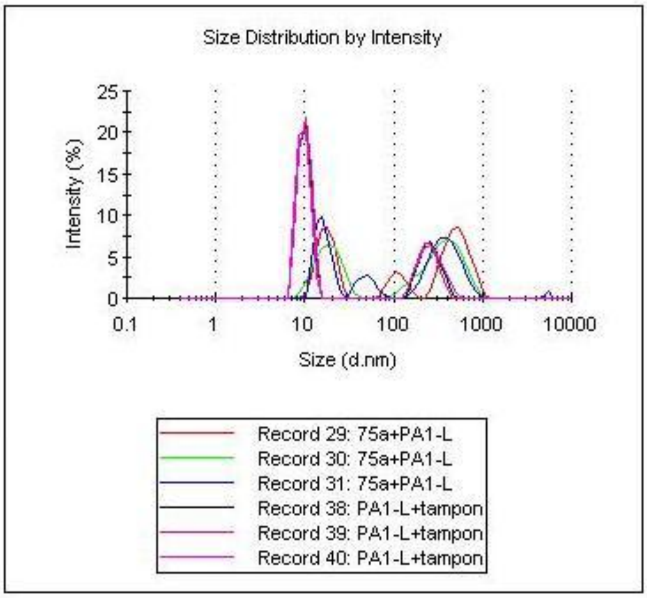

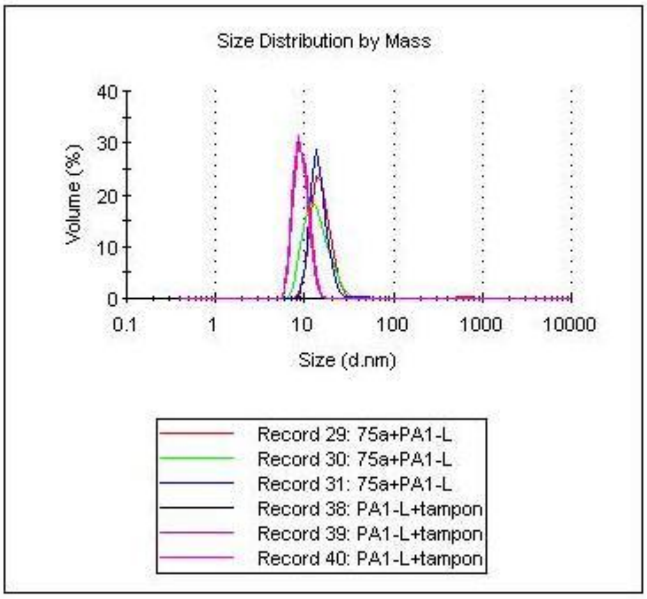

Cpd 16

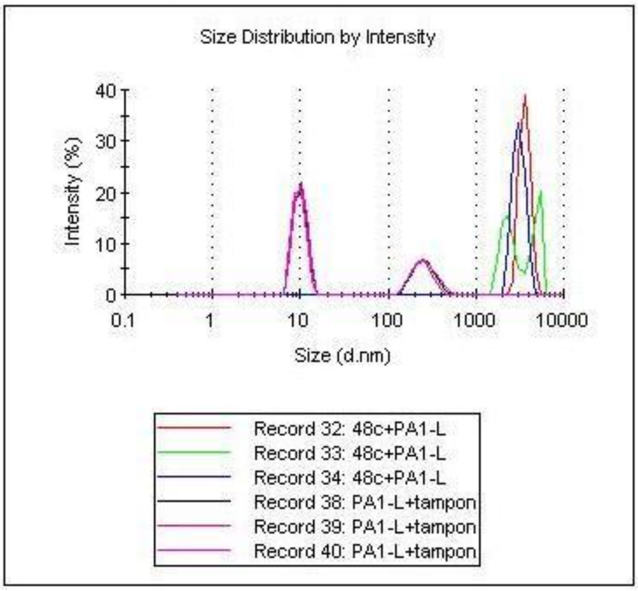

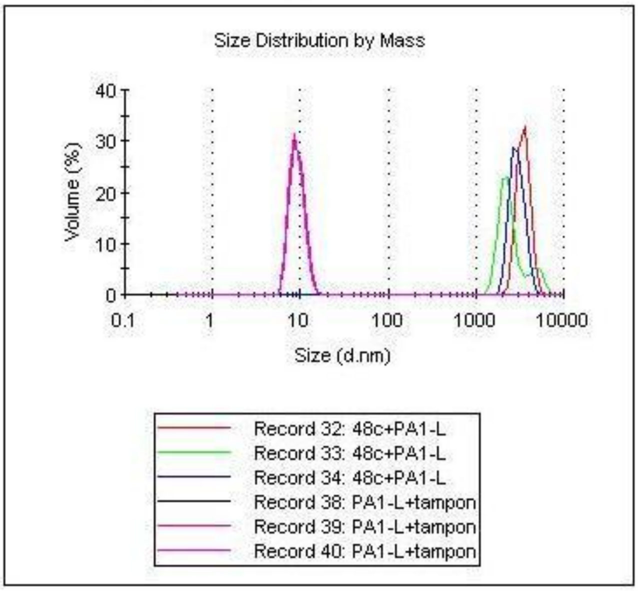

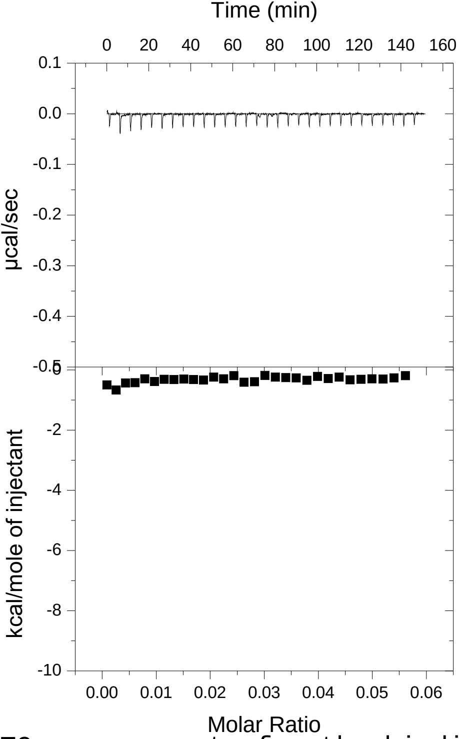

ITC measurements for the binding to lecA of glycocluster 13.

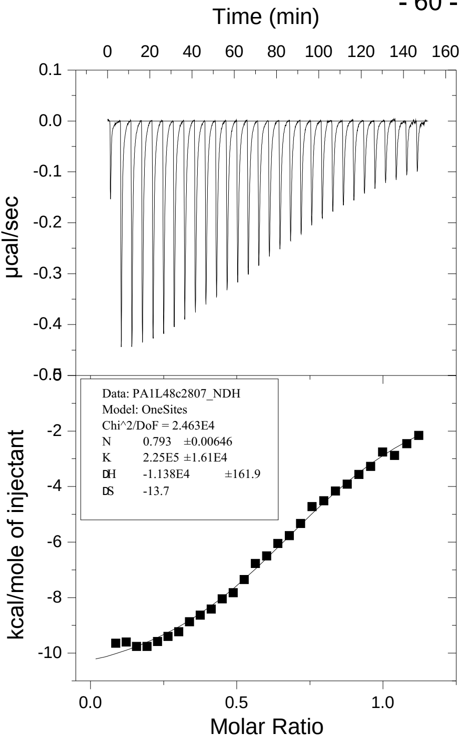

ITC measurements for the binding to lecA of glycocluster 16.

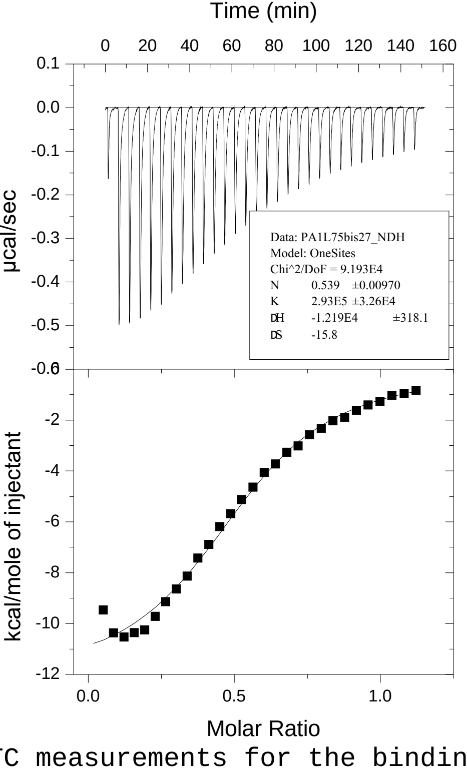

ITC measurements for the binding to lecA of glycocluster 17.

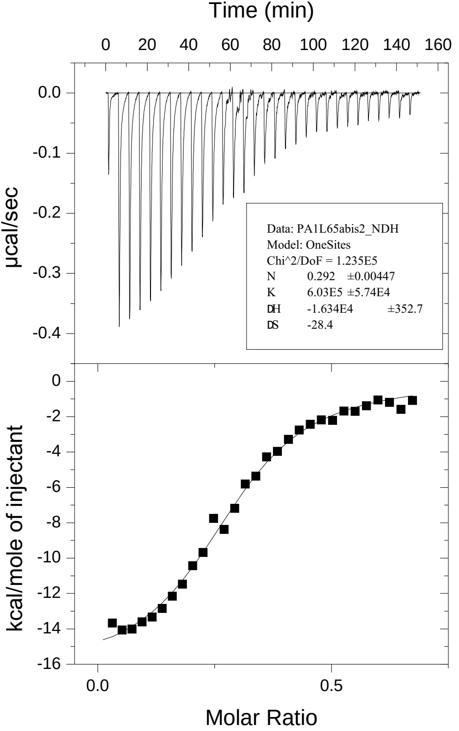

ITC measurement for the binding to lecA of cluster 1 (run 2).

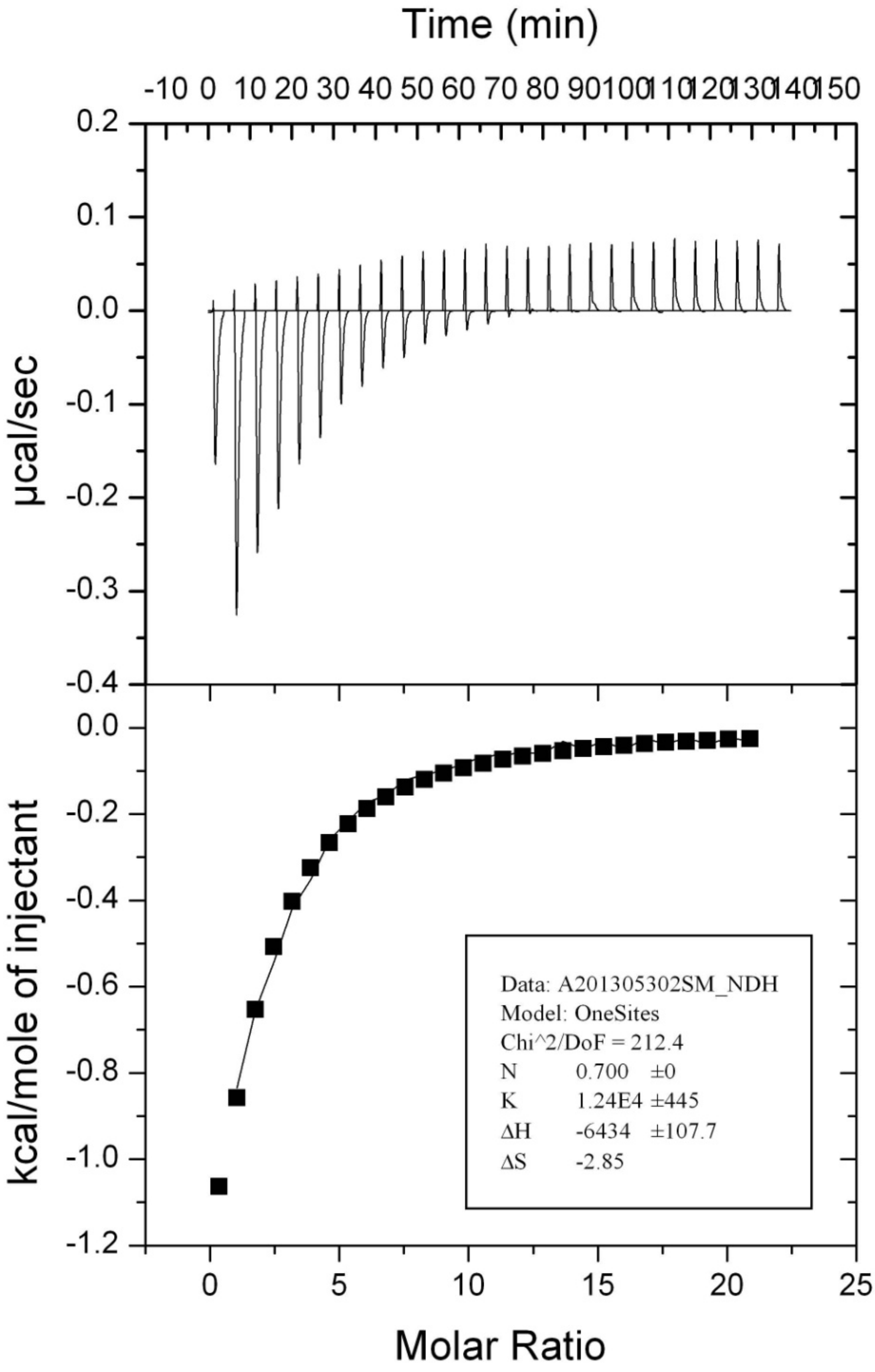

ITC measurements for the binding to lecB of fucose glycocluster 14. (excess lectin)

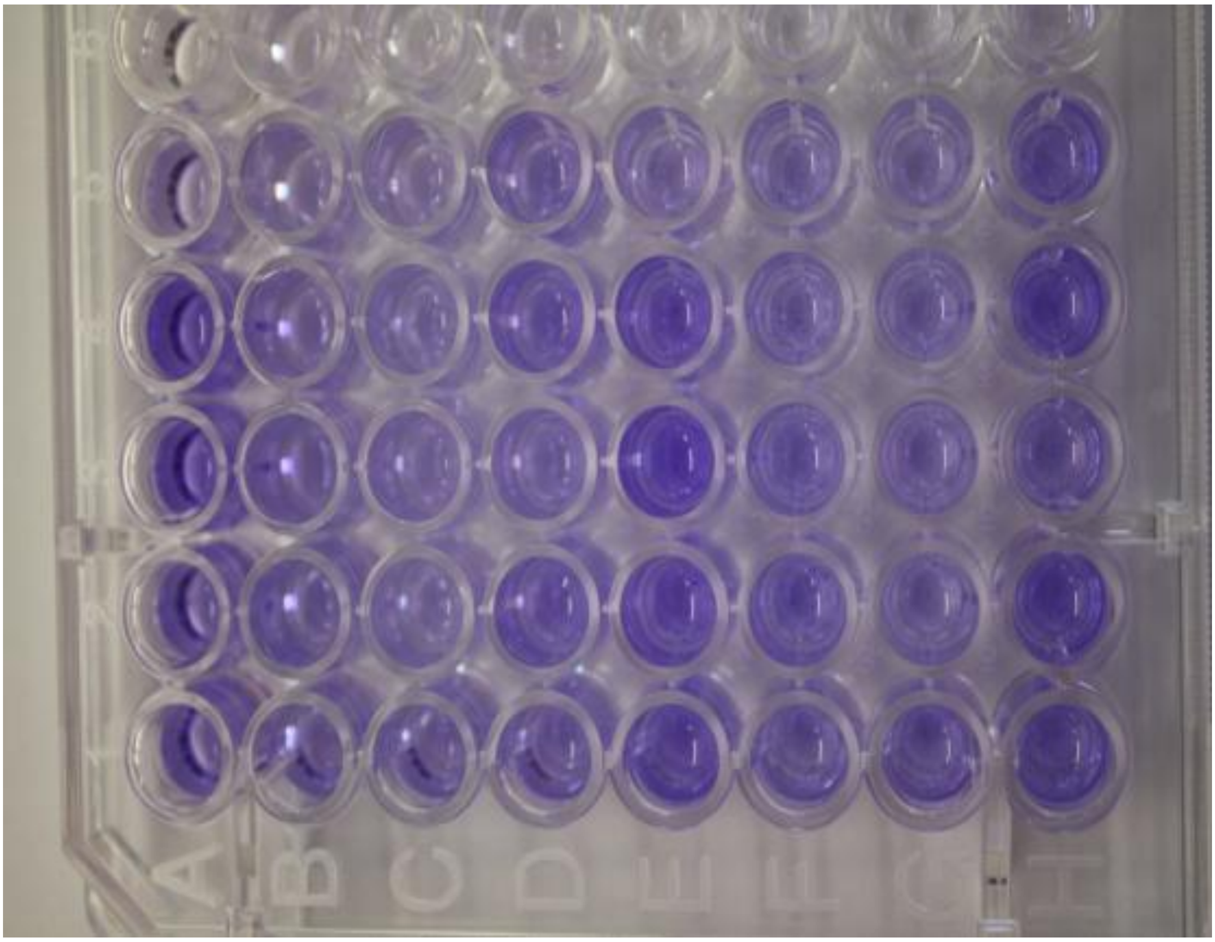

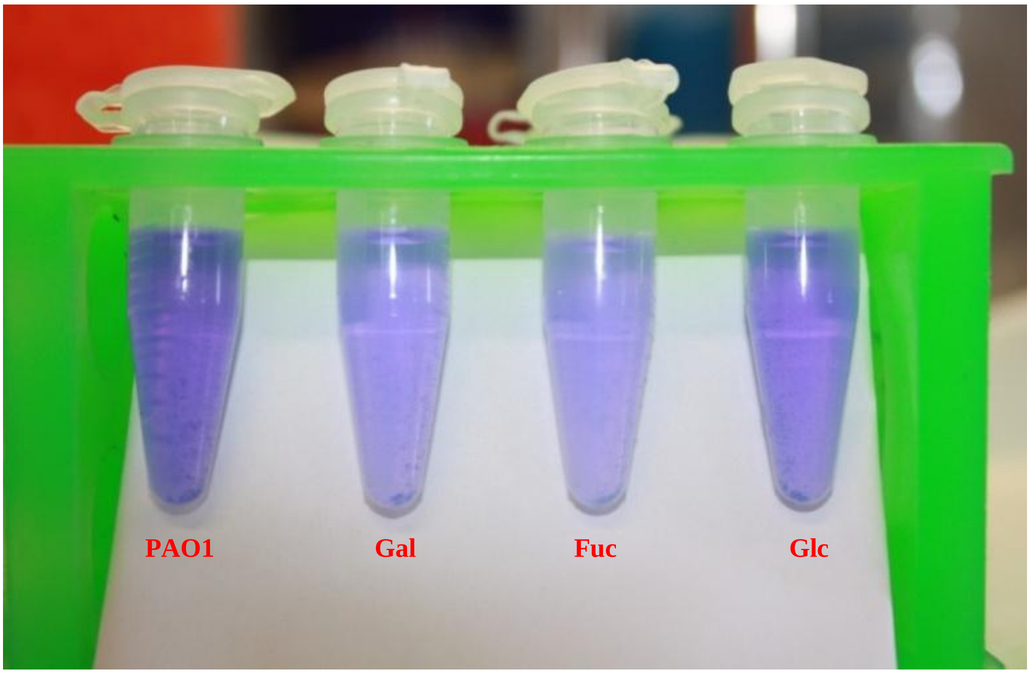
PAO1
Gal
Fuc
Glc
